# The Ypresian ichthyofauna of the Monte Solane *Lagerstätte* (Verona, northern Italy): A deep dive into the western Tethys early Eocene mesopelagic setting

**DOI:** 10.1371/journal.pone.0338490

**Published:** 2026-03-04

**Authors:** Pietro Calzoni, Luca Giusberti, Eliana Fornaciari, Valeria Luciani, Flavia Boscolo-Galazzo, Massimo Bernardi, Riccardo Tomasoni, Giorgio Carnevale

**Affiliations:** 1 Dipartimento di Geoscienze, Università degli Studi di Padova, Padova, Italy; 2 Dipartimento di Fisica e Scienze della Terra, Università degli Studi di Ferrara, Ferrara, Italy; 3 Bremen University, MARUM, Center for Marine Environmental Sciences, Bremen, Germany; 4 MUSE, Museo delle Scienze, Trento, Italy; 5 Dipartimento di Scienze della Terra, Università degli Studi di Torino, Torino, Italy; Indian Institute of Technology (IIT) - Bombay, INDIA

## Abstract

The Ypresian (lower Eocene) fish-bearing site of Monte Solane (Verona province, northern Italy) was discovered in the early 2000s, and its stratigraphy was thoroughly investigated more than a decade ago. However, its ichthyofauna, largely dominated by mesopelagic bony fishes, remained largely unstudied, even though it represents the second richest assemblage in the Verona province, ranking immediately after the Bolca *Lagerstätten*, which are globally renowned for their exceptionally abundant and diverse reef-associated fish assemblages. The scientific relevance of Monte Solane resides in being, along with the Solteri *Lagerstätte* (Trento province, northern Italy), the nearly coeval bathyal equivalent of Bolca. The Monte Solane ichthyofauna, represented by almost 200 collected specimens, comprises what reflects a mesopelagic assemblage including stomiiforms (Gonostomatidae and Phosichthyidae), myctophiforms (Myctophidae), and various percomorphs (Euzaphlegidae, Gempylidae, and Trichiuridae), plus a few more groups usually linked to the coastal epipelagic or benthic habitats (e.g., Clupeiformes and Apogonidae). Several new taxa are established herein: *Acanthophleges lessiniae* n. gen. et n. sp., *Bolcaichthys solanensis* n. sp., *Contemptor mastinoi* n. gen. et n. sp., *Eomastix zabimaru* n. gen. et n. sp., *Lepidoclupea renga* n. gen. et n. sp., *Sabbathichthys osbournei* n. gen. et n. sp., *Thyrsitoides cangrandei* n. sp., and *Veronaphleges ambrosii* n. sp. The stratigraphic relationships of the Monte Solane and Solteri sections are also investigated, and their assemblages are compared to better understand the main features of the Eocene mesopelagic environments of the western part of the Tethys. Exploring these sites can help to unveil the paleontological characters of the most ancient known Cenozoic deep-water *Lagerstätten* known, and also to better define the structure of the pelagic fish communities during the demise of the Early Eocene Climatic Optimum (EECO; ~ 53.2–49.1 Ma), the interval of the Cenozoic with the warmest long-term global average temperature and highest CO_2_ levels.

## Introduction

**Fig 1:**
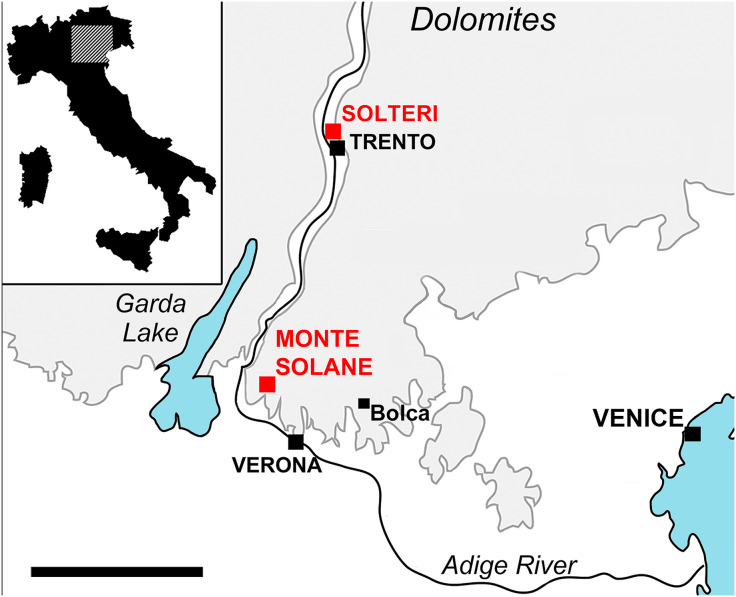
Sketch map of northeastern Italy showing the location of the Bolca, Monte Solane, and Solteri *Lagerstätten.* Scale bar 50 km. Map modified and reprinted from [14] under a CC BY license, with permission from Rivista Italiana di Paleontologia e Stratigrafia, original copyright year 2025.

Among Italian fish-bearing Paleogene deposits, the Veneto region (northeastern Italy) stands out for yielding the highest diversity, especially thanks to the celebrated Bolca *Lagerstätten* (Verona and Vicenza provinces), consisting of about 250 bony fish species belonging to about 200 genera [1,2]. Other remarkable Paleogene fish-bearing deposits are known from the region (e.g., Perarolo; [3]), but some of them remain poorly studied or insufficiently described constraining reconstructions of the Paleogene paleoichthyodiversity in this portion of the western Tethys (e.g., the Chiavon historical site, Vicenza province; [4]). Many other Eurasian early Paleogene fish-bearing sites are documented, showing exceptional preservation, such as the lower Ypresian Fur Formation deposits in Denmark [5,6] and Danata Formation in Turkmenistan [7–10], and the lower Eocene London Clay Formation in England [11,12]. However, all these sites reflect shallow-water environments and epipelagic contexts, with deep-sea localities being less common but crucial for better understanding the structure and composition of the Paleogene Tethyan fish faunas [13]. Recently, a new mesopelagic fish assemblage was reported from Solteri, in the Trento province of northern Italy. The site preserves an ichthyofauna dominated by deep-sea taxa, which has significantly extended the known stratigraphic ranges and geographic distributions of several families, including the stomiiform Phosichthyidae and Gonostomatidae [14]. Ca. 63 km southwest of the Solteri site, an Eocene *Lagerstätte* was discovered in the early 2000s at Monte Solane, in the western Lessini Mountains (Verona Province, northeastern Italy; Fig 1). However, this site has received so far little attention beyond stratigraphic investigations, which assigned the fish-bearing layer to the upper Ypresian [15]. Monte Solane yields the second richest Cenozoic ichthyofauna from the Verona province, second only to the Bolca *Lagerstätten*, to which it is not only geographically close but also stratigraphically almost coeval, being one of the oldest Cenozoic pelagic sites known worldwide (early Eocene, late Ypresian [15]). The early Eocene time frame is particularly relevant since it falls between the aftermath of the K/Pg event, when a remarkable radiation of teleost fishes took place, and the end of the Early Eocene Climatic Optimum (EECO; ~ 53.2–49.1 Ma), one of the warmest periods of the Cenozoic, which was impactful on all marine diversity worldwide [16]. Here we provide a comprehensive description of the lower Eocene ichthyofauna from the Monte Solane site, to assess its paleoenvironmental significance, and to compare it with the potentially coeval and geographically close bathyal site of Solteri [14,17,18]. To this end, we present a high-resolution stratigraphic framework for the Solteri section, integrating data from calcareous nannofossils and planktic foraminifera.

## Geological setting

All the fossils examined in this study come from the Monte Solane site, western Lessini Mountains, Verona province. The Lessini Mountains are a sector of the Southern Alps in northern Italy. During the Paleogene, this area was located on the “Lessini Shelf” [19], a paleogeographic element superimposed on the Trento Platform, a Jurassic structural high and paleogeographic domain of the Adria promontory. This platform drowned completely since the Middle Jurassic, when it was draped by pelagic sediments and became the Trento Plateau, a complex structural high characterized by reduced sedimentation (e.g., [20]). The Trento Plateau reacted rigidly during the eoalpine collision and was segmented by uplifted blocks that acted, since the early Eocene, as centers of initiation of shallow-water carbonates (e.g., Torbole Limestone), which eventually coalesced to form the Lessini Shelf [19,21,22]. Such growing small platforms shed carbonatic biodetritus into the surrounding depressed areas, as testified by slope deposits that graded into the basinal sediments of the Chiusole Formation (e.g., Malcesine Limestone; [21]). The Chiusole Formation basically represents most of the stratigraphical succession of Monte Solane. The lower portion of this formation is faulted, in direct contact with the Cretaceous Scaglia Variegata Alpina [15]. The section starts above the fault with fine-grained whitish or light brown cherty calcarenites, sometimes grading into dark marly limestones, in which metrical banks of coarse, larger foraminiferal-rich biocalcirudites are intercalated. The total thickness of the Chiusole Formation at Monte Solane probably exceeds 80 m, and its uppermost portion mostly consists of alternations of fine whitish calcarenitic limestones and coarse nummulitic biocalciruditic limestones containing micritic clasts and sparse silicified colonial corals. The section and fossiliferous site crop out in a small valley on the western front of the mountain, hidden between the hills north of the Sant’Ambrogio di Valpolicella village. The fossiliferous fish-bearing bed, a ca. 30 cm-thick laminated grey limestone, was first discovered in the early 2000s [23–25]. All fossils have been retrieved thanks to individual sampling, and there has not been any official excavation. The most abundant fossils retrieved from the site are bony fish skeletons and, subordinately, vegetal remains, mostly represented by marine macroalgae (*Delesserites*), a few angiosperm leaves and fruits (*Dombeyopsis* and *Chaneia*), and indeterminate seeds. Lastly, only a handful of poorly preserved crustacean remains (Brachyura and Caridea indet.) are known from the sites ( [15] and present paper). The Monte Solane section was investigated in detail from a biostratigraphic perspective, which allowed the fossiliferous bed to be referred to the upper Ypresian planktic foraminiferal E7a and calcareous nannofossil NP 13 Zones [15].

## Materials and methods

### Fishes

The material examined in this study includes 199 fossil specimens of bony fishes. The specimens are mainly housed in the collections of the Museo di Storia Naturale di Verona, Verona (Italy; 78 specimens), Museo della Natura e dell’Uomo of the University of Padova (Italy; 110 specimens), and Antiquarium of San Giorgio di Valpolicella (Verona province, Italy; 11 specimens). The specimens were never properly studied before. Photos of the specimens were taken using a Sony α7R3 camera, mounting a Sony FE 2.8/90 mm macro G OSS lens. Various specimens were photographed coated with alcohol to emphasize the osteological details by creating a distinct contrast in color with the surrounding matrix. Counts and measurements were taken directly on the specimens using a digital caliper. The specimens were studied with a stereomicroscope, Wild Heerbrugg M5, equipped with a camera lucida drawing arm.

### Nomenclatural acts

The electronic edition of this article conforms to the requirements of the amended International Code of Zoological Nomenclature, and hence the new names contained herein are available under that Code from the electronic edition of this article. This published work and the nomenclatural acts it contains have been registered in ZooBank, the online registration system for the ICZN. The ZooBank LSIDs (Life Science Identifiers) can be resolved and the associated information viewed through any standard web browser by appending the LSID to the prefix ““http://zoobank.org/”“. The LSID for this publication is: urn:lsid:zoobank.org:pub:D2CD75E3-170E-47 AC-9979–384503DF93FD. The electronic edition of this work was published in a journal with an ISSN and has been archived and is available from the following digital repositories: PubMed Central, LOCKSS.

### Micropaleontological and stratigraphical investigation of the Solteri site

#### Sampling.

The original profile of the bathyal fish-bearing site of Solteri (Trento, northern Italy), as originally described in [17] and located ca. 63 km NE of the Monte Solane site (Fig 1), is still outcropping, even if most of the originally described section is covered by protective wire mesh, as reported in [14]. In 2014, three of us (LG, MB, and RT) successfully sampled at Solteri a ca. 7-meter-thick interval of the original profile of Venzo et al. [17] that includes, in its lower half, the fossiliferous, fish-bearing level (S1 Fig). Within this segment, a total of 34 samples were collected with an average spacing of ca. 20 cm. At that time, the sampling was aimed at precisely dating this bathyal site through micropaleontological investigations and comparing it with the companion, biostratigraphically well-constrained Monte Solane site [15]. The results are presented here for the first time, together with our proposed stratigraphic correlation with the Monte Solane site.

#### Carbonate analysis.

The CaCO_3_ content of the lithologies of the Solteri section was measured on 34 samples with the electronic gas volumetric GEO-RS Calcimeter (analytical precision of ± 0.2%) at the University of Ferrara. All the samples were dried, crushed, and then pulverized. Calcimetric analyses were performed on 0.50 g (±0.001 g) of pulverized samples through a reaction derived from the treatment with 5 ml of hydrochloric acid solution (HCl) diluted to 10%. Carbonate weight percentage was obtained as a function of the increase in pressure due to the release of carbon dioxide (CO_2_), according to the following relation: CaCO_3_ + 2HCl → Ca^2+^ + 2Cl^-^ + H_2_O + CO_2_. Every 10 determinations, the instrument was calibrated using 0.500 g of pure Carlo Erba RPE calcium carbonate powder as a standard.

#### Planktic foraminifera.

The planktic foraminiferal content of Solteri was analyzed for ten samples (SLT 1, 2, 3, 4, 5, 6 bis, 7, 15, 22, and 30; S1 Table). Planktic foraminifera dominate the foraminiferal assemblages being >90% in all the samples studied, thus indicating an upper bathyal depositional setting, similarly to the Solane section [15]. Semiquantitative analysis was performed in washed residues of the > 63 μm fraction obtained from limestones, marly limestones, and indurate marls processed following the cold acetolysis method of Lirer [26] as described in Luciani et al. [27]. The planktic foraminiferal zonation for the Eocene here adopted is based on the zonal scheme of Wade et al. [28]. However, Luciani & Giusberti [29] highlighted diachronisms among some early Eocene zonal markers. Specifically, the base of *Acarinina cuneicamerata,* previously adopted to identify the base of Zone E7a and believed to occur above the *Morozovella subbotinae* top, datum that characterized the E5/E6 zonal boundary, occurs in reverse order with respect to the Wade et al. [28] zonation, thus making Zone E6 invalid as previously defined. This reversed order appears to be globally recorded as it has been observed in the Atlantic, tropical Pacific, and southern Indian Oceans [16,30–33]. Luciani & Giusberti [29] proposed an alternative definition of the E6/E7a zonal boundary by replacing the base of *A. cuneicamerata* with the base of *Astrorotalia palmerae,* as the base of the latter species is considered to coincide with the *A. cuneicamerata* base and occurs above the *M. subbotinae* base [34,35].

#### Calcareous nannofossils.

Thirty samples (S1 Table) were prepared as smear slides from unprocessed material and examined under a light microscope at a magnification of 1250 × . A preliminary qualitative assessment was performed to evaluate the abundance and preservation state of the calcareous nannofossil assemblages. Subsequently, the semiquantitative and quantitative counting methods proposed by Gardin & Monechi [36] and Rio et al. [37] were utilized to determine the presence or absence of index species. These methods included: 1) counting specimens of biostratigraphically significant taxa in an area of approximately 6–7 mm², roughly equivalent to three vertical traverses (modified after [36]) 2) counting a predetermined number of specimens of taxonomically related forms, such as *Coccolithus* and *Discoaster*. This approach was applied only when the count of specimens of the selected taxon (i.e., the genus) reached a statistically significant threshold of 10–100 specimens. The taxonomy used is based on the works of [38–40] for the genus *Tribrachiatus*. The calcareous nannofossil zonation adopted is from Agnini et al. [41].

### Institutional abbreviations

IGVR: General inventory of the Verona province (Museo di Storia Naturale di Verona and Antiquarium of San Giorgio di Valpolicella, Verona, Italy); MGP-PD: Museo della Natura e dell’Uomo of the University of Padova, Padova, Italy.

## Results

### Stratigraphic framework of the Solteri section and comparison with the Monte Solane site

#### Lithology and CaCO_3_ analysis of Solteri.

The 690 cm-thick sampled profile at Solteri can be subdivided into three main lithological intervals (S1 Fig):

- Interval L1. The basal interval, ca. 120 m thick (S1 Fig), likely corresponds to the top of the 2 m-thick “member XIII” of Venzo et al. [17] and is mainly represented by grey and light grey bioturbated limestones (CaCO_3_ varying from 81 to 93% with a drop to 65% at the top) with sparse sulfide nodules. Such a basal interval is marked at the top by a 2–7 cm thick level with chert.
- Interval L2. It consists of a package, ca. 260 cm thick, of dark grey to black limestones and marls, frequently laminated with abundant sulfides and locally with plants and undetermined?phosphatic remains. Its lower portion, ca. 1 m-thick, is represented by dominantly black and thinly laminated marls and calcareous marls (the CaCO_3_ analysis records two drops at 53 and 41%); it likely corresponds to “member XIV” of Venzo et al. [17], the fish-bearing interval. The overlying 160 cm are dominantly grey marly limestones and calcareous marls, unevenly laminated, in which a drop in CaCO_3_ is recorded (52%) at ca. 280 cm from the base section. At 257 cm from the base section, a marked undulated contact has been observed. Bioturbation consistently reappears at the top of the interval. This portion of interval L2 likely corresponds to the base of the “member XV” of Venzo et al. [17].
- Interval L3. It is ca. 320 cm thick (“member XV” p.p. of Venzo et al. [17]) and is mostly represented by light grey and light brown bioturbated limestones with some thin laminated intervals. Sulfide nodules are still present. The average CaCO3 percentage is 89% with a drop to 72% at ca. 583 cm, corresponding to a thin, dark grey laminated interval.

#### Planktic foraminifera.

**Fig 2:**
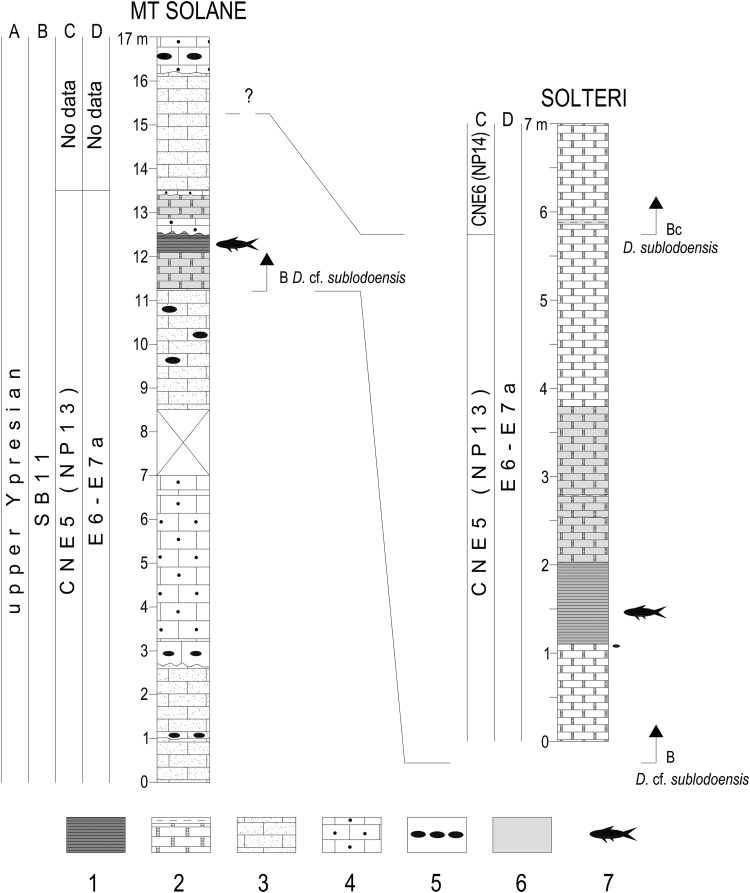
Correlation between the Ypresian Monte Solane (Verona) and Solteri (Trento) fish-bearing *Lagerstätten.* A: chronostratigraphy; B: larger foraminiferal zonation SB after Serra-Kiel et al. [42]; C: calcareous nannofossil zonation CNE after Agnini et al. [41] and equivalent zonation NP of Martini [43]; D: planktic foraminiferal zonation E after Wade et al. [28], modified by [29]. Lithologic legend: 1) laminated, organic-rich marls and calcareous marls; 2) limestones, marly limestones and marls; 3) calcarenitic limestones; 4) calciruditic larger foraminiferal limestones; 5) chert; 6) dark grey to brown lithologies; 7) fishes.

The disaggregation of the Solteri samples provided isolated foraminifera generally not well preserved; however, the planktic foraminiferal species are appropriately recognizable for biostratigraphic purposes. Assemblages are constituted by typical Ypresian taxa, dominated by acarininids (*Acarinina coalingensis, A. cuneicamerata, A. bullbrooki*, and *A. primitiva),* with rare subbotinids, and morozovellids (*Morozovella aragonensis, M. crater*, and *M. formosa*). The species *Morozovella subbotinae* is absent from the basal sample analyzed, and the species *Astrorotalia palmerae* was not recorded. Therefore, we combine the E6-E7a Zones, to which is referable the entire interval of the sampled Solteri section (Figs 2 and S1), in the absence of *Turborotalia frontosa,* which base marks the E7a/E7b zonal boundary.

#### Calcareous nannofossils.

All samples analyzed contain a few to rare calcareous nannofossils, exhibiting a poor to moderate state of preservation. In particular, the calcareous nannofossil assemblages in the basal 70 cm of the section are significantly fragmented (S1 Table). The main results are presented in S1 Fig, and S1 Table. Among the placoliths, the dominant taxa belong to the genus *Coccolithus*, which includes *Coccolithus pelagicus*, *Coccolithus crassus*, and an integrated morphotype of these two species. Notably, the distribution pattern of *Coccolithus*, especially *Coccolithus crassus*, mirrors the profile of CaCO_3_%. The Noelaerhabdaceae including *Cyclicargolithus floridanus*, *Dictyococcites*, and *Reticulofenestra*, follow in terms of abundance, while the genus *Toweius* is generally scarce to rare. The genus *Discoaster* exhibits consistently low abundance (S1 Table). Among the discoasterids, the absolute abundance counts indicate that *Discoaster lodoensis* is present in most samples. The continuous presence of pristine specimens of *Discoaster sublodoensis*, though in modest abundance, characterizes the last 1.30 meters of the sampled Solteri section (Fig 2). The presence of five-rayed *Discoaster* with ambiguous taxonomic and morphological features (*Discoaster* cf. *sublodoensis*) complicates the confident identification of *D*. *sublodoensis* below the uppermost 1.30 meters of the section (S1 Fig and S1 Table). However, in samples STL6, STL13, and STL22 (at 117, 279, and 480 cm, respectively), single or rare specimens of *D. sublodoensis* are documented (S1 Table). The single specimen of *Tribrachiatus orthostylus* observed at the 2.37 m level (sample STL11; S1 Fig) has been attributed to reworking. In agreement with [41], the distribution patterns of Noelaerhabdaceae, *Discoaster lodoensis*, and *Toweius*, coupled with the virtual absence of *T. orthostylus* and the sporadic presence of *D. sublodoensis* (S1 Fig and S1 Table), allow us to assign the first 5.70 meters of the Solteri section to calcareous nannofossil Zone CNE5 of Agnini et al. [41]. Specifically, we assign this portion of the Solteri section to the upper CNE5 Zone due to the presence of rare and sporadic specimens of *D*. *sublodoensis* in agreement of Agnini et al. [44]. Conversely, the upper portion of the section can be attributed to Zone CNE6 of Agnini et al. [41] due to the consistent presence of *D. sublodoensis* (Figs 2 and S1).

#### Age of the Solteri *Lagerstätte* and correlation with the Monte Solane site.

The abundance of calcareous plankton in the Solteri section allows us to establish the age of the fish-bearing horizon and a comparison with the biostratigraphic framework of the Monte Solane section [15] (Fig 2). To enable a reliable biostratigraphic comparison between the two sites, the zonal assignments for Monte Solane, as reported by Giusberti et al. [15], have been updated based on the most recent zonal schemes. Specifically, Giusberti et al. [15] referred the Monte Solane section to the planktic foraminiferal Zone E7a, following the Wade et al. [28] zonation, due to the occurrence of *A. cuneicamerata* and absence of both *M. subbotinae* and *Turborotalia frontosa*. Considering that Luciani & Giusberti [29] redefined the E6/E7a zonal boundary by adopting the top of *M. subbotinae*, we assigned herein the Monte Solane site to the combined E6-E7a Zone. As for the calcareous nannofossil biostratigraphy, Giusberti et al. [15] assigned the Monte Solane site to the Zone NP13, based on the zonation of Martini [43]. Because in the zonal scheme of Agnini et al. [41] the Zone NP13 corresponds to the CNE5, both defined as the interval from the top of *T. orthostylus* and the Base common of *D. sublodoensis,* we refer here the Monte Solane site to the Zone CNE5 (Fig 2). Specifically, the laminated fish bed of Monte Solane deposited in the upper part of this zone [15]. In addition, we further constrain the Monte Solane calcareous nannofossil biostratigraphy to correlate even more precisely the fish beds of Monte Solane and Solteri. Specifically, at Monte Solane, approximately 100 cm below the base of the fish bed, and at Solteri, from the base of the section, we observe the presence of *Discoaster* cf. *sublodoensis,* a morphotype of a five-rayed *Discoaster* that shares the same morphological characteristics as *D. sublodoensis* but is smaller in size. The presence of *D.* cf. *sublodoensis* is not documented in existing literature; however, its occurrence in both sections serves as a valuable tool in the local context for better correlating and constraining the fish-bearing laminites of Solteri and Monte Solane. As a result, both fish-bearing laminites occur after the first occurrence of *D*. cf. *sublodoensis* and below the lowest common occurrence of *D. sublodoensis* (Fig 2) that marks the base of the CNE6 Zone of Agnini et al. [41]. At Solteri, we record the base of the Zone CNE6 ~ 380 cm above the top of the fish-bearing interval. It should be noted that in Solane, the last 3.5 meters of the section consist of calcarenitic and calciruditic lithologies that are unsuitable for the study of calcareous plankton; thus, we cannot establish where the *Discoaster sublodoensis* Bc (the CNE5/CNE6 boundary) occurs. The results of our revision imply that the fish-bearing beds at both sites correspond to the upper Ypresian calcareous nannofossil Zone CNE5 and the combined planktic foraminiferal Zone E6-E7a (Fig 2). This correspondence is the same as reported in the integrated zonal scheme of Speijer et al. [45].

### Systematic paleontology

Class Chondrichthyes Huxley, 1880 [46]

Subclass Elasmobranchii Bonaparte, 1838 [47]

Cohort Euselachii Hay, 1902 [48]

Subcohort Neoselachii Compagno, 1970 [49]

Superorder Squalomorphii Compagno, 1973 [50]

Order Carcharhiniformes Compagno, 1973 [50]

Fam. indet.

Fig 3

**Referred materials:** IGVR MSNV 82454, an isolated tooth with crown and root preserved.

#### Description.

**Fig 3:**
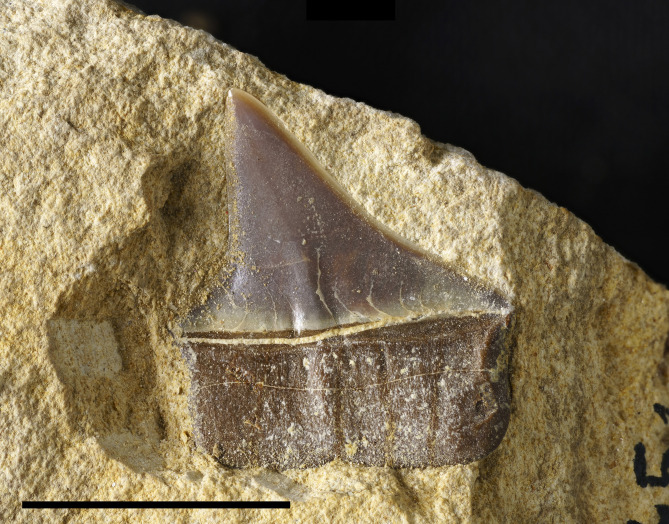
Carcharhiniformes Fam. indet. Single isolated tooth exposed on the labial side. Scale bar 10 mm.

This single isolated tooth is characterized by a triangular shape and a labial-lingual compressed crown with smooth edges. The crown is still embedded in the matrix, exposed on the labial side. The mesial cutting edge is gradually inclined, with a gentle inclination at its base near the heel and becoming steeper at half height, while the distal cutting edge is straight and vertical with a gently rounded and high heel at its base. The cutting edge at the distal heel is apparently irregular and incised, but it cannot be verified since it is partially covered by the matrix. The root is deep, half the height of the crown, and rectangular in shape, with a shallow nutrient groove at its base and numerous foramina concentrated in the upper portion. In the middle of the base of the crown and in the middle of the root, there is a gentle ridge, bordered by two shallow plications at the level of the crown (Fig 3).

#### Discussion.

The tooth morphology is clearly different from that of *Eogaleus bolcensis* from Bolca, which exhibits a slender cusp and incision at the distal heel [51]. Moreover, the nutrient groove on the tooth from Monte Solane is shallower as seen from the labial side compared to that of *Eogaleus* [51]. The described tooth is provisionally attributed to an indeterminate carcharhiniform because of the crown and root aspect and presence of an evident nutrient groove and foramina on the root. Due to the lingual side being covered by the matrix, it is not possible to unambiguously assign it to a specific family.

Class Actinopterygii Cope, 1887 [52]

Infraclass Teleostei Müller, 1845, *sensu* Arratia, 1999 [53]

Superorder Clupeomorpha Greenwood et al., 1966 [54]

Order Clupeiformes Bleeker, 1859 [55]

Suborder Clupeoidei Bleeker, 1859 [55]

Genus *Bolcaichthys* Marramà & Carnevale, 2015 [56]

**Type species:** *Bolcaichthys catopygopterus* Marramà & Carnevale, 2015 [56]

**Emended diagnosis:** Small- to medium-sized clupeoid unique for the following combination of characters: elongated and tapered body; head length approximately one third to one quarter SL; skull roof with 10−14 frontoparietal striae; mouth terminal; two supramaxillae, anterior small and rod-like, and posterior paddle-shaped; teeth absent in jaws and palate; complete series of abdominal keeled scutes (10 or 11 prepelvic, 10 or 11 postpelvic) with ascending arms; dorsal scutes absent; five or six branchiostegal rays; eight supraneurals; 40−43 vertebrae; 20−23 ribs; ribs-vertebrae ratio 0.48–0.55; three epurals; deeply forked caudal fin with 19 principal caudal-fin rays; dorsal fin small, inserting at about mid-length of the body with 13–16 rays; 15 or 16 anal-fin rays; 10–18 pectoral-fin rays; pelvic-fin origin slightly in front of or behind the posterior end of the dorsal fin and seven or eight pelvic-fin rays.

**Fig 4:**
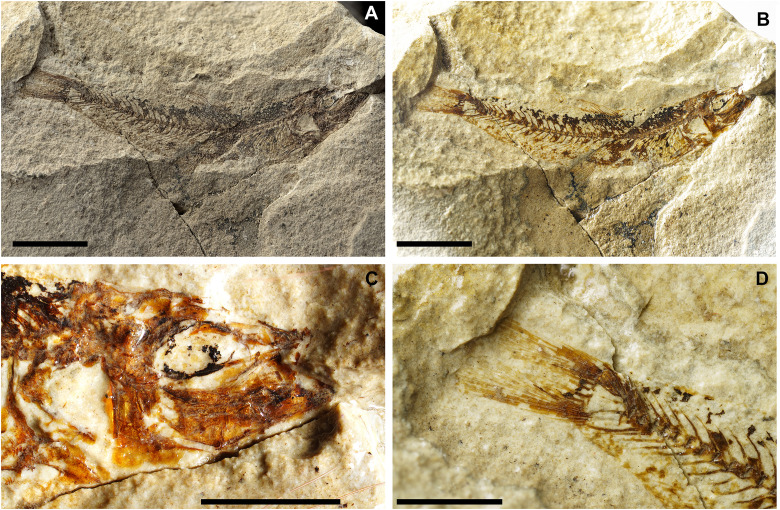
*Bolcaichthys solanensis* n. sp. Lateral view of the holotype, MGP-PD 33556, in natural light **(A)** and coated in alcohol **(B-D)**. Details of the cranium **(C)** and of the caudal fin **(D)**. Scale bars 10 mm **(A-B)**; 5 mm **(C-D)**.

**Fig 5:**
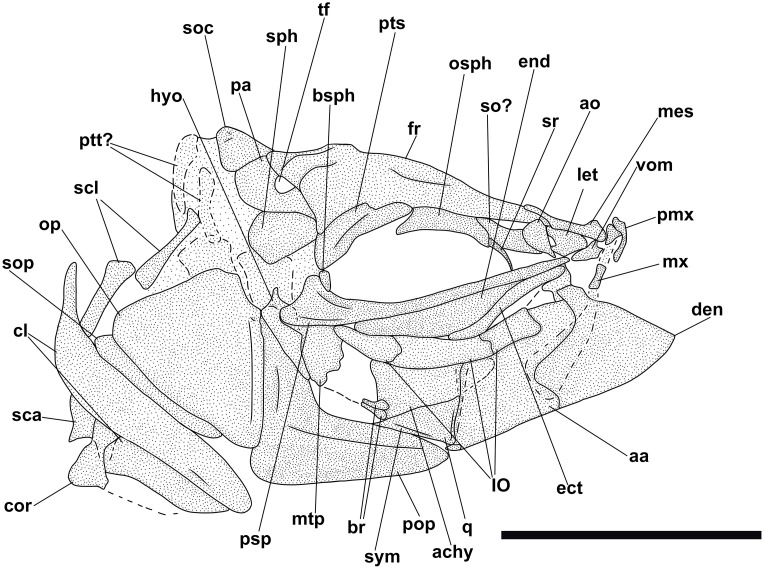
*Bolcaichthys solanensis* n. sp. **Holotype, MGP-PD 33556, interpretive reconstruction of the cranium.** Scale bar 5 mm. Abbreviations: aa: anguloarticular; ao: antorbital; achy: anterior ceratohyal; br: branchiostegal rays; bsph: basisphenoid; cor: coracoid; cl: cleithrum; den: dentary; ect: ectopterygoid; end: endopterygoid; fr: frontal; hyo: hyomandibula; IO: infraorbital bones; let: lateral ethmoid; mes: mesethmoid; mtp: metapterygoid; mx: maxilla; op: opercle; osph: orbitosphenoid; pa: parietal; pmx: premaxilla; pop: preopercle; psp: parasphenoid; pts: pterosphenoid; ptt: posttemporal; q: quadrate; sca: scapula; scl: supracleithrum; so: supraorbital; soc: supraoccipital; sop: subopercle; sph: sphenotic; sr: sclerotic ring; sym: symplectic; tf: temporal fossa; vom: vomer.

**Fig 6:**
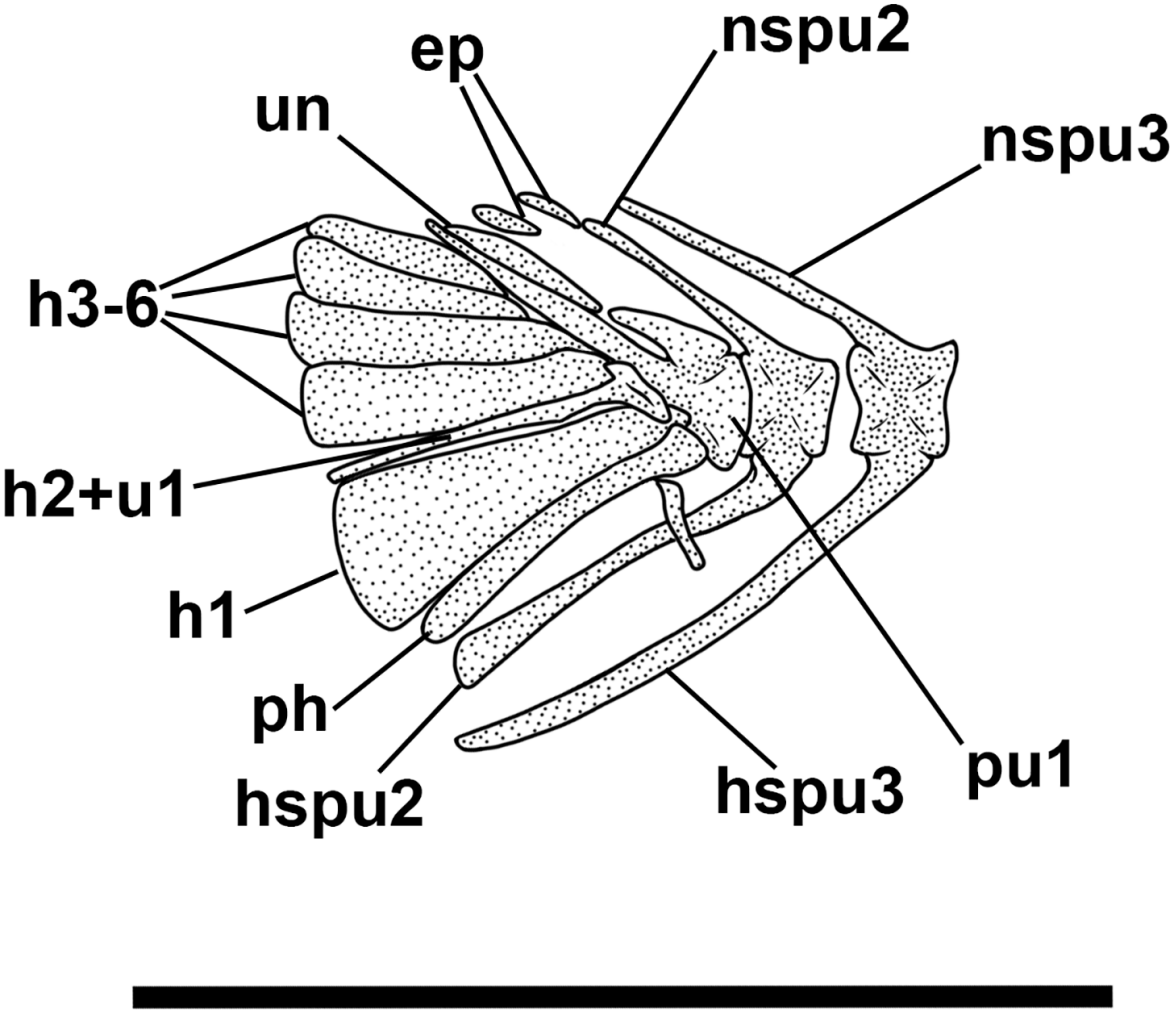
*Bolcaichthys solanensis* n. sp. **Holotype, MGP-PD 33556, interpretive reconstruction of the caudal skeleton.** Scale bar 5 mm. Abbreviations: ep: epural; h: hypural; hspu2: haemal spine of the second preural vertebra; hspu3: haemal spine of the third preural vertebra; nspu2: neural spine of the second preural vertebra; nspu3: neural spine of the third preural vertebra; ph: parhypural; pu1: first preural centrum; u1: first ural centrum; un: uroneural.

*Bolcaichthys solanensis* n. sp. Calzoni, Giusberti & Carnevale

lsid:zoobank.org:act:0B50BEBB-9875-43D0-9DFD-F99999EC6AA6 Figs 4–6

**Diagnosis:** Small-sized species of *Bolcaichthys* characterized by this unique set of traits: head length approximately one fourth of body (HL: 24.4% of SL); 43 vertebrae; 23 ribs; ribs-vertebrae ratio of 0.53; short dorsal fin located at the mid-point of the body with 13 rays; ten pectoral-fin rays; pelvic-fin origin behind the posterior end of the dorsal fin; seven pelvic-fin rays.

**Etymology:** Species named after the type locality “Solane”.

**Type locality and horizon:** Marly limestones of the Chiusole Formation (CNE5 and E6-E7a Zones; upper Ypresian), Monte Solane (Sant’Ambrogio di Valpolicella, Verona, Italy).

**Holotype (by monotypy):** MGP-PD 33556, a nearly complete articulated skeleton, 42.7 mm SL.

#### Description.

The only available specimen of *Bolcaichthys solanensis* n. sp. is small-sized (42.7 mm SL) and exhibits a rather fusiform and slender body (BD: 16.8% of SL; CPH: 7.5% of SL; Fig 4A and 4B; Table 1). The head is moderately large (HL: 24.4% of SL) and so is the orbit (O: 6.4% of SL; Table 1). The dorsal fin inserts around the mid-point of the body, with the pelvic fin inserting slightly behind the end of the dorsal-fin base.

**Table 1:** Measurements of *Bolcaichthys solanensis* n. sp. compared to the type species of the genus *B*. *catopygopterus.*

	*Bolcaichthys solanensis* n. sp.	*Bolcaichthys catopygopterus*
**SL (mm)**	42.7	39-101
**TL (mm)**	46.9	?
**HL**	24.4	26.7-30
**PD**	38.1	41.6-46.4
**PA**	?	5.9-7.2
**PP**	22.7?	28-31
**PV**	58.9	54.8-60
**DFL**	11.2	10.5-12.8
**AFL**	?	13.9-16.2
**PFL**	10.9	?
**VFL**	8.9	?
**PRO**	7.6	?
**O**	6.4	4.3-5.2
**POO**	11.3	11.1-12.8
**DRL**	13.3	?
**AFR**	?	?
**BD**	16.8	20.9-25
**CPL**	?	?
**CPH**	7.5	8.1-9.2

Data from [56]. Values are as percentage of SL. Abbreviations: AFL: anal-fin base length; AFR; anal-fin ray length; BD: maximum body depth; CPH: caudal peduncle height; CPL: caudal peduncle length; DFL: dorsal-fin base length; DRL: dorsal-fin ray length; HL: head length; O: orbit diameter; PA: preanal distance; PD: predorsal distance; PFL: pectoral-fin length; POO: postorbital distance; PRO: preorbital distance; PP: prepectoral distance; PV: prepelvic distance; SL: standard length; TL: total length; VFL: pelvic-fin length.

The neurocranium has a triangular outline. The lateral ethmoid is ovoid-shaped. The mesethmoid is long and slender. The vomer is small and toothless. The frontals are the largest bones of the skull roof: they are thin and narrow anteriorly and broader posteriorly. Due to poor preservation, the frontoparietal striae are not particularly evident in our specimen. A small ovoid temporal fossa is present between the frontal and the parietal, which has a somewhat quadrangular shape (Figs 4C and 5). The sphenotic is subtriangular. The pterotic and the epioccipital are poorly preserved. The supraoccipital is small and triangular. The parasphenoid is straight and narrow, slightly expanded posteriorly. The orbitosphenoid makes up the anterodorsal margin of the orbit, while the pterosphenoid and basisphenoid form its posterior margin.

The nasal is poorly preserved. The supraorbital is rectangular, while the antorbital is almost ovoid-shaped, and a portion of the sclerotic ring is preserved in the anterior part of the orbit. The first infraorbital bone is not preserved, while the second to fourth infraorbitals are trapezoid in shape. The posteriormost infraorbitals are difficult to distinguish.

The mouth is terminal and small, with a narrow gape (Figs 4C and 5). The premaxilla is minute and thin, with extremely reduced processes and lacking teeth. The maxilla is partially preserved: the anterior portion is fragmented, thin, and narrow, while the main body is broader and only visible as an impression. The supramaxillae are not preserved. The dentary is trapezoid, deep, and edentulous. The anguloarticular is robust and deep.

The quadrate is triangular, located under the midportion of the orbit, with a well-developed condyle. The symplectic is thin and small. The ectopterygoid is anteriorly elongate and thin. The endopterygoid is almost triangular, while the metapterygoid is poorly preserved. The palatine is not preserved. The hyomandibula has a broad articular head.

The preopercle is L-shaped and anteroventrally expanded, with horizontal and vertical arms showing similar lengths. The opercle is poorly preserved: it is still possible to notice its broad, trapezoid shape with a curved posterior margin. The subopercle is sickle-shaped and poorly preserved, while the interopercle is not preserved.

The hyoid arch is poorly preserved: the number of branchiostegal rays is difficult to determine, with only two rays articulated with the anterior ceratohyal (Fig 5). The branchial skeleton is not preserved.

There are at least 43 squared and compact vertebrae (22 + 21). The neural arches are elongate, thin, and extend well beyond their respective vertebral centrum. The haemal spines are similar to their neural counterparts. There are 23 pairs of elongate and thin ribs, with the first pair articulated to the third vertebra. Epineurals are present in all but the last 10 vertebrae: they are extremely thin and curved, attached to the base of the neural arches from the second or third vertebra up to the 18^th^ centrum, while they are shorter and become separated in the successive centra, towards the end of the vertebral column. There are poorly preserved epipleurals attached to the first five or six caudal vertebrae.

The haemal spines of the second and third preural vertebrae are thin and fused to their respective centra. The caudal skeleton consists of six autogenous hypurals (except the second hypural, which is fused to the first ural centrum), an autogenous and slender parhypural, two uroneurals (the first fused to the first preural centrum), and two poorly preserved and slender epurals (Figs 4D and 6). The first preural centrum bears a triangular neural plate, and it is fused with the first thin uroneural, while the second ural centrum is not preserved. The caudal fin is forked and contains 19 (10 + 9) principal rays plus at least four dorsal and five ventral procurrent rays (4 + , I, 9 + 8, I, 5).

At least six supraneurals are present, but due to their poor preservation, their exact number is difficult to determine. The dorsal fin inserts around the mid-point of the body (PD: 38.1% of SL; Table 1) and contains 13 rays, with the first two unsegmented and unbranched. The third ray is the longest of the series. The dorsal fin is supported by thin and slender pterygiophores; the first larger and stouter pterygiophore is fragmented. The anal-fin insertion is located behind the end of the dorsal-fin base, in the last third of the body. The anal fin is incomplete and only ten rays and a few thin pterygiophores are preserved.

The posttemporal is poorly preserved and difficult to define. The supracleithrum is rod-like. The cleithrum is large and curved with a well-developed anterior portion. The coracoid is partially preserved. The scapula is small and quadrangular. The mesocoracoid and the postcleithra are not preserved. The pectoral fin contains 10 rays sustained by at least three pectoral-fin radials. The pelvic girdle inserts around mid-point of the body, around the level of the end of the dorsal-fin base. The basipterygium is triangular and slender, lacking any developed process. The pelvic fin contains seven rays.

The ventral abdominal scutes are poorly preserved: their exact number is difficult to determine due to their inadequate preservation. The scutes are moderately well-ossified and lack developed ascending arms, with some pointed dorsal tips being noticeable (Fig 4B and 4D). There are at least four prepelvic and six postpelvic scutes. There are no predorsal scutes.

The squamation is poorly preserved, with just the impressions of minute cycloid scales. There are no lateral line scales.

#### Discussion.

MGP-PD 33556 is referred to the genus *Bolcaichthys* by having: mouth small and terminal, lack of teeth; both pre- and postpelvic abdominal scutes present; dorsal scutes absent; ribs-vertebrae ratio of 0.53 (0.48–0.55 in *B*. *catopygopterus*; [56]; Table 2); caudal fin with 19 principal rays; dorsal fin small, inserted at mid-point of the body; anal fin placed in the posterior third of the body [56].

**Table 2:** Summary of the meristic traits of the different species of the genus *Bolcaichthys.*

	*Bolcaichthys solanensis* n. sp.	*Bolcaichthys catopygopterus*
**Dorsal-fin rays**	13	15-16
**Anal-fin rays**	9+	15-16
**Pectoral-fin rays**	10	14-18
**Pelvic-fin rays**	7	8
**Caudal-fin rays (principal)**	19 (10 + 9)	19 (10 + 9)
**Caudal-fin rays (procurrent)**	4 + 5	?
**Vertebrae**	43 (22 + 21)	40-42 (23-25 + 16-18)
**Ribs-vertebrae ratio**	0.53	0.48-0.55
**Branchiostegal rays**	?	5-6
**Epurals**	2	3

Includes new data and data from [56].

*Bolcaichthys solanensis* n. sp. differs from the type and only species of the genus, *B. catopygopterus*, by having: a more slender body (BD: 16.8% of SL; CPH: 7.5% of SL vs BD: 20.9–25% of SL; CPH: 8.1–9.2% of SL in *B. catopygopterus*; [56]; Table 1); a smaller head (HL: 24.4% of SL vs SL: 26.7–30% of SL in *B. catopygopterus*; [56]; Table 1); 43 vertebrae (vs 40–42 in *B*. *catopygopterus*; [56]; Table 2); 23 ribs (vs 20–22 in *B. catopygopterus*; [56]; Table 2); two epurals (vs three in *B. catopygopterus*; [56]; Table 2); a shorter predorsal distance (PD: 38.7% vs 41.6–46.4% of SL in *B. catopygopterus*; [56]; Table 1); 13 dorsal-fin rays (vs 15–16 rays in *B. catopygopterus*; [56]; Table 2); 10 pectoral-fin rays (vs 14–18 in *B. catopygopterus*; [56]; Table 2) and seven pelvic-fin rays (vs eight in *B. catopygopterus*; [56]; Table 2).

Family Dussumieriidae Gill, 1861 [57]

Genus *Lepidoclupea* n. gen. Calzoni, Giusberti & Carnevale

urn:lsid:zoobank.org:act:3CC8A7C4-E776-4B57-AC0E-4DF02AE3D4CC

**Type species (by monotypy):** *Lepidoclupea* n. gen. et n. sp.

**Diagnosis:** Small-sized Dussumieriidae characterized by the following combination of features: slender and tapered body; head length approximately one fourth of SL (26.3% of SL); mouth terminal with numerous, minute and sharp teeth on both jaws; presence of teeth on the endopterygoid; pre- and postpelvic abdominal scutes absent; 39–40 (17 + 22–23) vertebrae; caudal skeleton with six hypurals, three epurals; and remarkable hypurostegy; deeply forked caudal fin with 19 principal caudal-fin rays; dorsal fin small, inserting at about mid-length of the body, containing 12 rays; anal fin small, inserting in the last fourth of the body, containing 13–16 rays; 10–12 pectoral-fin rays; pelvic-fin origin below the midlength of the dorsal-fin base; seven pelvic-fin rays.

**Etymology:** From the Greek word “*λεπιδωτός*” meaning “scaly” and the Latin word “*clupea*” meaning “herring”.

**Remarks:** This genus is tentatively referred to the Dussumieriidae (in the sense of [58]) by lacking pre- and postpelvic scutes, by having the dorsal-fin origin just anterior to the mid-point of the body, a short anal-fin base placed well posterior to the end of the dorsal fin, and the presence of a peculiar W-shaped scute surrounding the pelvic girdle [59]. The concurrent absence of pre- and postpelvic scutes and presence of teeth excludes the attribution to any of the other clupeoid families [60].

*Lepidoclupea renga* n. gen. et n. sp. Calzoni, Giusberti & Carnevale

urn:lsid:zoobank.org:act:C9D6C9AA-A7FF-447C-8BDF-D343B62EAE0D

Figs 7-9

**Fig 7:**
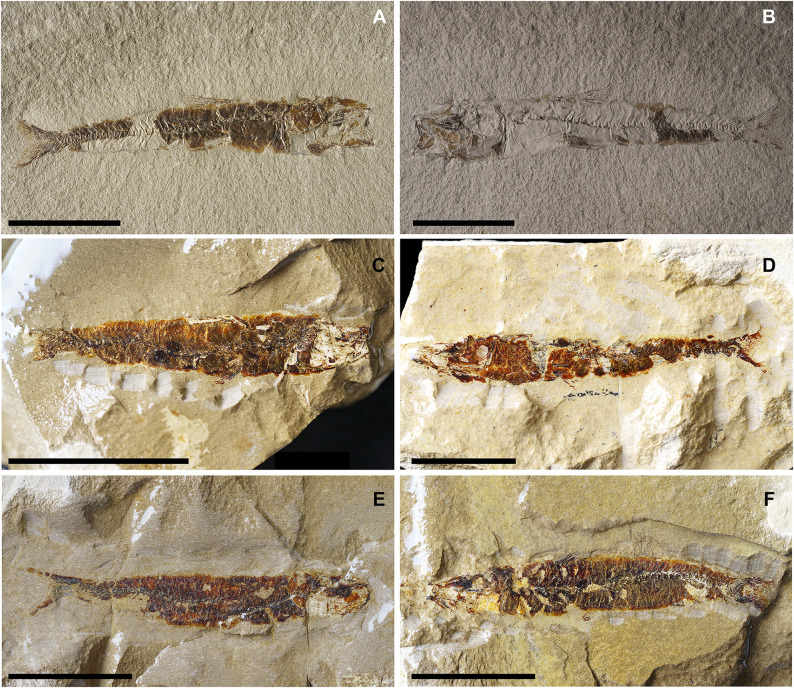
*Lepidoclupea renga* n. gen. et n. sp. Lateral view of the holotype, IGVR 64083-67873 **(A-B)**. Lateral view of the paratypes, coated in alcohol, MGP-PD 33506 **(C)**, MGP-PD 33517 **(D)**, MGP-PD 33535 **(E)**, MGP-PD 33528 **(F)**. Scale bars 20 mm.

**Fig 8:**
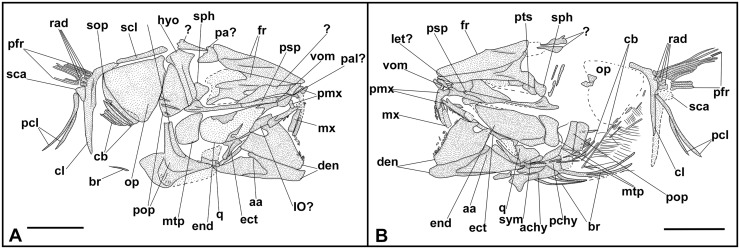
*Lepidoclupea renga* n. gen. et n. sp. Holotype. **Interpretive reconstruction of the cranium.** IGVR 64083 **(A)**, IGVR 67873 **(B)**. Scale bars 5 mm. Abbreviations: aa: anguloarticular; achy: anterior ceratohyal; br: branchiostegal rays; cb: ceratobranchial; cl: cleithrum; den: dentary; ect: ectopterygoid; end: endopterygoid; fr: frontal; hyo: hyomandibula; IO: infraorbital; let: lateral ethmoid; mtp: metapterygoid; mx: maxilla; op: opercle; pa: parietal; pal: palatine; pchy: posterior ceratohyal; pcl: postcleithrum; pfr: pectoral-fin ray; pmx: premaxilla; pop: preopercle; psp: parasphenoid; pts: pterosphenoid; q: quadrate; rad: pectoral-fin radial; sca: scapula; scl: supracleithrum; sop: subopercle; sph: sphenotic; sym: symplectic; vom: vomer.

v2011 Clupeidae gen. indet., Zorzin et al., p. 61, fig. 4. [25]

v2014 Clupeidae gen. indet., Giusberti et al., p. 6, fig. 4A. [15]

**Diagnosis:** As for genus.

**Etymology:** Species named after the Veronese dialect word “*renga*” meaning “herring”.

**Type locality and horizon:** Marly limestones of the Chiusole Formation (CNE5 and E6-E7a Zones; upper Ypresian), Monte Solane (Sant’Ambrogio di Valpolicella, Verona, Italy).

**Holotype:** IGVR 64083–67873, a nearly complete and articulated skeleton, in part and counterpart, 65.7 mm SL

**Paratypes:** MGP-PD 3348, an incomplete articulated skeleton; MGP-PD 33506, a nearly complete articulated skeleton; MGP-PD 33517, a nearly complete articulated skeleton; MGP-PD 33528, a nearly complete articulated skeleton; MGP-PD 33535, a nearly complete articulated skeleton.

#### Description.

*Lepidoclupea renga* n. gen. et n. sp. is a small-sized dussumieriid (65.7 mm SL) with a fusiform and slender body (BD: 17.6% of SL; CPH: 6.7% of SL; Fig 7; Table 3). The head is rather large (HL: 26.3% of SL; Table 3) with a circular orbit. The dorsal fin inserts just anterior to the midlength of the body (PD: 42.7% of SL; Table 3), while the anal-fin origin is placed at the level of the posterior end of the dorsal-fin base (PA: 81.1% of SL; Table 3).

**Table 3:** Measurements of *Lepidoclupea renga* n. gen. et n. sp. compared with the Eocene round herring *Trollichthys bolcensis.*

	*Lepidoclupea renga* n. gen. et n. sp.	*Trollichthys bolcensis*
**SL (mm)**	65.7	74.3-100.1
**TL (mm)**	74.2	107.6-117.6
**HL**	26.3	22.8-28.6
**PD**	42.7	40.3-44
**PA**	81.1	74.8-78.2
**PP**	27.6	23.9-29.3
**PV**	44.2	50.1-62.7
**DFL**	10.9	9.8-13.1
**AFL**	8.1	8.6-16.9
**PFL**	7.6	?
**VFL**	13	?
**PRO**	9	5.7-7.6
**O**	4.3	3.4-4.9
**POO**	14.6	?
**DRL**	15	?
**AFR**	?	?
**BD**	17.6	16.8-24.8
**CPL**	7.1	?
**CPH**	6.7	7.3-8.2

Includes new data and data from [61]. Values are as a percentage of SL. Abbreviations: AFL: anal-fin base length; AFR; anal-fin ray length; BD: maximum body depth; CPH: caudal peduncle height; CPL: caudal peduncle length; DFL: dorsal-fin base length; DRL: dorsal-fin ray length; HL: head length; O: orbit diameter; PA: preanal distance; PD: predorsal distance; PFL: pectoral-fin length; POO: postorbital distance; PRO: preorbital distance; PP: prepectoral distance; PV: prepelvic distance; SL: standard length; TL: total length; VFL: pelvic-fin length.

The neurocranium is triangular (Fig 8A and 8B). The ethmoid region is poorly preserved. The vomer is thin and edentulous. Most of the elements of the skull roof are poorly preserved and difficult to describe. The frontals are large, triangular, and broad with a longitudinal ridge on their surface (Fig 8A and 8B). The sphenotic is almost triangular. The parasphenoid is straight, narrow, and expanded posteriorly. The pterosphenoid is partially exposed, while the basisphenoid is not preserved. The nasals are fragmented and the infraorbital bones are poorly preserved.

The premaxilla is thin and slightly curved, with a short ascending process. It bears at least 16 thin and retrorse teeth (Fig 8B). The maxilla is only partially preserved. The supramaxillae are not preserved. The dentary is deep and triangular: it bears at least 25 small teeth, similar to those of the premaxilla (Fig 8B). The anguloarticular is trapezoid in outline.

The quadrate is fan-like with a developed anterior process and a posterior socket for the insertion of the symplectic (Fig 8B). The ectopterygoid is straight and slightly expanded posteriorly. The endopterygoid is large and almost elliptic and bears numerous teeth on its medial surface (see IGVR 67873; Figs 7B and 8B). The metapterygoid has an irregular shape. The palatine is minute and poorly preserved (Fig 8A). The hyomandibula is displaced from its original position (see IGVR 64083; Figs 7A and 8A) and has an elongate ventral shaft and a broad articular head.

The preopercle is large and L-shaped, ventrally expanded, with the vertical arm slightly longer than the horizontal arm (Fig 8A). The opercle is large and trapezoid with rounded posterior margins and detached from its original position (see IGVR 64083; Figs 7A and 8A). The subopercle is arcuate and partially preserved, while the interopercle is not preserved.

The anterior ceratohyal is narrow, with an expanded anterior end, and lacks a beryciform foramen. The posterior ceratohyal is triangular. There are at least seven thin and elongate branchiostegal rays (Fig 8B). Of the branchial arcs, only isolated and fragmented ceratobranchials are preserved, bearing seven long and spinous gill rakers and numerous gill filaments.

There are 39–40 vertebrae (17 + 22–23), whose centra are almost quadrangular and gradually more compact towards the caudal fin. The neural spines are thin and straight. The haemal spines are slender and slightly curved. There are thin and straight ribs attached to the ventrolateral sides of the abdominal vertebrae up to the 17^th^ vertebra; however, their exact number is difficult to determine due to the dense scales covering the whole body. There are no traces of intermuscular bones.

**Fig 9:**
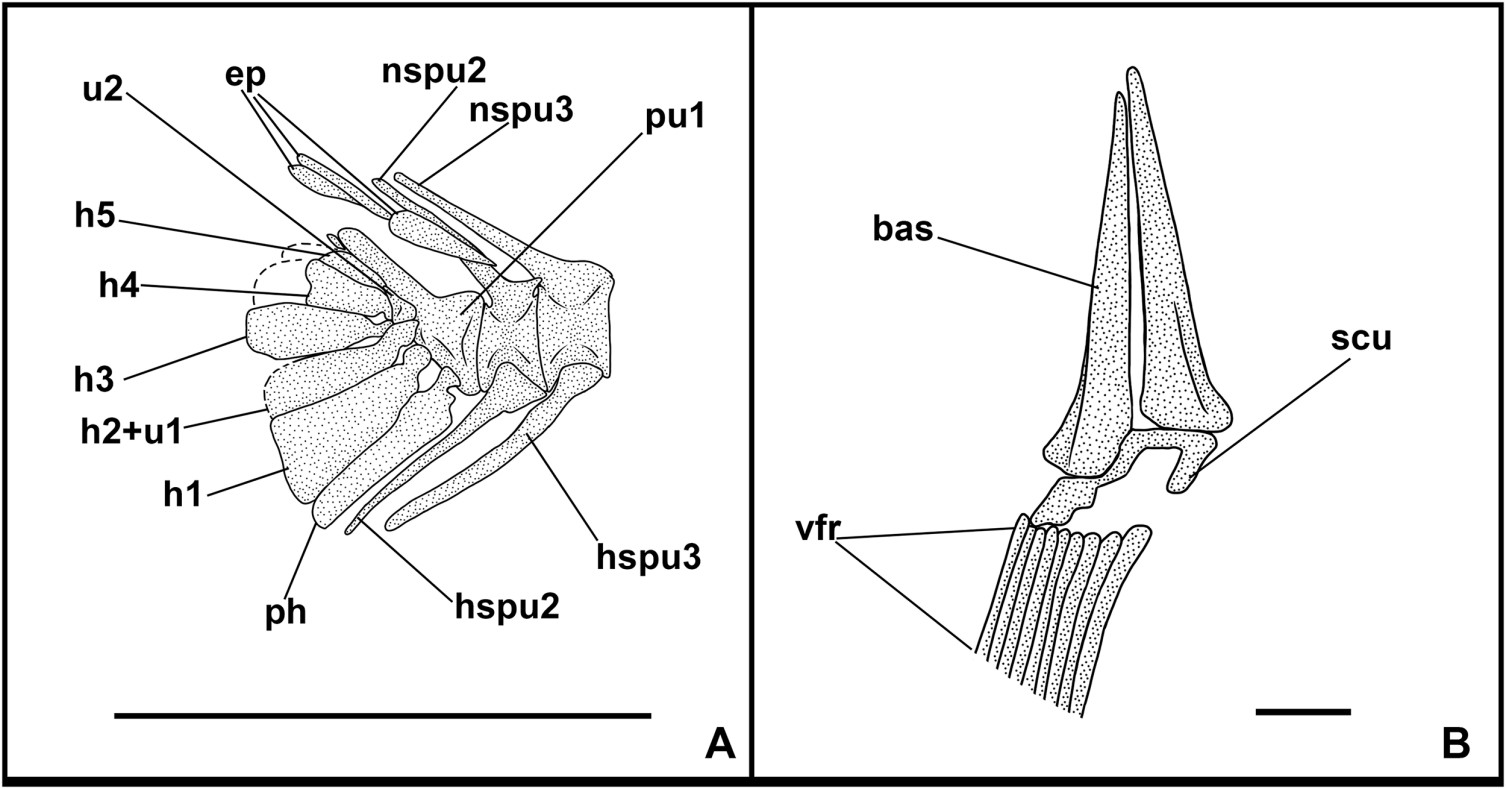
*Lepidoclupea renga* n. gen. et n. sp. **Holotype, IGVR 64083. Interpretive reconstruction of the caudal skeleton (A) and pelvic girdle (B).** Scale bars 5 mm **(A)**; 1 mm **(B)**. Abbreviations: bas: basipterygium; ep: epural; h: hypural; hspu2: haemal spine of the second preural vertebra; hspu3: haemal spine of the third preural vertebra; nspu2: neural spine of the second preural vertebra; nspu3: neural spine of the third preural vertebra; ph: parhypural; pu1: first preural centrum; scu: abdominal scute; u1: first ural centrum; u2: second ural centrum; vfr: pelvic fin rays.

The structure of the caudal skeleton is difficult to determine due to the remarkable degree of hypurostegy. The caudal skeleton seems to consist of at least five hypurals, the second of which fused to the first ural centrum, an autogenous and wedge-shaped parhypural, a single uroneural fused to the first preural centrum, and three poorly preserved epurals (Fig 9A). A second ural centrum is present, albeit reduced. The haemal spines of the second and third preural vertebrae are thin and autogenous (Fig 9A). The caudal fin is forked and contains 19 (10 + 9) principal rays plus eight dorsal and seven ventral procurrent rays (8, I, 9 + 8, I, 7).

Due to inadequate preservation, it is difficult to establish the number of supraneurals. The first dorsal fin originates almost at the midlength of the body (PD: 42.7% of SL; Fig 7; Table 3); it bears 12 rays, of which the 2^nd^ and 3^rd^ are the longest of the series, and it is supported by thin and delicate pterygiophores, whose number is difficult to determine. The anal-fin origin is located well behind the posterior end of the dorsal fin, almost closer to the caudal peduncle; it contains 16 rays supported by thin pterygiophores.

The posttemporal is not preserved. The supracleithrum is thin and rod-like. The cleithrum is large, curved, and anteriorly pointed. The scapula is small and triangular, while the coracoid is not preserved; there are two thin and curved postcleithra, almost reaching the ventral margin of the body (Fig 8A and 8B). The pectoral fin is supported by four pectoral-fin radials and contains 10–12 rays.

The pelvic girdle is located under the dorsal-fin base (PV: 44.2% of SL; Table 3), and the basipterygium is narrow and triangular. The pelvic fin has seven to nine rays.

There are no pre- or postpelvic abdominal scutes, and the peculiar dussumieriid W-shaped pelvic scute is poorly preserved in our material, with some fragments exposed in IGVR 67873, surrounding the anterior portion of the pelvic fins (Figs 7B and 9B).

The whole body is covered by a dense cover of cycloid scales; there is no trace of the lateral-line scales.

#### Discussion.

Giusberti et al. [15] previously ascribed the holotype to Clupeidae gen. indet. However, *Lepidoclupea renga* n. gen. et n. sp. differs from the genera of the Clupeidae by lacking pre- and postpelvic scutes and by having numerous acicular and retrorse teeth (usually absent or not developed in Clupeidae). The only fossil genus clearly referred to the round herrings, based on the presence of the peculiar W-shaped scute, is *Trollichthys bolcensis* from the Ypresian of Bolca, Italy [61].

*Lepidoclupea renga* n. gen. et n. sp. differs from *Trollichthys bolcensis* by having sharp and retrorse teeth in both jaws (vs absent in *T*. *bolcensis*; [61]); presence of teeth on the endopteryogoid (vs absent in *T*. *bolcensis*; [61]); 39–40 vertebrae (vs 41–42 in *T*. *bolcensis*; [61]; Table 4); 19 principal caudal-fin rays (vs 17 in *T*. *bolcensis*; [61]; Table 4); 11 dorsal-fin rays (vs 14–16 in *T*. *bolcensis*; [61]; Table 4) and 12 pectoral-fin rays (vs 14 in *T*. *bolcensis*; [61]; Table 4).

**Table 4:** Summary of the meristic traits of *Lepidoclupea renga* n. gen. et n. sp. compared to those of the round herring *Trollichthys bolcensis.*

	*Lepidoclupea renga* n. gen. et. n. sp.	*Trollichthys bolcensis*
**Dorsal-fin rays**	12	14-16
**Anal-fin rays**	13-16	13
**Pectoral-fin rays**	10-12	14
**Pelvic-fin rays**	7-9	8
**Caudal-fin rays (principal)**	19 (10 + 9)	17 (9 + 8)
**Caudal-fin rays (procurrent)**	8 + 7	?
**Vertebrae**	39-40 (17 + 22-23)	41-42 (19-20 + 21-22)
**Ribs-vertebrae ratio**	?	0.52-0.57
**Branchiostegal rays**	7+	?
**Epurals**	3?	?

Includes new data and data from [61].

Other fossil round herrings include *Dussumieria elami* and *Etrumeus hafizi* from the Eocene of Iran [62] and *Etrumeus boulei* and *Spratelloides lemoinei* from the Upper Miocene of the Mediterranean [63,64].

Order Stomiiformes *sensu* Harold & Weitzman, 1996 [65]

Family Phosichthyidae Weitzman, 1974 [66]

Genus *Sabbathichthys* n. gen. Calzoni, Giusberti & Carnevale

urn:lsid:zoobank.org:act:3CAE0E6A-8773-4E92-BD23-671B4135DD2C

**Type species (by monotypy):** *Sabbathichthys osbournei* n. gen. et n. sp.

**Diagnosis:** A genus of the family Phosichthyidae characterized by the following combination of features: mouth large with an elongate premaxilla occupying most of the dorsal margin of the mouth gape, reaching more than two thirds of the length of the maxilla, bearing about 16 minute needle-like teeth; mandibular joint just behind the posterior edge of the orbit; lower jaw length similar to that of the upper jaw; 42-43 vertebrae (21 caudal); three epurals; dorsal fin with 13 rays; anal fin containing at least 20 rays; pectoral fin with ten rays, extending backwards up to the basipterygia; pelvic fin containing eight rays; seven photophores of the IP series; ten photophores of the PV series; at least four photophores of the VAV series; at least 15 photophores of the AC series; single row of photophores above the anal fin.

**Etymology:** Genus named after the British rock band “Black Sabbath”, and the Greek word “ἰ*χθύς*” meaning fish.

**Remarks:** This genus is referred to the Phosichthyidae based on the presence of a greatly elongate and developed premaxilla, which reaches more than two-thirds of the length of the maxilla; conical jaw teeth, uniform in size; presence of photophores of the IP series; three pectoral-fin radials [67,68].

These features allow us to exclude any attribution to the family Gonostomatidae, whose members are characterized by a premaxilla corresponding to one-third or less of the length of the maxilla, teeth of different sizes forming an alternate pattern of long conical teeth separated by minute needle-like elements, four pectoral-fin radials, and photophores of the IP series absent [69].

*Sabbathichthys osbournei* n. gen. et n. sp. Calzoni, Giusberti & Carnevale

urn:lsid:zoobank.org:act:C22BB739-CC5F-4844–8711-DC74B6858BF4

Figs 10–12

Diagnosis: as for genus.

**Etymology:** Species name dedicated to Ozzy Osbourne (1948-2025), frontman of the British heavy metal band Black Sabbath, who passed away during the preparation of this paper.

**Type locality and horizon:** Marly limestones of the Chiusole Formation (CNE5 and E6-E7a Zones; upper Ypresian), Monte Solane (Sant’Ambrogio di Valpolicella, Verona, Italy).

**Holotype:** MGP-PD 33551, a nearly complete articulated skeleton, 39.1 mm SL.

**Paratype:** IGVR 64077, a nearly complete articulated skeleton.

#### Description.

**Fig 10:**
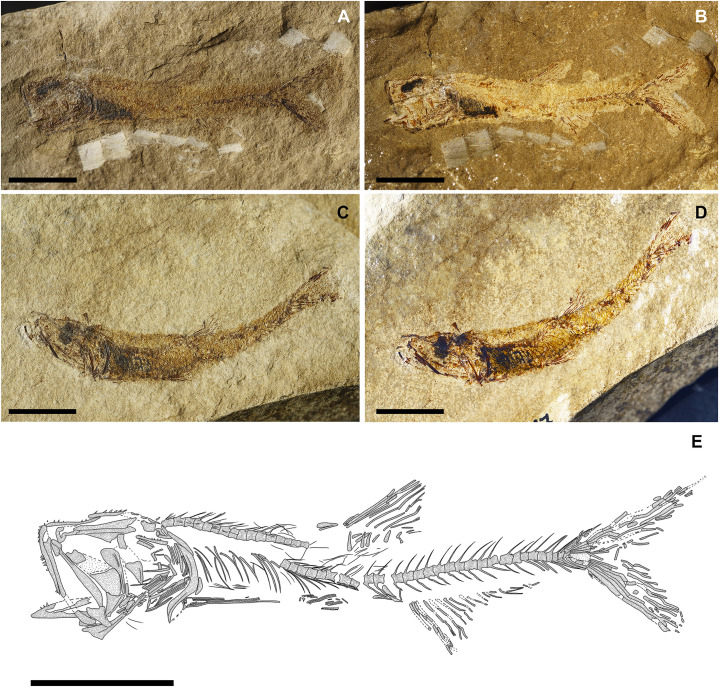
*Sabbathichthys osbournei* n. gen. et n. sp. Lateral view of the holotype, MGP-PD 33551, in natural light **(A)** and coated in alcohol **(B)**. Lateral view of the paratype, IGVR 64077, in natural light **(C)** and coated in alcohol **(D)**. Interpretive reconstruction of the skeleton of the holotype **(E)**. Scale bars 10 mm.

The body is slender and laterally compressed, showing its maximum depth in the head region (BD: 20% of SL; Fig 10; Table 5). The mouth is terminal and extremely large, with the mandibular joint placed slightly posterior to the large orbit (Fig 10E).

**Table 5:** Measurements of *Sabbathichthys osbournei* n. gen. et n. sp.

	*Sabbathichthys osbournei* n. gen. et n. sp.	*Praewoodsia mesogeae*	*Vinciguerria* spp.	*Vinciguerria distincta*	*Vinciguerria obscura*
**SL (mm)**	39.1	60-61	18.7-53	up to 40	33-70
**TL (mm)**	47.2	?	?	?	?
**HL**	29.9	33.3-35.2	23.4-36.1	28.6-29.4	25-30.2
**PD**	53.9	56.7-59	48.9-71.4	54.4-55.7	53.7-61
**PA**	67.2	75-80.3	57.5-78.6	67.7-68.6	70.7-75.6
**PP**	29	?	17.2-31.8	?	?
**PV**	47.7	?	45.5-59.5	44.3-45.6	48.7-55.8
**DFL**	13.3	?	11.1-21.4	?	11.1
**AFL**	21.1	13.3-14.8	11.1-20.3	?	?
**PFL**	17.7	21.3	12-20.4	?	?
**VFL**	?	11.7-14.7	8.4-15.6	?	?
**PRO**	5.7	9.8	5.3-11.6	?	?
**O**	4.7	1.7	6.4-10.7	?	?
**POO**	16.8	?	?	?	?
**DRL**	18.1	?	?	?	?
**AFR**	15.3	?	?	?	?
**BD**	20	20-22.9	14.4-27.2	11.4-14.7	15.9-24.4
**CPL**	14.6	?	?	?	?
**CPH**	10	8.3-9.8	5.9-12.3	5.7-5.9	6-9.8

Includes new data and data from [62,67,70–74]. Values are as a percentage of SL. Abbreviations: AFL: anal-fin base length; AFR: anal-fin ray length; BD: maximum body depth; CPH: caudal peduncle height; CPL: caudal peduncle length; DFL: dorsal-fin base length; DRL: dorsal-fin ray length; HL: head length; O: orbit diameter; PA: preanal distance; PD: predorsal distance; PFL: pectoral-fin length; POO: postorbital distance; PRO: preorbital distance; PP: prepectoral distance; PV: prepelvic distance; SL: standard length; TL: total length; VFL: pelvic-fin length.

**Fig 11:**
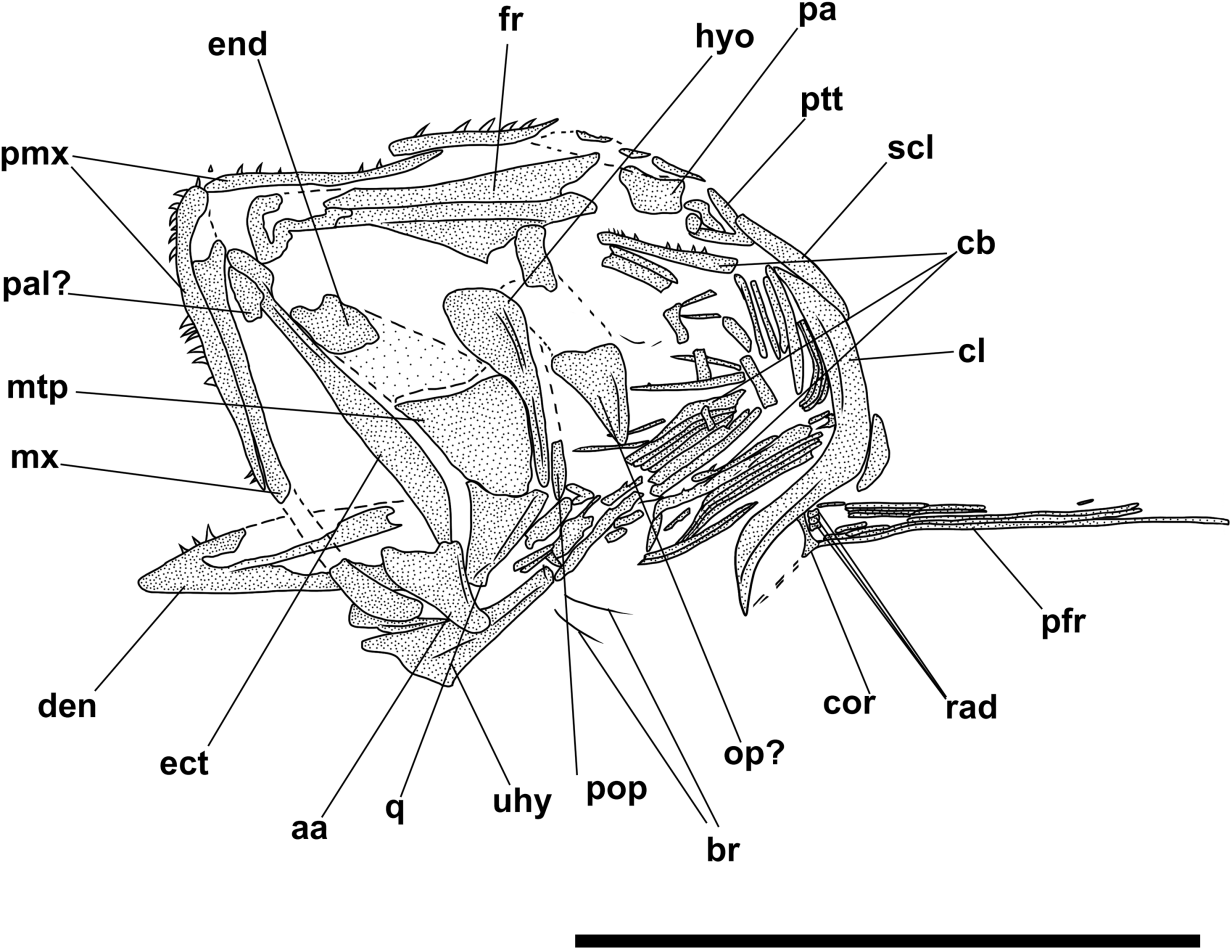
*Sabbathichthys osbournei* n. gen. et n. sp. **Holotype, MGP-PD 33551. Interpretive reconstruction of the cranium.** Scale bar 10 mm. Abbreviations: aa: anguloarticular; br: branchiostegal rays; cb: ceratobranchial; cor: coracoid; cl: cleithrum; den: dentary; ect: ectopterygoid; end: endopterygoid; fr: frontal; hyo: hyomandibula; mtp: metapterygoid; mx: maxilla; op: opercle; pa: parietal; pal: palatine; pfr: pectoral-fin ray; pmx: premaxilla; pop: preopercle; ptt: posttemporal; q: quadrate; rad: pectoral-fin radial; scl: supracleithrum; uhy: urohyal.

The bones of the neurocranium are thin and delicate. The ethmoid region is poorly preserved, with the lateral ethmoid being fragmented. The frontals are elongate, triangular, and narrow, with ridges on their surface that extend back over the posterior edge of the orbit. The parietals are small and polygonal (Fig 11). The other bones of the skull roof are poorly preserved and difficult to describe. The straight and narrow parasphenoid is mainly preserved as an impression only, crossing the orbit in the midline (see IGVR 64077; Fig 10C and 10D). The nasals and the bones of the infraorbital series are not preserved.

The upper jaw bears numerous teeth along the entire upper border of the mouth. The premaxilla is straight, narrow, and elongate, being more than two-thirds (74.7%) of the length of the maxilla; its processes are reduced, and it bears at least 16 minute, needle-like teeth, equally spaced and sized (Fig 11). The maxilla is partially preserved: it is elongate, curved, and slightly expanded posteriorly. The teeth of the maxilla are extremely worn, being similar to those of the premaxilla. The supramaxillae are poorly preserved. The dentary is triangular and slender, bearing small conical teeth. The anguloarticular is triangular.

The suspensorium is inclined forward. The quadrate is triangular with a developed anterior process. The symplectic is small and wedge-shaped. The ectopterygoid is straight and anteriorly inclined. The endopterygoid is large and triangular, while the metapterygoid is almost trapezoid. The hyomandibula is partially preserved and slightly curved anteriorly: it has an elongate and narrow ventral shaft, while its articular head is poorly preserved (see MGP-PD 33551; Figs 10A-B and 11).

The preopercle is poorly preserved, solely represented by its ventral-most portion (MGP-PD 33551; Figs 10A-B and 11). The opercle is rectangular, partially preserved, and displaced from its original position (MGP-PD 33551; Figs 10A-B and 11). The interopercle and subopercle are not preserved.

The hyoid arches are poorly preserved; only the urohyal shows a triangular and anteriorly broad shape, and it is possible to distinguish only a few branchiostegal rays. Of the branchial skeleton, several isolated ceratobranchials are visible, bearing up to eight spinous gill rakers, with smaller indentations on their surface.

The vertebral column consists of 42−43 vertebrae (22−21 + 21; Fig 10E). The centra are rectangular, longer than high, except for the posterior caudal centra that are more compact anteroposteriorly. The neural arches emerge from the anterior half of the centrum. The neural spines are thin and slightly curved, extending posteriorly beyond the end of their respective centrum. The first neural spine fused in the midline is that of the 20^th^ vertebra. The haemal spines are slender and posteriorly curved, similarly to their opposite neural spines. There are long and slender ribs that articulate with the lateral sides of the abdominal vertebrae (except for the first two centra) and reach the ventral edge of the body.

All the abdominal vertebrae bear thin epineurals, which are present up to the 24^th^ or 25^th^ vertebra (see MGP-PD 33551; Fig 10A-B, E). There is no evidence of the epipleurals.

**Fig 12:**
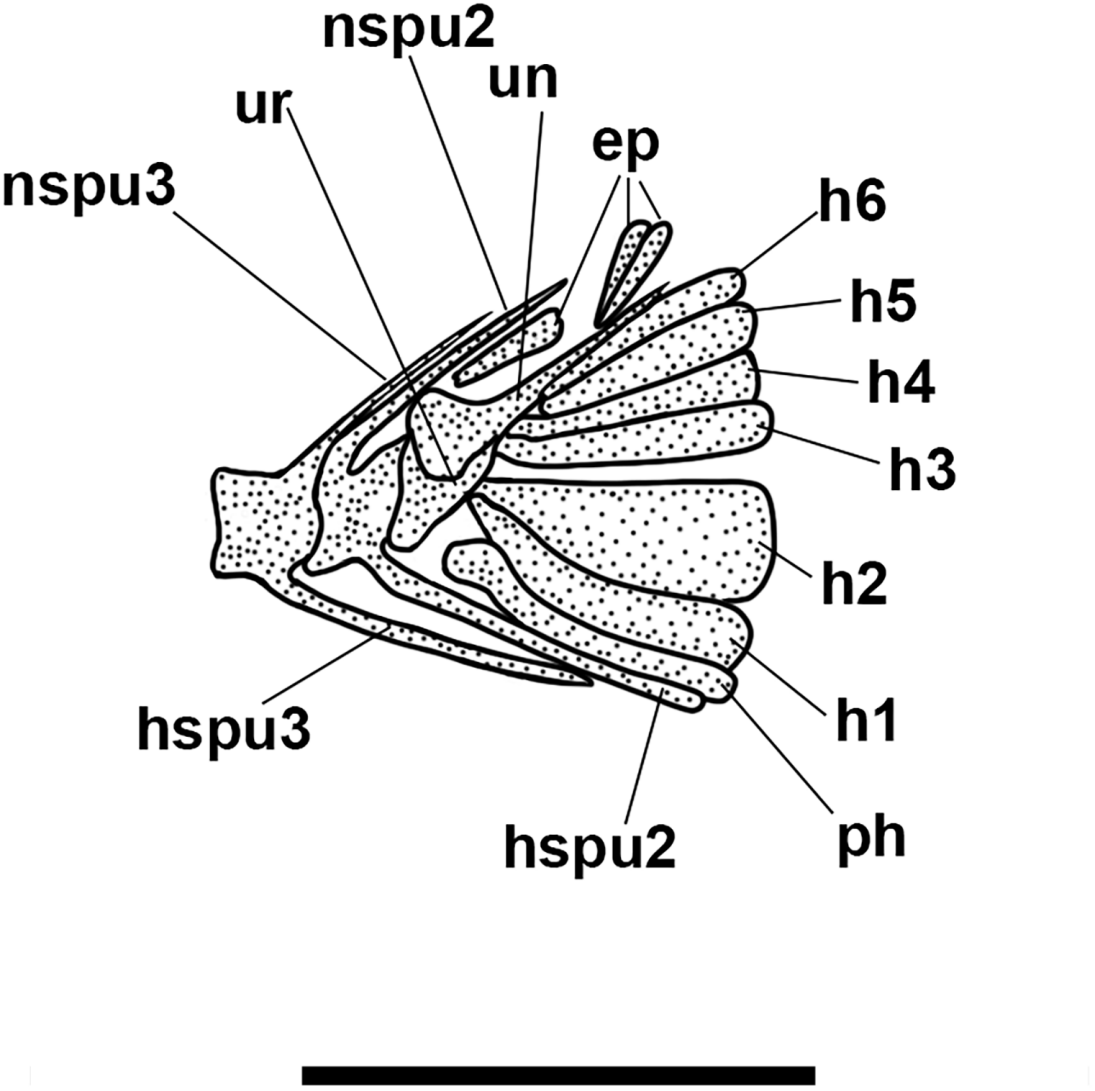
*Sabbathichthys osbournei* n. gen. et n. sp. **Holotype, MGP-PD 33551. Interpretive reconstruction of the caudal skeleton.** Scale bar 5 mm. Abbreviations: ep: epural; h: hypural; hspu2: haemal spine of the second preural vertebra; hspu3: haemal spine of the third preural vertebra; nspu2: neural spine of the second preural vertebra; nspu3: neural spine of the third preural vertebra; ph: parhypural; un: uroneural; ur: urostyle.

The caudal skeleton consists of six autogenous hypurals, an autogenous and slightly expanded parhypural, a single and large uroneural, and three small epurals (Fig 12). The second ural centrum is not preserved. The haemal spines of the second and third preural vertebrae are elongate and fused to the centrum (Fig 12). The caudal fin is forked and bears 19 (10 + 9) principal rays. In addition, there are up to six dorsal and four or five ventral procurrent rays (6, I, 9 + 8, I, 4–5).

Some supraneurals are visible, but their precise number is difficult to establish (see IGVR 64077). The dorsal fin consists of 13 rays, the first two being short and unbranched. The third and fourth rays are the longest of the series. The dorsal fin is supported by thin and rectangular pterygiophores. The first two rays are supported by a large pterygiophore that is inclined forward, almost horizontally. The anal fin inserts behind the level of the posteriormost dorsal fin pterygiophore. The length of the anal-fin base is almost two times that of the dorsal fin (DFL: 13.3% of SL; AFL: 21.1% of SL; Table 5). The anal fin contains at least 20 rays (see MGP-PD 33551; Fig 10A-B, E), the first two being very reduced and unbranched, while the third and fourth rays are the longest of the series. A few anal-fin pterygiophores are preserved, resembling those of the dorsal fin.

The posttemporal is bifurcate and poorly preserved. The supracleithrum is thin and rod-like, strongly inclined anteriorly. The cleithrum is large and crescent-shaped, posteriorly expanded, and with a ventrally pointed and bent-downward process. The coracoid has a developed anterior process, preserved as an impression only. The scapula is difficult to recognize. The pectoral fin inserts ventrally on the body flanks and extends posteriorly up to the pelvic fin; it has ten rays supported by three pectoral-fin radials (see MGP-PD 33551; Fig 11). The pelvic girdle is triangular and narrow. The pelvic fins contain eight rays.

The photophores are preserved as thin black spots of organic matter and are particularly evident in the holotype. Seven photophores are preserved close to the lower jaw, likely belonging to the IP series; ten photophores belong to the PV series, at least four to the VAV series, and at least 15 to the AC series, disposed in a single row above the anal fin. The ORB1 photophore is preserved, and due to inadequate preservation, there is no trace of the ORB2 photophore (possibly some traces are present posteroventral to the orbit of IGVR 64077; Fig 10C and 10D). The squamation consists of small, circular cycloid scales.

#### Discussion.

Recent phylogenetic studies based on molecular and morphological data suggest that the Phosichthyidae are non-monophyletic and include the genera traditionally referred to this family plus *Triplophos* within the expanded Stomiidae [75]. However, since there are still substantial morphological differences between these two families, for the sake of convenience, we still consider the Phosichthyidae as a separate family, following the classification of Nelson et al. [76].

The Paleogene record of the Phosichthyidae includes *Solterichthys macrognathus* from the lower Eocene of Solteri, northern Italy [14], *Vinciguerria distincta* from the Lutetian Dabakhan Formation, Georgia [73,74], *Vinciguerria obscura* [74], and *Praewoodsia mesogeae* from the upper Eocene of Iran [62,67,74,77]. Species of the genera *Ichthyococcus*, *Phosichthys,* and *Vinciguerria* (*V*. *orientalis*) are known from Miocene deposits [67,68].

*Sabbathichthys osbournei* n. gen. et n. sp. can be easily distinguished from the other phosichthyids due to its longer premaxilla, which is more than two-thirds of the length of the maxilla. The premaxilla of the other taxa is much shorter, ranging from extremely reduced (*Ichthyococcus*) to about one-third of the length of the maxilla (*Vinciguerria*; [67,73,74]). The dentition of *S. osbournei* n. gen. et n. sp. consists of minute and uniserial needle-like teeth, unlike those of *Phosichthys* and *Woodsia*, which exhibit a dentition characterized by teeth of two different sizes, and those of *Polymetme* and *Yarrella*, which have biserial teeth on the premaxilla [67].

*S. osbournei* n. gen. et n. sp. differs from *Vinciguerria distincta* by having a deeper body (BD: 20% of SL, CPH: 10% of SL in *S*. *osbournei* n. gen. et n. sp. vs BD: 11.1–14.7% of SL, CPH: 5.7–5.9% of SL in *V*. *distincta*; [74]; Table 5); 42–43 vertebrae (vs 45 in *V. distincta*; [74]; Table 6); anal-fin origin located after the end of the dorsal-fin base (vs located at the mid-point of the dorsal-fin base in *V*. *distincta*; [74]; Table 6); number of photophores of the VAV series (9 vs 10 in *V*. *distincta*; [74]; Table 6) and AC series (15 vs 20–21 in *V*. *distincta*; Table 6; [67,74]).

**Table 6:** Summary of the meristic traits of *Sabbathichthys osbournei* n. gen. et. n. sp. and other extinct photichthyid taxa.

	*Sabbathichthys osbournei* n. gen. et n. sp.	*Praewoodsia mesogeae*	*Solterichthys macrognathus*	*Vinciguerria* spp.	*Vinciguerria distincta*	*Vinciguerria obscura*
**Dorsal-fin rays**	13	12-13	?	12-16	14	12-14
**Anal-fin rays**	20+	12-13	?	12-17	15-20	15-21
**Pectoral-fin rays**	10	10	13	8-12	12	10-12
**Pelvic-fin rays**	8	6-8	?	7-9	8	8-9
**Caudal-fin rays (principal)**	19 (10 + 9)	20 (10 + 10)	?	19 (10 + 9)	19 (10 + 9)	19 (10 + 9)
**Caudal-fin rays (procurrent)**	6 + 4-5	?	?	?	?	?
**Vertebrae (caudals)**	42-43 (21-22 + 21)	39 (21 + 18)	20 + ?	37-44 (19/25 + 16/19)	45 (24 + 21)	40-42 (21/22 + 19-20)
**Branchiostegal rays**	?	?	12	?	?	?
**ORB1**	yes	?	yes	yes	?	yes
**ORB2**	?	?	yes	yes	?	yes
**IP**	7	?	6	7-8	4+	9
**PV**	10	?	9	11-17	?	11-13
**VAV**	9	?	?	7-11	10	8-9
**AC**	15+	?	?	10-17	20-21	17-18

Includes new data and data from [14,62,67,70–74,78–84]. Abbreviations: AC: series of photophores posterior to the anal-fin origin; IP: ventral series of photophores anterior to pectoral-fin base; ORB1: preorbital photophore; ORB2: postorbital photophore; PV: series of photophores between the bases of the pectoral and pelvic fins; VAV: ventral series of photophores between the pelvic-fin base to the anal-fin origin.

*S. osbournei* n. gen. et n. sp. differs from other species of the genus *Vinciguerria* by having a larger premaxilla (more than two thirds of the length of the maxilla vs one third of the length in *Vinciguerria* spp.); three epurals (vs two in *Vinciguerria* spp.), more anal-fin rays (at least 20 vs 12–17 in *Vinciguerria* spp., AFL: 21.1 vs 11.1–20.3% of SL, respectively; [74]; Tables 5 and 6), a more posterior anal-fin origin (just behind the end of the dorsal-fin base vs beneath the middle or latter half of the dorsal fin in *Vinciguerria* spp. [74]), and ten photophores of the PV series (vs 11–17 in *Vinciguerria* spp.; Table 5; [65,68,70,74]).

*S. osbournei* n. gen. et n. sp. differs from *Praewoodsia mesogeae* by having a shorter head (HL: 29.9% vs HL: 33.3–35.2% of SL in *P*. *mesogeae*; [62]; Table 6), 42–43 vertebrae (vs 39 in *P*. *mesogeae*; [62]; Table 5), 19 principal caudal-fin rays (vs 20 in *P*. *mesogeae*; [62]; Table 5), a shorter predorsal and preanal distance (PD: 53.9% and PA: 67.2% of SL vs PD: 56.7–59% and PA: 75–80.3% of SL in *P*. *mesogeae*; [62]; Table 5), at least 20 anal-fin rays (vs 12–13 in *P*. *mesogeae*; [62]; Table 6) and a longer anal-fin base (AFL: 21.1% vs AFL: 13.3–14.8% of SL in *P*. *mesogeae*; [62]; Table 5).

*Sabbathichthys osbournei* n. gen. et n. sp. differs from *Solterichthys macrognathus* by having fewer and smaller teeth on the maxilla, ten pectoral-fin rays (vs 13 in *S*. *macrognathus*; [14]; Table 6) and only one ORB photophore (both ORB1 and ORB2 are present in *S*. *macrognathus*; [14]).

Family Gonostomatidae Gill, 1893 [85]

Genus *Scopeloides* Wettstein, 1886 [86]

**Type species:** *Osmerus glarisianus* Agassiz, 1844 [87]

**Diagnosis:** See Calzoni et al. (2025). [14]

*Scopeloides bellator* Calzoni, Giusberti & Carnevale, 2025 [14]

Fig 13

v2011 Gonostomatidae gen. indet. cf. *Scopeloides*, Zorzin et al., p. 61, fig. 5. [25]

v2014 Gonostomatidae gen. indet. cf. *Scopeloides*, Giusberti et al., p. 6, fig. 4. [15]

+2025 *Scopeloides bellator*, Calzoni et al. 2025, p. 471, fig. 11. [14]

**Referred materials:** IGVR 64072, a nearly complete articulated skeleton; IGVR 64073–67862, a nearly complete articulated skeleton, 58.2 mm SL; IGVR 64074, a nearly complete articulated skeleton, 38.5 mm SL; IGVR 64075–82438, a nearly complete articulated skeleton, 51.2 mm SL; IGVR 64076–82446, a nearly complete articulated skeleton, 48.9 mm SL; IGVR 64096–64097, a nearly complete articulated skeleton, in part and counterpart; IGVR 82406–82432, a nearly complete articulated skeleton, in part and counterpart, 52.3 mm SL; IGVR 82439a, a nearly complete articulated skeleton, 51 mm SL; IGVR 82447, a nearly complete articulated skeleton, 66.8 mm SL; MGP-PD 33454A-B, a nearly complete articulated skeleton, in part and counterpart; MGP-PD 33467, a nearly complete articulated skeleton, 51.8 mm SL; MGP-PD 33500, a nearly complete articulated skeleton; MGP-PD 33526, a nearly complete articulated skeleton, 45.7 mm SL; MGP-PD 33532A-B, a nearly complete articulated skeleton, in part and counterpart, 37.3 mm SL; MGP-PD 33547, a nearly complete articulated skeleton, 55.6 mm SL; MGP-PD 33554, a nearly complete articulated skeleton.

#### Discussion.

**Fig 13:**
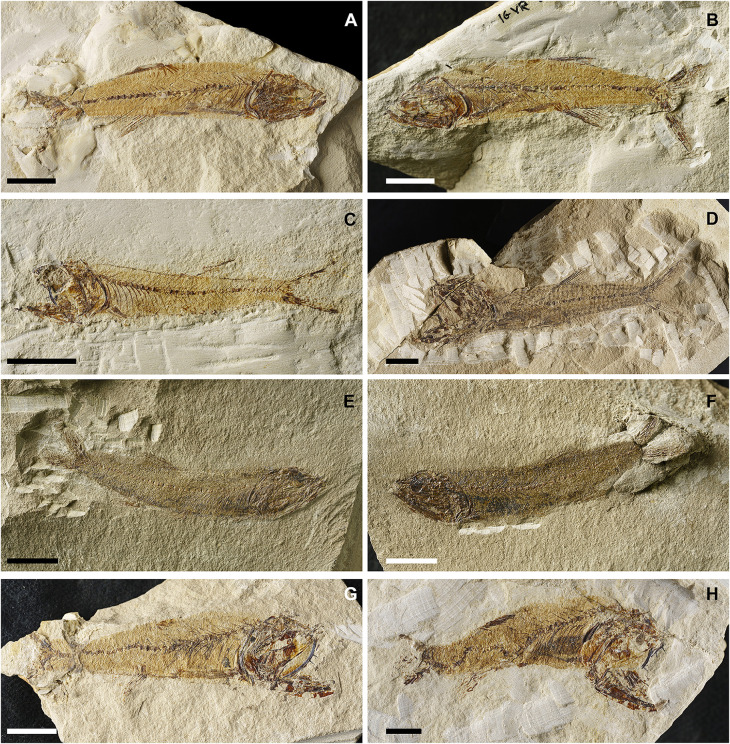
*Scopeloides bellator* Calzoni, Giusberti & Carnevale, 2025 [14]. Lateral view of IGVR 64073-67862 (A-B), IGVR 64074 (C), IGVR 82447 (D), IGVR 64075-82438 (E-F), MGP-PD 33457 (G), MGP-PD 33500 (H). Scale bars 10 mm.

The specimens (Fig 13) are referred to *S*. *bellator* since they exhibit a rather robust and thick body (BD: 19.7–26% SL; CPH: 10.2–12.6% SL; Table 7), two or three premaxillary fang-like teeth and more than seven maxillary fang-like teeth, anterior supramaxilla large and ovoid, posterior suparamaxilla small and pointed, dentary with more than six fang-like teeth, urohyal with straight dorsal and slightly curved ventral margin, less than 39 vertebrae, epineurals and epipleurals attached close to the base of the neural and haemal spines of the first four or five caudal vertebrae, dorsal-fin origin anterior to the anal fin (PD: 51.3–59% of SL; PA: 57.9–66.8% of SL; Table 7), and other meristic and morphometric features (e.g., median and paired fins ray count, head length, predorsal, preanal, prepectoral and prepelvic distances and anal-fin base length, see Tables 7 and 8).

**Table 7:** Measurements of the different species of the genus *Scopeloides.*

	*Scopeloides bellator* (type series)	*Scopeloides violator* (type series)	*Scopeloides* cf. *glarisianus*
**SL (mm)**	37.3-66.8 (37.1-62.8)	27.8-56.1 (45.8-83.4)	13.7-40.6
**TL (mm)**	46.4-67 (43.2-79.1)	34.7-60.2 (48.3-94.9)	14.3-55.2
**HL**	27.8-31.4 (27.1-30.9)	23.8-31.4 (20.8-27)	21.3-30.3
**PD**	51.3-59 (55.7-57.5)	52.9-61.2 (52.2-60)	49-53.6
**PA**	57.9-66.8 (53.6-67.1)	66.5-68.7 (59-68.3)	55.2-59.7
**PP**	26.8-31.9 (22.1-34.3)	30.4-30.5 (26.5-32.7)	21.4-37.9
**PV**	46.8-52.6 (43.1-52.5)	47.2-52.5 (46.1-57.1)	50.5-52.6
**DFL**	10.7-15.5 (9.4-15.6)	12.8 (12.2-14.7)	8.7-11.9
**AFL**	22.7-30.2 (21.6-31.8)	? (21.5-28.7)	33
**PFL**	14.9-20.8 (15.7-19.5)	9.3 (12.1-21.3)	9-15.2
**VFL**	7.7-12.3 (9.2-12.4)	10.1-11.5 (8-12.7)	7.9-9.1
**PRO**	6.2-10.8 (5.8-10.1)	5.9-6.8 (4.2-7.7)	9.3-13.4
**O**	4.1-8.9 (5.6-9.9)	2.6? (4-7.9)	5.2-5.4
**POO**	11.2-16.6 (8.8-18.8)	10.4-19 (11.3-19.4)	11-12.3
**DRL**	11.9-21.6 (11.4-20.1)	13.7-13.8 (9.3-20.9)	15.2-20
**AFR**	12.3-18.1 (9.9-22.8)	11.6 (12.1-20.5)	?
**BD**	19.7-26 (20.4-31.4)	11.7-15.5 (14.1-18.9)	17.1-20.6
**CPL**	6.5-11.1 (6.8-12.1)	8.4 (5.7-9.4)	11.7
**CPH**	10.2-12.6 (9.6-17.3)	5.8-7.2 (6.5-9.1)	8.4-10.2

Includes new data and data from [14]. Values in brackets for *S*. *violator* and *S*. *bellator* refer to the specimen belonging to the type series [14]. Values are as a percentage of SL. Abbreviations: AFL: anal-fin base length; AFR: anal-fin ray length; BD: maximum body depth; CPH: caudal peduncle height; CPL: caudal peduncle length; DFL: dorsal-fin base length; DRL: dorsal-fin ray length; HL: head length; O: orbit diameter; PA: preanal distance; PD: predorsal distance; PFL: pectoral-fin length; POO: postorbital distance; PRO: preorbital distance; PP: prepectoral distance; PV: prepelvic distance; SL: standard length; TL: total length; VFL: pelvic-fin length.

**Table 8:** Summary of the meristic traits of the different species of the genus *Scopeloides.*

	*Scopeloides bellator*	*Scopeloides violator*	*Scopeloides* cf. *glarisianus*	*Scopeloides glarisianus*
**Dorsal-fin rays**	13-15	14-15	13-14	13-15
**Anal-fin rays**	24-29	27-30	24-27	26-29
**Pectoral-fin rays**	9-14	10-15	10-13	11-12
**Pelvic-fin rays**	7-9	7-9	7-8	7-9
**Caudal-fin rays (principal)**	19 (10 + 9)	19 (10 + 9)	19 (10 + 9)	19 (10 + 9)
**Caudal-fin rays (procurrent)**	6-9 + 6-9	8-11 + 7-9	6-7 + 5-6	6-7 + 6-7
**Vertebrae (caudals)**	35-39 (18-20)	40-43 (20-21)	37 (19-20)	39-41 (21-22)
**Branchiostegal rays**	10-12	12-14	10	9
**Fangs (pmx)**	2-3	2	2-3	2-4
**Fangs (mx)**	6-8	6-7	9-12	9-12
**Fangs (den)**	7-8	5-6	6-7	6
**PV**	6-11	6-10	6	9-10
**VAV**	6-8	5	?	?
**AC**	18	17-20	18	20
**Epineurals attached to haemal spines**	5	5	8	7-8
**Epipleurals attached to haemal spines**	4-5	6	8	6-7

Includes new data and data from [14,67,88,89]. Abbreviations: AC: series of photophores posterior to the anal-fin origin; den: dentary; mx: maxilla; pmx: premaxilla; PV: series of photophores between the bases of the pectoral and pelvic fins; VAV: ventral series of photophores between the pelvic-fin base to the anal-fin origin.

**Fig 14:**
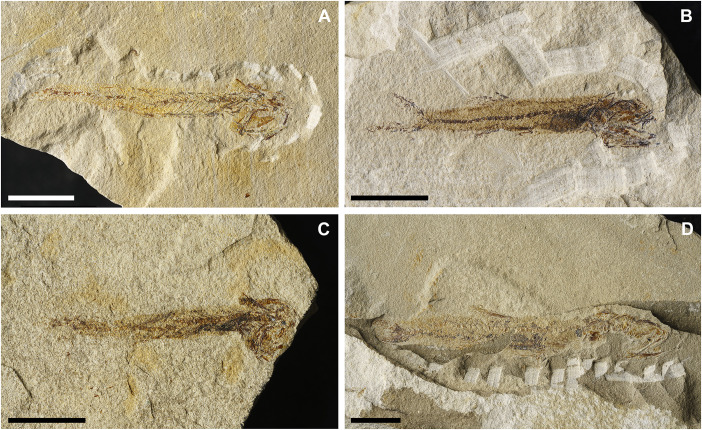
*Scopeloides violator* Calzoni, Giusberti & Carnevale, 2025 [14]. Lateral view of MGP-PD 33470 (A), MGP-PD 33479 (B); MGP-PD 33504 (C), MGP-PD 33510 (D). Scale bars 10 mm.

**Occurrence**: upper Ypresian of Solteri (Trento) and Monte Solane (Verona) sites, northeastern Italy. *Scopeloides violator* Calzoni, Giusberti & Carnevale, 2025 [14] Fig 14 +2025 *Scopeloides violator*, Calzoni et al., p. 466, fig. 7. [14]

**Referred materials** MGP-PD 33469, a nearly complete articulated skeleton; MGP-PD 33470, a nearly complete articulated skeleton, 37.8 mm SL; MGP-PD 33479, a nearly complete articulated skeleton, 29.8 mm SL; MGP-PD 33504, a nearly complete articulated skeleton, 27.8 mm SL; MGP-PD 33510, a nearly complete articulated skeleton, 56.1 mm SL.

#### Discussion.

Five specimens are referred to *S*. *violator* since they exhibit the characteristic traits of this species (Fig 14; see [14]), including, a notably slender and thin body (BD: 11.7–15.5% SL; CPH: 5.8–7.2% SL; Table 7), two premaxillary fang-like teeth and less than eight maxillary fang-like teeth, anterior supramaxilla large and ovoid, posterior supramaxilla smaller and with an anterodorsally pointed process, dentary with five or six fang-like teeth, urohyal triangular with slightly curved ventral edge and straight dorsal edge, more than 39 vertebrae, five or six epineurals and five or six epipleurals inserting close to the base of the neural and haemal spines of the caudal vertebrae, dorsal-fin origin anterior to the anal fin, and certain meristic and morphometric features (e.g., anal, pectoral and pelvic fins ray count, head length, predorsal, preanal prepectoral and prepelvic distance, see Tables 7 and 8).

**Occurrence**: upper Ypresian of Solteri (Trento) and Monte Solane (Verona) sites, northeastern Italy.

*Scopeloides* cf. *glarisianus* (Agassiz, 1844) [87]

Fig 15

cf.

+1844 *Osmerus glarisianus* Agassiz, Agassiz, p. 109, pl. 12, figs. 3-4. [87]

1886 *Scopeloides glaronensis* (Ag.), Wettstein, p. 55–57, pl. 2. figs. 7-13. [86]

1931 *Mrazecia mrareci* Pauca, Pauca, p. 148, 151. [90]

1933 *Mrazecia mrazeci* Pauca, Pauca, p. 40–42, figs. 10-11, pl. 2, figs. 4-5, pl. 3, fig. 6. [91]

1934 *Scopeloides mrareci* (Pauca), Pauca, p. 90–91. [92]

1938-40 *Scopeloides mrazeci* (Pauca), Kalabis, p. 28–33. [93]

1948 *Scopeloides glarisianus* (Ag.), Kalabis, p. 136–139, pl. I, fig. 1. [94]

1960 *Scopeloides glarisianus* (Ag.), Danilchenko, p. 27–28, pl. 2, fig. 1. [95]

**Fig 15:**
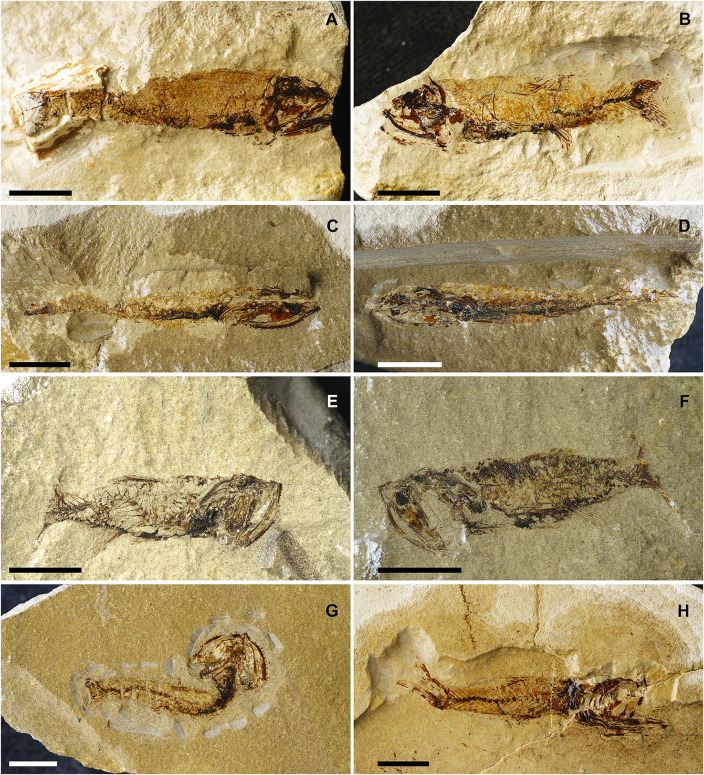
*Scopeloides* cf. *glarisianus.* Lateral view of IGVR 82433-82434 **(A-B)**, MGP-PD 33451A-B **(C-D)**, MGP-PD 33463A-B **(E-F)**, MGP-PD 33550 **(H)**, and dorsal view of MGP-PD 33503 **(G)**, all coated in alcohol. Scale bars 10 mm.

1967 *Scopeloides glarisianus* (Ag.), Arambourg, p. 43, pl. 2, figs. 2-7, 9; Text-figs. 14, 15, 17. [62]

1968 *Scopeloides glarisianus* (Ag.), Jerzmanska, p. 27–28, pl. 2, fig. 1. [96]

1977 *Scopeloides glarisianus* (Ag.), Ciobanu, p. 67, pl. 16, fig. 1. [97]

1977 *Scopeloides paucai* Ciobanu, p. 68–69, pl. 17, fig. 1. [97]

1989 *Scopeloides glarisianus* (Ag.), Gregorová, p. 89–90, pl. IV, fig. 7. [98]

1989 *Scopeloides* sp. Gregorová, p. 89–90, pl. 1, fig. 1. [98]

1997 *Scopeloides glarisianus* (Agassiz), Gregorová, p. 133–134, pl. I-II. [88]

2005 *Scopeloides glarisianus* (Agassiz, 1833–1844), Prokofiev, p.S100, fig. 5a-b. [67]

2012 *Scopeloides glarisianus* (Agassiz), Přikryl et al., p. 379–383, figs. 1A, 2A-B, 3A-B, 4, 6–8-8. [99]

**Referred materials** IGVR 64086–64087, a nearly complete articulated skeleton, in part and counterpart, 29 mm SL; IGVR 82411, a nearly complete articulated skeleton, 13.7 mm SL; IGVR 82433–82434, a nearly complete articulated skeleton, in part and counterpart; MGP-PD 33451A-B, a nearly complete articulated skeleton, in part and counterpart; MGP-PD 33463A-B, a nearly complete articulated skeleton, in part and counterpart; MGP-PD 33503, a nearly complete articulated skeleton and MGP-PD 33550, a nearly complete skeleton, 40.6 mm SL.

#### Description.

Small to medium-sized *Scopeloides* species characterized by a slender body (BD: 17.1–20.6% of SL), with a large terminal mouth and a small orbit (Fig 15).

The frontals are the largest bones of the skull roof; these are triangular with longitudinal striae on their surface. The other bones of the skull roof are fragile and poorly preserved.

The premaxilla is short (around one fourth of the length of the maxilla) and bears two or three long conical teeth separated by several smaller needle-like teeth. The maxilla is sigmoid and bears 9–12 long and thin caniniform teeth separated by several smaller, needle-like teeth. The anterior supramaxilla is poorly preserved, while the posterior supramaxilla has a rounded base and a pointed anterodorsal end (see MGP-PD 33463B, MGP-PD 33550; Fig 15F and 15H). The dentary is triangular and bears six or seven caniniform teeth separated by smaller teeth. The anguloarticular is triangular.

The hyomandibula is anteriorly inclined, with a narrow ventral shaft and a rhomboid head.

The hyoid arch has a narrow anterior ceratohyal and up to ten branchiostegal rays. The urohyal is poorly preserved.

The vertebral column comprises up to 37 vertebrae (19–20 caudals). Thin and curved epineurals attach to the base of the neural arches of the abdominal vertebrae and of the anterior eighth caudal vertebrae. Epipleurals insert at the midlength of the haemal arches in the anterior eight caudal vertebrae.

The caudal skeleton comprises six autogenous hypurals, an autogenous parhypural, two uroneurals, and three epurals. The caudal fin is forked with 19 (10 + 9) principal rays plus six or seven dorsal and five or six ventral procurrent rays.

The dorsal fin contains 13 or 14 rays. The anal-fin origin is slightly posterior to the dorsal fin and has a remarkably larger base; it has 24–27 rays, the anterior being much elongated into a lobe. The pterygiophores of the dorsal and anal fins are very similar.

The pectoral fin contains 10–13 rays, while the pelvic fins have seven or eight rays.

Just six photophores of the PV series and 18 of the AC series are noticeable in the available material.

#### Discussion.

The specimens documented herein cannot be attributed to the other two species of the genus *Scopeloides* reported herein (*S*. *violator* and *S*. *bellator*) due to their peculiar dentition (two or three caniniform teeth in the premaxilla and nine to 12 caniniform teeth in the maxilla [14]. Both *S*. *violator* and *S*. *bellator* never exhibit more than eight fang-like maxillary teeth ( [14]; Table 8). The anterior supramaxilla is not well preserved, but it does not seem to be as large as that of *S*. *violator* and *S*. *bellator*, which can reach half the length of the maxilla. In addition, in our material, epineurals and epipleurals are present up to the eighth caudal vertebra (vs up to the fifth caudal vertebra in the other two species; [14]). Regarding the number of vertebrae, the specimens described herein cannot be assigned to *S. violator* as they possess fewer than 40 vertebrae ( [14]; Table 8). Finally, their slim outline (BD: 17.1–20.5% of SL) is consistent with that of *S*. *glarisianus* (BD: 15.6–20% of SL; [67]) and differs from the thinner body outline of *S*. *violator* and the thicker body outline of *S*. *bellator* ( [14]; Figs 13-15; Table 7).

These specimens are therefore tentatively referred to *Scopeloides* cf. *glarisianus* since their vertebral count does not fit with that of the type species Oligocene material of *S. glarisianus* (37 vs 39–41 vertebrae, respectively; see [88,89]).

*Scopeloides* sp.

**Referred materials:** 31 partially articulated skeletons are referred to the genus *Scopeloides*, ranging from 43.8 to 56.3 mm SL.

#### Description.

These specimens show some of the typical features of the genus *Scopeloides* (e.g., sculptured frontals, long fang-like teeth, two supramaxillae, epineurals and epipleurals attached close to the base of the spines; [67]) but the inadequate preservation of other useful traits prevents their attribution at the species level.

Order Myctophiformes *sensu* Johnson, 1982 [100]

Family Myctophidae Gill, 1893 [85]

Subfamily Eomyctophinae Prokofiev, 2006 [101]

Genus *Eomyctophum* Daniltschenko, 1947 [102]

**Type species** *Eomyctophum koraense*, Daniltshenko, 1947 [102]

*Eomyctophum mainardii* Calzoni, Giusberti & Carnevale, 2025 [14]

Fig 16

v2011 Myctophidae gen. indet. cf. *Eomyctophum*, Zorzin et al., p. 61, fig. 6. [25]

v2014 Myctophidae gen. indet. cf. *Eomyctophum*, Giusberti et al., p.6, fig. 4C. [15]

+2025 *Eomyctophum mainardii*, Calzoni et al. p. 477, fig. 13. [14]

**Emended diagnosis:** Moderate-sized *Eomyctophum* species characterized by the following combination of traits: mouth large with mandibular joint one orbit diameter from the posterior margin of the orbit; a single small supramaxilla; 32–34 vertebrae, (16–17 caudals); forked caudal fin with 19 principal rays plus six-seven dorsal and five-eight ventral procurrent rays; dorsal fin containing 14–16 rays; anal fin with 14–16 rays; pectoral fin comprising 12–14 rays; large pelvic fins with 7–9 rays; cleithrum with well-developed posterior lamina and moderately developed posteromedial shelf; pectoral fins short and narrow, smaller than the pelvic fins; basipterygium extending from the level of the posterior margin of the pectoral girdle to in front of the dorsal-fin insertion, its length corresponds to the length of four-five vertebrae; moderately short prepelvic distance (PV: 43.6–49.7% of SL); pelvic fins elongate and larger than the pectoral fins; distance between pectoral and pelvic fins shorter than the distance between pelvic- and anal-fin insertions.

**Referred materials:** IGVR 64078–67861, nearly complete articulated skeleton, in part and counterpart, 62.8 mm SL; IGVR 64079–64112, a nearly complete articulated skeleton, in part and counterpart, 44.6 mm SL; IGVR 64099–64100, a nearly complete articulated skeleton; IGVR 67857–67862, a nearly complete articulated skeleton; IGVR 67863, a nearly complete articulated skeleton; MGP-PD 33495, a nearly complete articulated skeleton, measuring 37.5 mm SL; MGP-PD 33447a, b, two nearly complete articulated skeletons on the same slab; MGP-PD 33476, a nearly complete articulated skeleton; MGP-PD 33490, a nearly complete articulated skeleton; MGP-PD 33514, a nearly complete articulated skeleton; MGP-PD 33529, an incomplete articulated skeleton, lacking the posterior portion of the body; MGP-PD 33553, a nearly complete articulated skeleton.

#### Discussion.

**Fig 16:**
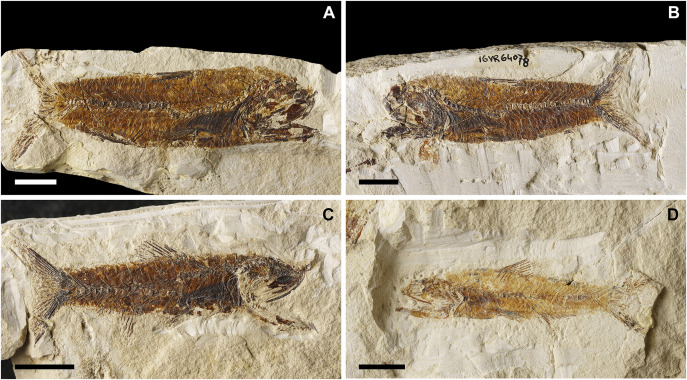
*Eomyctophum mainardii* Calzoni, Giusberti & Carnevale, 2025 [14]. Lateral view of IGVR 64078-67861 (A-B), IGVR 67863 (C), IGVR 67856b (D). Scale bars 10 mm.

The specimens (Fig 16) are referred to *E*. *mainardii* by having a robust body (BD: 24.1–27.4% of SL; CPH: 12.6–17% of SL; Table 9), pelvic girdle almost contacting the pectoral girdle, short prepelvic distance (PV: 45.7–49.7% of SL; Table 9), and distance between pectoral and pelvic fins significantly shorter than that between the pelvic and anal fins (PVD: 12.7–15.6% of SL; VAD: 19.8–25% of SL; Table 9). Moreover, a typical feature of *E*. *mainardii* is the pelvic-fin length that almost doubles the pectoral-fin length (VFL: 16.3–19.7% of SL; PFL: 9.3–10.3% of SL; [14]; Table 9). On the other hand, in the type species *E*. *koraense,* the pectoral fins are longer than the pelvic fins [101]. Aside from other meristic and morphometric differences, another diagnostic trait is the basipterygium length, which in our specimens is more comparable to that of *E*. *mainardii* rather than that of *E*. *koraense* [14,101]. Our material also differs from the other species of the genus for the vertebral count (32–34 vertebrae vs less than 30 in *E*. *polysarcus* and 34–36 in *E*. *cozlae*; [103,104]), from which it differs also for other meristic traits (e.g., dorsal-fin rays, pelvic-fin rays; [97,101,103,104]; Table 10).

**Table 10:** Summary of the meristic traits of the different species of the genus *Eomyctophum* and *Oligophus moravicus.*

	*Eomyctophum mainardii*	*Eomyctophum cozlae*	*Eomyctophum koraense*	*Eomyctophum polysarcus*	*Oligophus moravicus*
**Dorsal-fin rays**	12-16	12	12-14	10-14	12-14
**Anal-fin rays**	14-16	16	14-17	?	14-17
**Pectoral-fin rays**	10-15	?	12-15	?	10
**Pelvic-fin rays**	7-9	?	8	8-10	8
**Caudal-fin rays (principal)**	19 (10 + 9)	12?	19 (10 + 9)	?	19 (10 + 9)
**Caudal-fin rays (procurrent)**	6-11 + 6-9	10?	8-9 + 7-8	?	9 + 8
**Vertebrae**	32-34	34-36	32-34	30	35-37
**Supramaxilla**	yes	yes	yes	?	no
**Second ural centrum**	yes	yes	yes	?	no
**Size**	>70 mm SL	<60mm SL	<60 mm SL	?	<60 mm SL

Includes new data and data from [14,97,103,104].

**Table 9:** Measurements of *Eomyctophum mainardii* from Monte Solane.

	*Eomyctophum mainardii* (holotype)
**SL (mm)**	25.2-75.7 (68.1)
**TL (mm)**	31.7-89.1 (70)
**HL**	29.3-30.8 (28.4)
**PD**	40.7-46.3 (48.6)
**PA**	64.3-72 (66.7)
**PP**	31.6-37.9 (32.2)
**PV**	45.7-49.7 (43.6)
**DFL**	16.9-20.9 (18.8)
**AFL**	15.9-17.2 (14.4)
**PFL**	9.3-10.3 (8.5)
**VFL**	16.3-19.7 (20.6)
**PVD**	12.7-15.6 (10.7)
**VAD**	19.8-25 (26.2)
**PRO**	5.1-7.8 (6.2)
**O**	6.1-8.9 (6.7)
**POO**	14.7-19.3 (15.5)
**DRL**	12.7-22.1 (21.2)
**AFR**	10-18.1 (13.1)
**BD**	24.1-27.4 (26.9)
**CPL**	12.5-17.8 (10.1)
**CPH**	12.6-17 (16.3)

Values in brackets refer to the measurements of the holotype of the species from Solteri (see [14]). Includes new data and data from [14]. Values are as a percentage of SL. Abbreviations: AFL: anal-fin base length; AFR; anal-fin ray length; BD: maximum body depth; CPH: caudal peduncle height; CPL: caudal peduncle length; DFL: dorsal-fin base length; DRL: dorsal-fin ray length; HL: head length; O: orbit diameter; PA: preanal distance; PD: predorsal distance; PFL: pectoral-fin length; POO: postorbital distance; PRO: preorbital distance; PP: prepectoral distance; PV: prepelvic distance; PVD: distance between pectoral and pelvic fins; SL: standard length; TL: total length; VAD: distance between pelvic and anal fins; VFL: pelvic-fin length.

**Occurrence**: upper Ypresian of Solteri (Trento) and Monte Solane (Verona) sites, northeastern Italy.

Division Percomorphacea Wiley & Johnson, 2010 [105]

Order Scombriformes (= clade Pelagia *sensu* Miya et al., 2013) [106]

Suborder Trichiuroidea *sensu* Nakamura & Parin, 1993 [107]

Family Euzaphlegidae Daniltshenko, 1960 [95]

*Acanthophleges* n. gen. Calzoni, Giusberti & Carnevale

urn:lsid:zoobank.org:act:2B5A8AF3-40D8-43E8-9E6A-D2BACB42B9E5

**Type species (by monotypy):** *Acanthophleges lessiniae* n. gen. et. n. sp.

**Diagnosis:** A genus of the family Euzaphlegidae showing the following combination of features: lower jaw slightly prognathous; lower jaw articulation below the posterior-most portion of the orbit; opercle with moderately deep posterior notch; ribs elongate and slender; first dorsal fin with at least eight spines; second dorsal fin with at least 14 rays; anal fin with two spines and 22 rays; pectoral fins inserting low on body and containing 16 short rays; pectoral-fin length shorter than head length; pelvic fins thoracic with one spine plus five rays; small scales bearing thin spines.

**Etymology:** Named after the Greek word *“*ἄ*κανθα”* meaning “spine”, in reference to its peculiar squamation, and the ending of the family name Euzaphlegidae.

**Remarks and comparison:** *Acanthophleges* n. gen. is tentatively referred to the Euzaphlegidae due to its elongate and semi-fusiform body; mouth large; lower jaw slightly prognathous; jaw teeth uniserial; fangs absent in both upper and lower jaws; first dorsal fin with eight spines; second dorsal fin consisting of at least 14 rays; anal fin with two spines and 22 rays; finlets absent; pelvic fins thoracic, consisting of a spine and 5 soft rays; caudal fin forked [95,108].

The moderately fusiform and relative deep body (vs extremely thin and compressed in Trichiuridae), absence of fangs in both upper and lower jaws, lack of supramaxilla, 29 vertebrae (vs at least 32 in Gempylidae and at least 50 in Trichiuridae), forked and developed caudal fin (vs often reduced or absent in Trichiuridae), dorsal fins separated by a gap (vs running across all the body in Trichiuridae), and pelvic fins not reduced, with one spine and five rays (vs usually reduced or absent in Trichiuridae; [107,109,110]) allow to exclude any attribution of *Acanthophleges* n. gen. to the other trichuiroid families Gempylidae and Trichiuridae.

**Fig 17:**
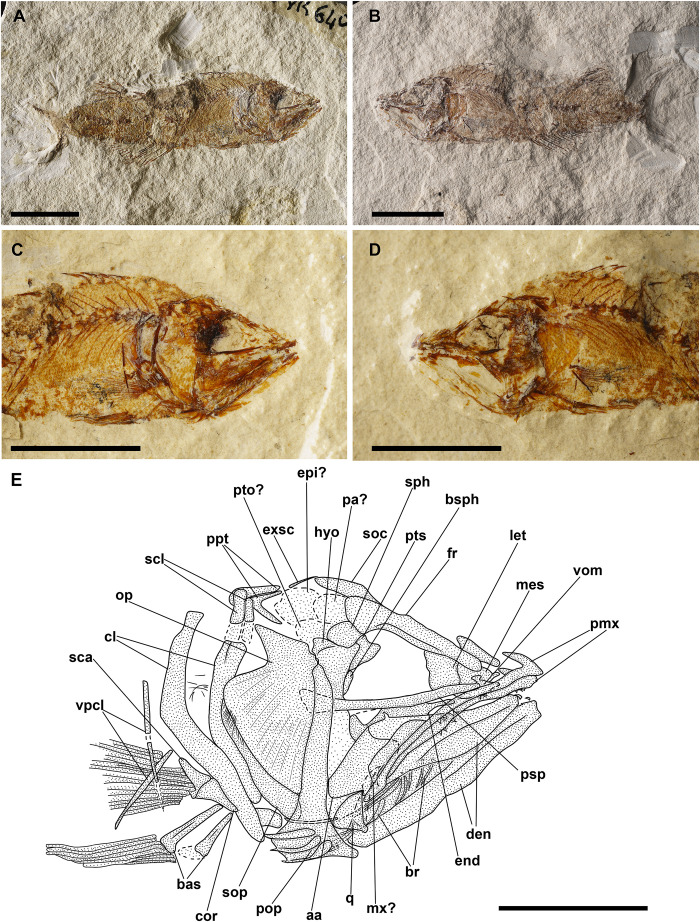
*Acanthophleges lessiniae* n. gen. et n. sp. Lateral view of the holotype, IGVR 64085-67877, in normal light **(A-B)** and detail of the cranium coated in alcohol **(C-D)**. Interpretive reconstruction of the cranium **(E)**. Scale bars 10 mm **(A-D)**; 5 mm **(E)**. Abbreviations: aa: anguloarticular; bas: basipterygium; br: branchiostegal rays; bsph: basisphenoid; cl: cleithrum; cor: coracoid; den: dentary; end: endopterygoid; epi: epioccipital; exsc: extrascapular; fr: frontal; hyo: hyomandibula; let: lateral ethmoid; mes: mesethmoid; mx: maxilla; op: opercle; pa: parietal; pmx: premaxilla; pop: preopercle; psp: parasphenoid; pto: pterotic; pts: pterosphenoid; ptt: posttemporal; q: quadrate; sca: scapula; scl: supracleithrum; soc: supraoccipital; sop: subopercle; sph: sphenotic; vom: vomer; vpcl: ventral postcleithrum.

*Acanthophleges lessiniae* n. gen. et n. sp. Calzoni, Giusberti & Carnevale

urn:lsid:zoobank.org:act:AA460BEE-4B88-4E2E-9DD1-C103E052708E Fig 17

Diagnosis: As for genus.

**Etymology:** Species named after the “Lessini Mountains”, where the Monte Solane site is located.

**Type locality and horizon:** Marly limestones of the Chiusole Formation (CNE5 and E6-E7a Zones; upper Ypresian), Monte Solane (Sant’Ambrogio di Valpolicella, Verona, Italy).

**Holotype (by monotypy):** IGVR 64085–67877, a partially complete articulated skeleton, lacking the posterior portion of the body, including the caudal skeleton, in part and counterpart.

#### Description.

The specimen measures under 50 mm from the tip of the premaxilla to the last preserved vertebra. The body is rather slender, with the maximum body depth at the level of the pelvic girdle and gradually tapering towards the caudal peduncle.

The head is less than a third of the total length of the preserved portion of the body. The mouth is wide and terminal. The orbit is large (Fig 17).

The neurocranium is triangular with a developed ethmoid region. The mesethmoid and the lateral ethmoid are robust and almost quadrangular in outline. The vomer is poorly preserved (see IGVR 64085; Fig 17C and 17E). The frontals are tapered anteriorly and follow the profile of the orbit, becoming thickened posteriorly. Most of the other bones of the skull roof are poorly preserved, although it is possible to determine their shape (Fig 17E). The parietal is quadrangular. The sphenotic is almost triangular. The pterotic is rectangular. The epioccipital and the other bones of the occipital and otic regions are poorly preserved. The supraoccipital crest is moderately developed and triangular. The parasphenoid is straight and narrow. The pterosphenoid and basisphenoid are partially exposed. The nasals and the infraorbital bones are difficult to recognize.

The premaxilla is curved, with a short and pointed ascending process; a few small, conical teeth are preserved. The maxilla is poorly preserved (Fig 17C). There is no trace of a supramaxilla. The dentary is triangular and rather deep; it bears small conical teeth, slightly retrorse, similar to those of the upper jaw but slightly larger in size. The anguloarticular is trapezoid in outline.

The quadrate is almost triangular with a developed anterior process. The symplectic is not preserved. Only the posterior portion of the ectopterygoid is visible, being curved and narrow (see IGVR 67877; Fig 17D and 17E). The endo- and metapterygoid are not properly exposed and are difficult to describe. The palatine is not preserved. The ventral shaft of the hyomandibula is not exposed, and only its broad head and opercular process are exposed.

The preopercle is L-shaped, with a broad ventral portion bearing three thick spines and a thin vertical arm. The opercle is large and trapezoid, with a moderately deep posterodorsal notch. The subopercle is arcuate, while the interopercle is not preserved.

The hyoid arc is partially preserved, although the shape of the anterior and posterior ceratohyals cannot be determined. There are seven slender and saber-like branchiostegal rays. Of the branchial skeleton, some fragments of ceratobranchials with numerous gill rakers are preserved.

The vertebral column is incomplete: at least 29 vertebrae characterized by almost rectangular centra are preserved. The neural prezygapophyses are rather developed. The neural spines are thin and slightly curved. Their opposite haemal spines are almost identical in outline. There are extremely elongate and curved ribs that do not reach the ventral margin of the body, in large part articulated with the lateral side of all abdominal centra. There are no preserved epineurals.

The caudal fin is poorly preserved, and only the forked shape of the fin is noticeable.

Two delicate supraneurals are preserved (Fig 17C and 17D). There are two separate dorsal fins, albeit poorly preserved. The first dorsal fin consists of at least eight spines supported by wedge-like pterygiophores; the first spine is supernumerary on the first pterygiophore. The second dorsal fin is incomplete and shows at least 14 distally branched rays supported by thin pterygiophores; the number of spines is not possible to establish due to poor preservation of the anterior part of this fin. The anal fin is rather large and contains two spines followed by 22 rays, the first of which being the longest of the series. The anal-fin pterygiophores are thin and straight.

The posttemporal is V-shaped and articulates with an extremely thin and styliform element that could be interpreted as an extrascapular (Fig 17E). The supracleithrum is partially preserved and rod-like. The cleithrum is large and curved with a tapered ventral end. The coracoid is narrow and anteriorly pointed. The scapula is almost triangular in outline. The ventral postcleithrum is remarkably elongate, almost reaching the ventral margin of the body. The dorsal postcleithrum is not preserved. The pectoral fin inserts low on the body flanks and contains 16 rays. The pelvic girdle is thoracic. The basipterygium is narrow, and the pelvic fins contain one spine plus five rays each.

The whole body is covered by numerous minute scales with a small upright spine arising. The lateral-line scales are difficult to recognize.

#### Discussion.

Among the fossil euzaphlegids, *Acanthophleges lessiniae* n. gen. et n. sp. can be separated from *Palimphyes* (upper Paleocene-Oligocene of eastern Europe and middle Asia; e.g., [108]) by having a lower jaw articulation posterior to the mid-point of the orbit (vs anterior in *Palimphyes*; [95]); and short pectoral fins (vs elongate, reaching the level of the second dorsal fin, in *Palimphyes*; [111]; Table 11).

**Table 11:** Summary of the meristic traits of different euzaphlegid taxa.

	*Acanthophleges lessiniae* n. gen. et n. sp.	*Veronaphleges ambrosii* n. sp.	*Veronaphleges brunae*	*Euzaphleges longurio*	*Palimphyes* spp.	*Thyrsocles kriegeri*	*Zaphlegulus venturaensis*
**1st Dorsal fin**	VIII+	XI	IX	X-XII	VII-X	?	XI-XII
**2nd Dorsal fin**	14+	I, 24	I, 14	25-27	I, 14–20	24-26	22-24
**Anal-fin rays**	II, 22	II, 24	II, 14	26-27	II, 15–20	26-27	23-24
**Pectoral-fin rays**	16	20	?	?	13-20	?	?
**Pelvic-fin rays**	I + 5	I + 5	?	?	I + 5	?	?
**Caudal-fin rays (principal)**	?	17 (9 + 8)	17 (9 + 8)	?	17 (9 + 8)	?	?
**Caudal-fin rays (procurrent)**	?	?	?	?	?	?	?
**Vertebrae**	29+	30 (13 + 17)	29 (12 + 17)	51-53	32-39	49-50	47
**Parapophyses**	0	5	2	?	?	?	?
**Branchiostegal rays**	7	5+	?	?	?	?	?
**Epurals**	?	?	2	?	2-3	?	?
**Rayless pterygiophores**	?	1	1	?	3-4	?	?
**Supraneurals**	2	?	3	?	?	?	?

Contains new data and data from [62,108,112].

*Acanthophleges lessiniae* n. gen. et n. sp. also differs from the more recent Miocene euzaphlegids (*Zaphlegulus venturaensis*, *Euzaphleges longurio*, and *Thyrsocles kriegeri*) mainly for having a lower vertebral count (ca. 29 vertebrae vs 47 in *Z. venturaensis,* 51–53 in *E*. *longurio* and 49 or 50 in *T*. *kriegeri*; [62]; Table 11).

*Acanthophleges lessiniae* n. gen. et n. sp. differs from the other euzaphlegid from Solane, *Veronaphleges ambrosii* n. sp., by having a more fusiform and less deep body; seven branchiostegal rays (vs five in *Veronaphleges ambrosii* n. sp.; Table 11); pectoral fins inserted low on the body flanks with 16 rays (vs high on body flanks with 20 rays in *Veronaphleges ambrosii* n. sp.; Table 11); small scales with upright spines (vs larger and spineless in *Veronaphleges ambrosii* n. sp.).

Genus *Veronaphleges* Bannikov, 2008 [108]

**Type species** *Veronaphleges brunae* Bannikov, 2008 [108]

**Emended diagnosis** A genus of the family Euzaphlegidae showing the following combination of features: maximum body depth equal to or slightly less than head length; HL: 26.3–33.7% of SL; stout and massive dentary; lower jaw articulation below the posteriormost border of the orbit; lower jaw slightly prognathous; uniserial and sharp teeth equally large in both jaws; opercle slightly notched posteriorly; hyomandibula almost vertically oriented; vertebrae 29–30; neural spines relatively short and moderately slender; ribs short, strongly inclined; short and slender epineurals; three supraneurals; dorsal fins not widely separated, with one rayless pterygiophore between them; first dorsal fin with nine to 11 spines, second dorsal fin with a spine and 14–24 soft rays; anal fin symmetrical to slightly posterior to the second dorsal fin, with two spines and 14–24 soft rays; pectoral fins not elongated; pelvic fins relatively small, located just behind pectoral-fin insertion; caudal fin large, deeply forked; scales moderately large, cycloid.

**Remarks and comparison** *Veronaphleges* can be referred to the family Euzaphlegidae by having: an elongate body with moderately deep caudal peduncle; mouth large; lower jaw slightly prognathous; jaw teeth uniserial; lack of fangs in both upper and lower jaws; 29–30 vertebrae; parapophyses in the last two to five abdominal vertebrae; two separated dorsal fins with a rayless pterygiophores in between; first dorsal fin with 6–12 slender spines; second dorsal fin longer and more extended, consisting of a single spine and 14–26 rays; anal fin with two spines and 16–27 rays; no finlets; pectoral fins inserted high on the body flanks; pelvic fins thoracic and consisting of one spine and five soft rays; caudal fin forked, without strong hypurostegy [95,108].

*Veronaphleges* cannot be referred to other trichiuroid families (Gempylidae, Trichiuridae) mainly by lacking fangs and by having a substantial gap between the two dorsal fins occupied with a rayless pterygiophore [107].

*Veronaphleges* can be distinguished from other Paleogene euzaphlegids like *Palimphyes* (from upper Paleocene to Oligocene of eastern Europe and middle Asia; e.g., [112]) by having: a strong and stout dentary (vs thinner in *Palimphyes*), lower jaw articulation posterior to the mid-point of the orbit (vs anterior in *Palimphyes*; [95]); lower jaw teeth equal in size to those of the upper jaw, whereas in *Palimphyes* the lower jaw teeth are larger than the premaxillary teeth [108]; 29–30 vertebrae (vs 32–39 in *Palimphyes* spp. [112]); a single rayless pterygiophore in the gap between the two dorsal fins (vs three-four in *Palimphyes*; [112]); distance between the two dorsal fins shorter than the second dorsal-fin base length (vs the opposite in *Palimphyes*; [95]); short pectoral fins (vs elongate, reaching the level of the second dorsal fin, in *Palimphyes*; [111]); moreover, there are additional meristic differences that are listed in Table 11.

*Veronaphleges ambrosii* n. sp. also differs from the euzaphlegids from the Miocene of California (*Zaphlegulus venturaensis*, *Euzaphleges longurio,* and *Thyrsocles kriegeri*) mainly for having a lower vertebral count (29 vertebrae vs 47 in *Z. venturaensis,* 51–53 in *E*. *longurio,* and 49 or 50 in *T*. *kriegeri*; [62]) and for other minor meristic differences in the median fins (see [62]; Table 11). Moreover, it differs from *Z*. *venturaensis* by having a deeper body (BD: 26–30.3% vs 25% of SL in *Z. venturaensis*) and a shorter gap between the two dorsal fins (vs larger in *Z. venturaensis;* see [113]).

*Veronaphleges ambrosii* n. sp. Calzoni, Giusberti & Carnevale

urn:lsid:zoobank.org:act:D8D9A323-5B8A-4DBF-9947-D27BBDF42659

Figs 18-19

**Fig 18:**
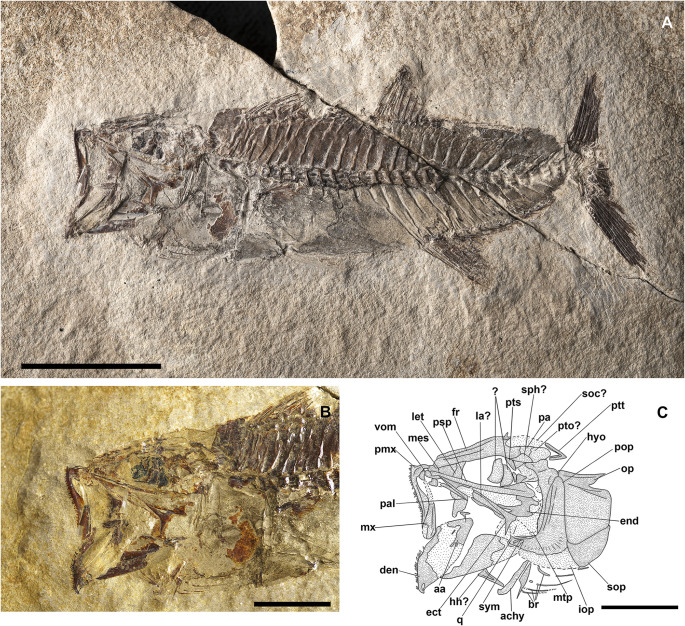
*Veronaphleges ambrosii* n. sp. Lateral view of the holotype, IGVR 64084 **(A)**. Detail of the cranium, coated in alcohol **(B)**. Interpretive reconstruction of the cranium **(C)**. Scale bars 20 mm **(A)**; 10 mm **(B-C)**. Abbreviations: aa: anguloarticular; achy: anterior ceratohyal; br: branchiostegal rays; den: dentary; ect: ectopterygoid; end: endopterygoid; fr: frontal; hh: ventral hypohyal; hyo: hyomandibula; iop: interopercle; la: lachrymal; let: lateral ethmoid; mes: mesethmoid; mtp: metapterygoid; mx: maxilla; op: opercle; pa: parietal; pal: palatine; pmx: premaxilla; pop: preopercle; psp: parasphenoid; pto: pterotic; pts: pterosphenoid; ptt: posttemporal; q: quadrate; soc: supraoccipital; sop: subopercle; sph: sphenotic; sym: symplectic; vom: vomer.

**Fig 19:**
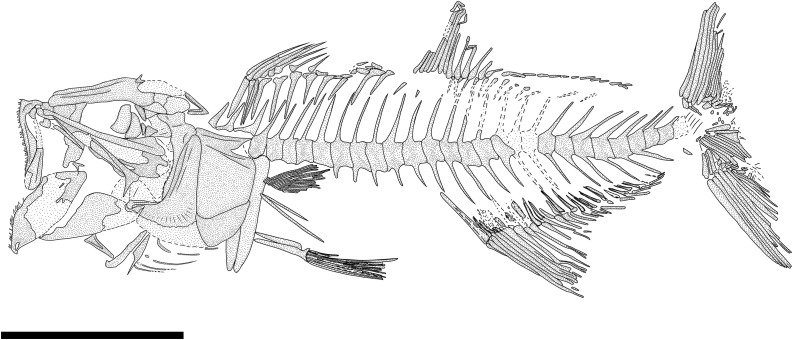
*Veronaphleges ambrosii* n. sp. **Holotype, IGVR 64084. Interpretive reconstruction of the skeleton.** Scale bar 20 mm.

v2011 Euzaphlegidae gen. et sp. nov., Zorzin et al., p. 62, fig. 7. [25]

v2014 Euzaphlegidae gen. indet., Giusberti et al., p.7, fig. 5B. [15]

v2015 Euzaphlegidae, Roghi, p. 58, fig. 2. [114]

**Diagnosis** Head large, representing one third of the body length (HL: 33.7% of SL); supraoccipital crest low; lower jaw stout, slightly prognathous with articulation below the posteriormost portion of the orbit; opercle with moderately deep posterior notch; 30 (13 + 17) vertebrae; five posterior abdominal vertebrae bearing thick parapophyses; first dorsal fin with 11 spines; second dorsal fin with one spine and 24 rays; anal fin with two spines and 24 rays; anal-fin origin posterior to dorsal-fin origin and not symmetrical (PD2: 59% of SL; PA: 69.4% of SL); pectoral fins not elongate, shorter than head length, with 20 rays; pelvic fins thoracic, with one spine plus five rays.

**Etymology** Species named after Luigi Ambrosi, who discovered the Monte Solane site.

**Type locality and horizon** Marly limestones of the Chiusole Formation (CNE5 and E6-E7a Zones; upper Ypresian), Monte Solane (Sant’Ambrogio di Valpolicella, Verona, Italy).

**Holotype (by monotypy)** IGVR 64084, a nearly complete articulated skeleton, 74.7 mm SL.

#### Description.

The body is fusiform and rather deep (Figs 18A and 19), showing its maximum depth at the level of the pectoral girdle (BD: 30.3% of SL; Table 12), tapering posteriorly (CPH: 12.3% of SL; Table 12). The head is large (HL: 33.7% of SL; Table 12) with a wide mouth and an almost elliptic orbit.

**Table 12:** Measurements of *Veronaphleges ambrosii* n. sp. compared to the type species of the genus, *Veronaphleges brunae.*

	*Veronaphleges ambrosii* n. sp.	*Veronaphleges brunae*
**SL (mm)**	74.7	184-192
**TL (mm)**	85.5	?
**HL**	33.7	26.3-27.8
**PD1**	33.2	33-35
**PD2**	59	59-62.5
**PA**	69.4	62-64
**PP**	36.8	?
**PV**	42	?
**DFL1**	21.3	20
**DFL2**	30.8	21-22
**AFL**	25.5	23?
**PFL**	12.1	?
**VFL**	14.6	?
**PRO**	10.9	?
**O**	6.4	?
**POO**	16.9	?
**DRL1**	12.5	?
**DRL2**	12	?
**AFR**	10.8	?
**BD**	30.3	26-28
**CPL**	7.9?	?
**CPH**	12.3	10

It contains new data and data from [108]. Values are as a percentage of SL. Abbreviations: AFL: anal-fin base length; AFR; anal-fin ray length; BD: maximum body depth; CPH: caudal peduncle height; CPL: caudal peduncle length; DFL1–2: dorsal-fin base length; DRL1–2: dorsal-fin ray length; HL: head length; O: orbit diameter; PA: preanal distance; PD1–2: predorsal distance; PFL: pectoral-fin length; POO: postorbital distance; PRO: preorbital distance; PP: prepectoral distance; PV: prepelvic distance; SL: standard length; TL: total length; VFL: pelvic-fin length.

The neurocranium is triangular with a rather elongate ethmoid region. The mesethmoid is trapezoid, and the lateral ethmoid is columnar. The vomer is poorly preserved, and the presence of vomerine teeth is difficult to determine. The frontals are elongate and posteriorly broad. Most of the bones of the skull roof are feebly preserved, mainly as impressions only, such as the parietal, which appears to be small and polygonal in outline (Fig 18B and 18C). The sphenotic and pterotic are both small and subtriangular. The supraoccipital is poorly preserved, and the impression of a low crest is recognizable. The epioccipital is not preserved. The parasphenoid is straight and narrow, with a dorsal flange. The pterosphenoid is only partially exposed along the posterodorsal corner of the orbit, and there is no evidence of the basisphenoid. The nasals and the infraorbital series bones are poorly preserved and extremely difficult to recognize.

The premaxilla has a short and thin ascending process and a low postmaxillary process; it bears 34 small, conical teeth and lacks fangs (Fig 18C). The maxilla is slender and has a slightly expanded distal end characterized by a rounded profile. The dentary is massive and triangular, bearing 17 small and slightly retrorse conical teeth; its outer surface shows a cancellous texture and has an ovoid foramen close to the ventral corner of the symphysis (Fig 18C). The anguloarticular is trapezoid in outline.

The quadrate is fan-shaped and has a developed anterior process. The symplectic is thin and narrow. The ectopterygoid is elongate and slightly curved. The endopterygoid is large and triangular, with rounded edges, while the metapterygoid is poorly preserved. The palatine appears to be thin and delicate. The hyomandibula has a narrow ventral shaft preserved as an impression only, while it presents a broader articular head and a developed opercular process (Fig 18C).

The bones of the opercular series are mainly preserved as an impression only (Fig 18B and 18C). The preopercle is L-shaped with minute serrations along its ventral margin and a vertical arm longer than the horizontal arm. The opercle is trapezoid and has a moderately deep posterodorsal notch. The subopercle is rather deep and has a rounded ventral border. The interopercle is poorly preserved.

The hyoid apparatus is partially preserved (Fig 18C). The anterior ceratohyal is narrow and rectangular, while the posterior ceratohyal is not preserved. There are at least five thin and saber-like branchiostegal rays. The elements of the branchial skeleton are not preserved.

The vertebral column contains 30 vertebrae (13 + 17). The centra are almost square, only slightly longer than high, with rather developed neural prezygapophyses. The last five abdominal vertebrae exhibit well-developed parapophyses. The neural spines are straight and thin. The haemal spines are preserved mostly as impressions and are similar to their opposite neural spines (Fig 19). The ribs are poorly preserved and extremely difficult to describe. Only a few short and delicate epineurals are preserved, attached to the base of the neural spines of the abdominal vertebrae.

The neural and haemal spines of the second and third preural centrum are poorly preserved, as is the caudal skeleton. The caudal fin is forked and contains 17 (9 + 8) principal rays; the inadequate preservation of the fin does not allow to determine the number of procurrent rays.

The number of supraneurals is difficult to determine due to the poor preservation of the predorsal region of the body.

There are two dorsal fins separated by a gap in which a single rayless pterygiophore is accommodated (Fig 19). The first dorsal fin contains 11 spines, supported by 10 wedge-shaped pterygiophores. There is a supernumerary spine on the first pterygiophore (Fig 19). The second dorsal fin contains one small spine and 24 soft rays, supported by 24 thin pterygiophores. The second ray is the longest of the series. The anal-fin origin is posterior to that of the second dorsal fin and contains two small spines followed by 24 soft rays. The first soft ray is the longest of the series.

The posttemporal is deeply bifurcate (Fig 18C). The cleithrum is large with an elongate and distally pointed ventral arm. The ventral postcleithrum is pointed and styliform. The supracleithrum, coracoid, and dorsal postcleithrum are poorly preserved. The pectoral fins are inserted at the mid-height of the body flanks and contain 20 relatively short rays.

The pelvic girdle is thoracic, inserting just below the pectoral girdle. The basipterygium is thin and triangular. The pelvic fin consists of one spine plus five rays that are comparable in length to those of the pectoral fins.

The squamation consists of moderately large and rounded cycloid scales. The scales of the lateral-line series are poorly preserved.

#### Discussion.

*Veronaphleges ambrosii* n. sp. differs from *Veronaphleges brunae* by having a larger head (HL: 33.7% vs 26.3–27.8% of SL in *V*. *brunae*; [108]; Table 12); 30 (13 + 17) vertebrae (vs 29, 12 + 17, in *V*. *brunae*; [108]; Table 11); five parapophyses (vs two in *V*. *brunae*; [108]; Table 11); first dorsal fin with 11 spines (vs nine in *V*. *brunae*; [108]; Table 12), second dorsal fin with one spine and 24 soft rays (vs one spine and 14 soft rays in *V*. *brunae*; [108]; Table 11); anal-fin origin posterior to the second dorsal-fin origin (PD2: 59% and PA: 69.4% of SL vs almost symmetrical to the second dorsal fin in *V*. *brunae*, PD2: 59–62.5% and PA: 62–64% of SL % of SL; [108]; Table 12), anal fin with two spines and 24 soft rays (vs two spines and 14 soft rays *V*. *brunae*; [108]; Table 11).

Family Gempylidae Gill, 1862 [115]

**Remarks:** Ten specimens are referred herein to the family Gempylidae by having: semi-fusiform, elongate and laterally compressed body; elongate mesethmoid and lateral ethmoid; lateral ethmoid with a short lateral process and a posterolateral process; large mouth with fangs in both upper and lower jaws; premaxilla with short ascending process and a reduced postmaxillary process; a small supramaxilla; three branchiostegal rays articulated with the posterior ceratohyal; beryciform foramen absent; a deep opercular notch; a rounded anterodorsal process of the subopercle; between 32 and 50 vertebrae; caudal fin forked; two clearly separate dorsal fins separated by a small notch; supracleithrum elongate; cleithrum without posterior protuberance; pectoral fins inserted low on the body flanks; pectoral-fin length shorter than head length; pelvic fins short [107,109]. Meristic differences that separate the taxa described herein and other Paleogene fossil gempylids are summarized in Tables 13,14 and S2.

**Table 13:** Summary of the meristic traits of different gempylid taxa from Monte Solane.

	*Contemptor mastinoi* n. gen. et n. sp.	*Krampusichthys tridentinus*	*Thyrsitoides cangrandei* n. sp.
**1st Dorsal-fin rays**	VIII+	IX-XIII	XVI-XVII
**2nd Dorsal-fin rays**	I, 28	I-II, 19–26	I, 14
**Anal-fin rays**	II, 24	II, 14–19	II, 11+
**Pectoral-fin rays**	15	15-19	15-19
**Pelvic-fin rays**	I + 5	I + 5	I + 5
**Caudal-fin rays (principal)**	17 (9 + 8)	17 (9 + 8)	17 (9 + 8)
**Caudal-fin rays (procurrent)**	6 + 6	6-7 + 6-8	? + 6
**Vertebrae**	34 (15 + 19)	32 (14 + 18)	34 (18 + 16)
**Branchiostegal rays**	5 + ?	8	7
**Pmx teeth (fangs)**	20 (2-3)	14-21 (1-2)	27 (3)
**Den teeth (fangs)**	5 (4)	7 (3)	11+ (1)

Includes new data and data from [14]. Abbreviations: den: dentary; pmx: premaxilla.

**Table 14:** Measurements of the various gempylid taxa from Monte Solane.

	*Contemptor mastinoi* n. gen. et n. sp.	*Krampusichthys tridentinus*	*Thyrsitoides cangrandei* n. sp.
**SL (mm)**	81.4-84.4	78.2	73.2
**TL (mm)**	96.1-100.4	96.4	78.6
**HL**	28.2-32.4	32.6	23.2
**PD1**	31.1-31.7	39.7	21.9
**PD2**	51.8	61.9	72.5
**PA**	61.1-62.9	63.2	74.6
**PP**	31.9	35.2	23.6
**PV**	37.8-38.1	37.6	25.5
**DFL1**	16.8	18.8	47.8
**DFL2**	32.3	15.5	14.1
**AFL**	20	22.4	?
**PFL**	5.3?	9.5	9.9
**VFL**	8.2-8.9	11.4	8.1
**PRO**	7.8-8.8	13.8	5.6
**O**	6.2-9.9	8.4	3.6
**POO**	11.2-16.4	10.8	19.4
**PRO (%HL)**	31.2	42	33.7
**O (%HL)**	35.1	25.9	27.8
**POO (%HL)**	39.9	32.9	38.9
**DRL1**	5.8	16.5	9
**DRL2**	7.7	14.6	8.8
**AFR**	6.5	12.9	10.7
**BD**	26.7-26.8	30.5	10.9
**CPL**	12.4	13.8	?
**CPH**	11.1-12	14.1	2.9

Includes new data and data from [14]. Values are as percentage of SL. Abbreviations: AFL: anal-fin base length; AFR; anal-fin ray length; BD: maximum body depth; CPH: caudal peduncle height; CPL: caudal peduncle length; DFL1–2: dorsal-fin base length; DRL1–2: dorsal-fin ray length; HL: head length; O: orbit diameter; PA: preanal distance; PD1–2: predorsal distance; PFL: pectoral-fin length; POO: postorbital distance; PRO: preorbital distance; PP: prepectoral distance; PV: prepelvic distance; SL: standard length; TL: total length; VFL: pelvic-fin length.

*Contemptor* n. gen. Calzoni, Giusberti & Carnevale

urn:lsid:zoobank.org:act:85913 CD8-5074-4973-AF47-233F05596FE0

**Type species (by monotypy):** *Contemptor mastinoi* n. gen. et n. sp.

**Diagnosis:** A genus of the family Gempylidae characterized by the following combination of features: elongate, compressed and fusiform body; well-developed supraoccipital crest; upper jaw with 20 small conical teeth and two or three large fangs, one considerably larger than the others; lower jaw with four long fangs and five smaller conical teeth; palatine toothed; smooth ventral edge of the preopercle; moderately deep opercular notch; 34 vertebrae (15 + 19); epineurals attached to elongate and posteriorly curved ribs; five autogenous hypurals and parhypural; three epurals; caudal fin hypurostegic, forked, with 17 principal rays (9 + 8) plus four dorsal and six ventral procurrent rays; first dorsal fin with at least eight spines; second dorsal fin with one spine and 28 soft rays; anal fin with two spines and 24 rays; pectoral fin with 15 rays; pelvic fins with one spine and five rays; moderately large cycloid scales.

**Etymology:** From the Latin word “*Contemptor*”, meaning “contemptuous”, due to the fierce appearance of this taxon.

*Contemptor mastinoi* n. gen. et n. sp. Calzoni, Giusberti & Carnevale

urn:lsid:zoobank.org:act:449FB4AA-6DAE-447A-B672-49DB54722A76

Figs 20-22

**Fig 20:**
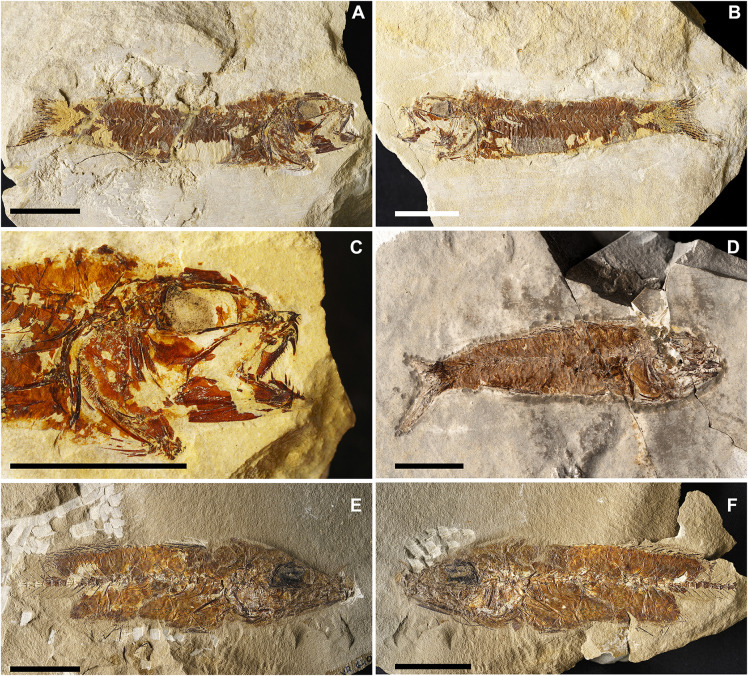
*Contemptor mastinoi* n. gen. et n. sp. Lateral view in natural light of the holotype, IGVR 82435-82436 **(A-B)**, detail of the cranium of IGVR 82345 coated in alcohol **(C)**. Lateral view of the paratype, IGVR 67858, coated in alcohol (D) and of the specimen IGVR 64080a-b in natural light **(E-F)**. Scale bars 20 mm **(A-B, D-F)**, 10 mm **(C)**.

**Fig 21:**
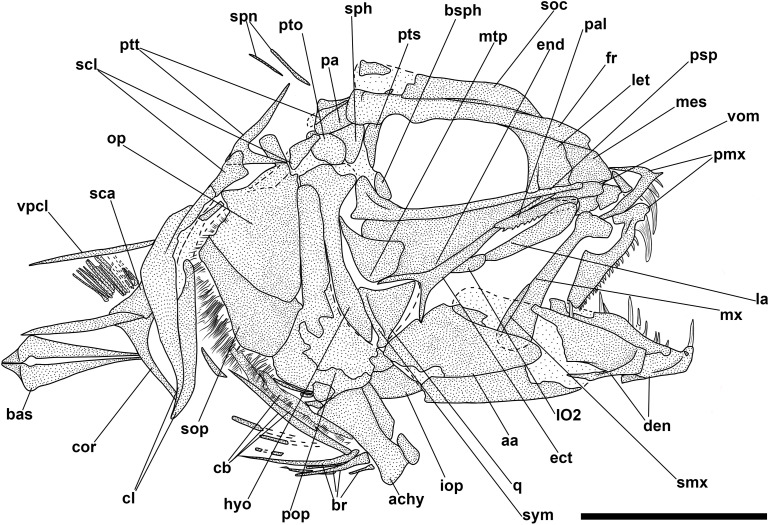
*Contemptor mastinoi* n. gen. et n. sp. **Holotype, IGVR 82435-82436. Interpretive reconstruction of the cranium.** Scale bar 10 mm. Abbreviations: aa: anguloarticular; achy: anterior ceratohyal; bas: basipterygium; br: branchiostegal rays; bsph: basisphenoid; cb: ceratobranchial; cl: cleithrum; cor: coracoid; den: dentary; ect: ectopterygoid; end: endopterygoid; fr: frontal; hyo: hyomandibula; IO2: second infraorbital bone; iop: interopercle; la; lachrymal; let: lateral ethmoid; mes: mesethmoid; mtp: metapterygoid; mx: maxilla; op: opercle; pa: parietal; pal: palatine; pmx: premaxilla; pop: preopercle; psp: parasphenoid; pto: pterotic; pts: pterosphenoid; ptt: posttemporal; q: quadrate; sca: scapula; scl: supracleithrum; smx: supramaxilla; soc: supraoccipital; sop: subopercle; sph: sphenotic; spn: supraneural; sym: symplectic; vom: vomer; vpcl: ventral postcleithrum.

v2011 Gempylidae gen. indet., Zorzin et al., p. 62, fig. 8. [25]

v2014 Gempylidae gen. indet., Giusberti et al., p.7, fig. 5C. [15]

**Diagnosis:** As for genus.

**Etymology:** Species named after “Mastino della Scala” (1220–1277), Master of the city of Verona from 1262 to 1277, and in reference to the Italian word “*mastino*” meaning “mastiff”, given the large fangs of this species.

**Type locality and horizon:** Marly limestones of the Chiusole Formation (CNE5 and E6-E7a Zones; upper Ypresian), Monte Solane (Sant’Ambrogio di Valpolicella, Verona, Italy).

**Holotype:** IGVR 82435–82436, a nearly complete articulated skeleton, in part and counterpart, 84.4 mm SL.

**Paratype:** IGVR 67858, a nearly complete articulated skeleton, 81.5 mm SL.

**Referred materials:** IGVR 64080a-b, an incomplete and articulated skeleton lacking the posterior portion of the body, including the caudal fin, in part and counterpart, is also tentatively referred to this taxon.

#### Description.

The specimens exhibit a moderate size (81.5–84.4 mm SL) with a slightly fusiform body, reaching its maximum depth in the pelvic girdle area, and gradually tapering towards the caudal peduncle (BD: 26.7–26.8% of SL; CPH: 11.1–12% of SL; Fig 20; Table 14). The head is rather large (HL: 28.2–32.4% of SL; Table 14) with an almost ovoid orbit and a large mouth.

The neurocranium is elongate and triangular with a notably developed ethmoid region (Fig 21). The mesethmoid and lateral ethmoid are quadrangular in outline. The frontals are the largest bones of the skull roof; they are elongate, posteriorly broad, and tapered anteriorly. The parietals are small and almost rectangular in outline. The sphenotic is small and forms the posterodorsal corner of the orbit. The pterotic is quadrangular. The epioccipital and the prootic are poorly preserved. The supraoccipital crest is well-developed, extending anteriorly up to the anterior border of the orbit (see IGVR 82435–82436; Figs 20A-C and 21). The parasphenoid is narrow and straight, with a dorsal flange, making up the floor of the orbit. The pterosphenoid and basisphenoid are partially exposed, the latter being small and ovoid, located in the posteroventral corner of the orbit.

The nasals are not preserved. The infraorbital series is incomplete; the lachrymal is elongate and rod-like, while the second infraorbital is short and compact; the other infraorbitals are not preserved (see IGVR 82435–82436; Figs 20A-C and 21).

The premaxilla has a short ascending process and a reduced postmaxillary process; there are two or three fangs, the posterior of which is the largest, and 20 smaller conical teeth (Fig 21). The maxilla has a robust articular head and an expanded and rounded distal end with a thin, splint-like supramaxilla in its posterior portion. The dentary is triangular and bears four fangs and five smaller conical teeth, larger than those of the premaxilla (Fig 21). The outer surface of the dentary exhibits a cancellous texture, and there is a small ovoid foramen close to the ventral portion of the symphysis. The anguloarticular is trapezoid and has a well-developed posterior process.

The quadrate is triangular in outline and has a developed anterior process. The symplectic is small and rod-like. The ectopterygoid is elongate and curved. The endopterygoid is large and triangular. The metapterygoid is quadrangular with a thin posterior process directed upwards. The palatine is large, with rounded margins, bearing small teeth. The hyomandibula has a narrow ventral shaft and a broad articular head.

The preopercle is “L-shaped” and expanded ventrally with a smooth ventral margin (see IGVR 67858; Fig 20D). The opercle is trapezoid with a moderately deep posterodorsal notch. The subopercle is broad and almost arcuate, expanded anteriorly and tapering posteriorly, while the interopercle is roughly triangular in outline.

The hyoid apparatus is partially preserved. The anterior ceratohyal is rectangular and narrow, while the posterior ceratohyal is almost triangular. Seven saber-like branchiostegal rays are preserved. Of the branchial skeleton, several isolated fragments of the ceratobranchials are preserved, bearing up to nine or ten spinous gill rakers and numerous filaments.

The vertebral column contains 34 vertebrae (15 + 19). The centra are rectangular, slightly longer than high. The neural prezygapophyses are particularly developed. The neural spines have a thick basal portion, especially in the anterior abdominal centra. The haemal spines are slender and slightly curved, attached to the anterior portion of the centra. There are thick and posteriorly curved ribs attached to the lateral sides of the abdominal vertebrae, except for the first two. Thin epineurals articulate with the proximal third of the ribs.

**Fig 22:**
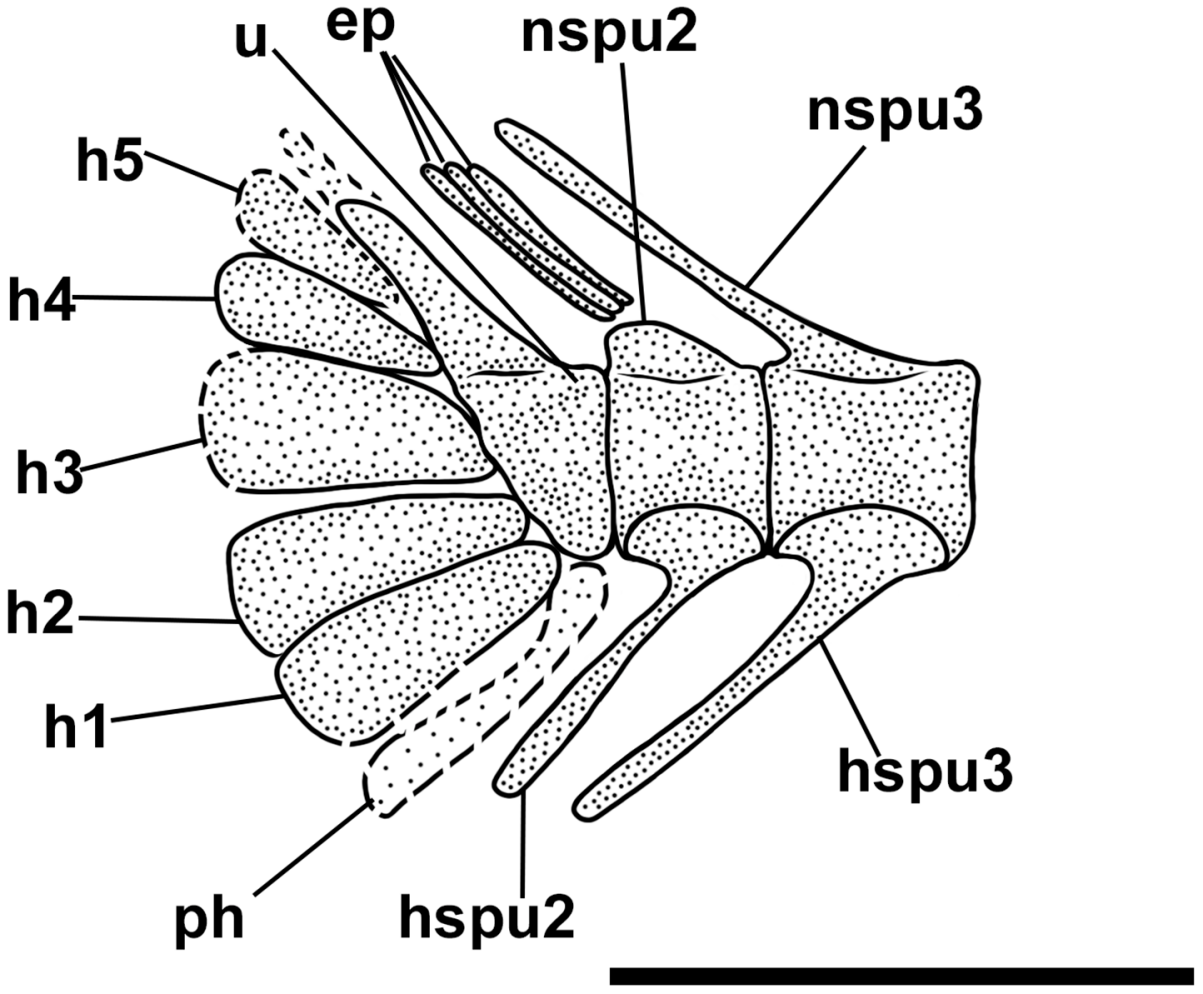
*Contemptor mastinoi* n. gen. et n. sp. **Interpretive reconstruction of the caudal skeleton.** Scale bar 5 mm. Abbreviations: ep: epural; h: hypural; hspu2: haemal spine of the second preural vertebra; hspu3: haemal spine of the third preural vertebra; nspu2: neural spine of the second preural vertebra; nspu3: neural spine of the third preural vertebra; ph: parhypural; u: ural centrum.

The caudal skeleton components are partially hidden due to the extensive hypurostegy of the caudal-fin rays; it consists of five autogenous hypurals, a poorly preserved parhypural, and three epurals, while it is difficult to determine the number of uroneurals (see IGVR 67858; Figs 20D and 22). The haemal spines of the second and third preural vertebrae are autogenous, while the neural spine of the second preural centrum is reduced to a short crest (Fig 22). The caudal fin is forked and includes 17 (9 + 8) principal rays, plus four dorsal and six ventral procurrent rays (4, I, 8 + 7, I, 6).

There are two poorly preserved and thin supraneurals (see IGVR 82435; Figs 20A, 20C, and 21).

The first dorsal fin contains eight spines, supported by seven wedge-shaped pterygiophores, with a supernumerary spine on the first pterygiophore. The second dorsal fin contains one spine followed by 28 rays, supported by wedge-like pterygiophores (see IGVR 67858; Fig 20D). The anal fin originates behind the second dorsal-fin origin and contains two spines followed by 21–24 rays. The first three rays are the longest of the series. The anal-fin pterygiophores are similar to those of the second dorsal fin. There is no trace of finlets.

The posttemporal is deeply bifurcate. The supracleithrum is partially preserved and rod-like. The cleithrum is large with an elongate and pointed ventral end. The coracoid is anteriorly pointed. The scapula is small and quadrangular. The ventral postcleithrum is a straight and pointed process projected posteriorly. The dorsal postcleithrum is not preserved. The pectoral fin inserts low on the body flanks and contains 15 short rays. The pelvic girdle is thoracic, inserting just below the pectoral girdle. The basipterygia are narrow and triangular, with scarcely developed posterior processes (Fig 21). The pelvic fin contains one spine plus five rays.

The squamation consists of moderate-sized cycloid scales. The dorsal branch of the lateral line is ventral to the dorsal fins and above the vertebral column, following the dorsal profile of the body.

In IGVR 67858 (Fig 20D), it is possible to observe some teleost vertebrae as stomachal content.

Genus *Krampusichthys* Calzoni, Giusberti & Carnevale, 2025 [14]

**Type species (by monotypy):** *Krampusichthys tridentinus* Calzoni, Giusberti & Carnevale, 2025 [14]

*Krampusichthys tridentinus* Calzoni, Giusberti & Carnevale, 2025 [14]

Fig 23

+2025 *Krampusichthys tridentinus* Calzoni et al., p. 490, fig. 21. [14]

**Referred materials:** IGVR 64109, a nearly complete articulated skeleton; MGP-PD 33459, a nearly complete articulated skeleton; MGP-PD 33511, a nearly complete articulated skeleton.

#### Discussion.

These specimens are referred to *Krampusichthys tridentinus* (see [14]) by having: a slender and fusiform body; low supraoccipital crest; premaxilla with a short ascending process and one or two fangs associated with 10–12 smaller conical teeth; lower jaw with two fangs and eight smaller conical teeth; outer surface of the dentary with cancellous texture; palatine narrow and toothed; crescent-shaped preopercle with minute serrations on its ventral margin; 32 vertebrae; thin and straight epineurals; caudal skeleton with five autogenous hypurals and parhypural; caudal fin forked with 17 principal rays, plus seven or six dorsal and five or six ventral procurrent rays; first dorsal fin with 10–12 slender spines and second dorsal fin with one spine and 14–17 rays; anal fin with two spines and 18 rays; anal-fin origin located opposite or slightly behind the second dorsal-fin origin; no finlets; pectoral fin short, inserted low on the body flanks and with up to 15 rays; pelvic girdle thoracic; pelvic fins with one spine plus five rays; moderate-sized ctenoid scales (Table 13).

**Occurrence**: upper Ypresian of Solteri (Trento) and Monte Solane (Verona) sites, northeastern Italy.

Genus *Thyrsitoides* Fowler, 1929 [116]

**Type species** *Thyrsitoides marleyi* Fowler, 1929 [116]

**Emended diagnosis** A genus of the family Gempylidae showing the following combination of features: elongate and compressed body (BD: 7.1–12.4% of SL; CPH: 2.9% of SL); snout sharp and prognathous and pointed lower jaw; two or three fangs in the upper jaw; one or two fangs placed anteriorly in the lower jaw; both upper and lower jaws with smaller and conical lateral teeth, those in lower jaw larger than those in the upper jaw; palatine bearing small teeth on its medial surface; 34 vertebrae (18–20 + 14–16); caudal skeleton with five autogenous hypurals, three epurals and two uroneurals; long-based first dorsal fin with 16–19 spines; second dorsal fin with one small spine and 15–17 rays; anal fin smaller than second dorsal fin, containing one or two small spines and 15–17 rays; pectoral fin with 13–16 rays; pelvic fins well-developed, about as long as the pectoral fins with one spine and five rays; two lateral lines, the upper following dorsal contour of body, the lower originating below fourth dorsal-fin spine or slightly behind it, running mid-laterally; body covered with small, thin cycloid scales.

*Thyrsitoides cangrandei* n. sp. Calzoni, Giusberti & Carnevale

urn:lsid:zoobank.org:act:541C6C43-53D4-49B0-AFE1–0FD1F4F0BDA0

Figs 24-26

**Fig 23:**
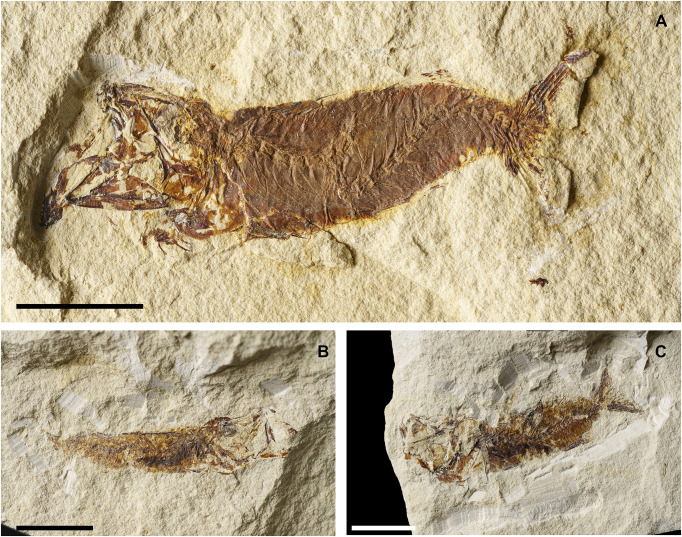
*Krampusichthys tridentinus* Calzoni, Giusberti & Carnevale, 2025 [14]. Lateral view of IGVR 64109 **(A)**, MGP-PD 33459 **(B)**, and MGP-PD 33511 **(C)**. Scale bars 10 mm.

**Fig 24:**
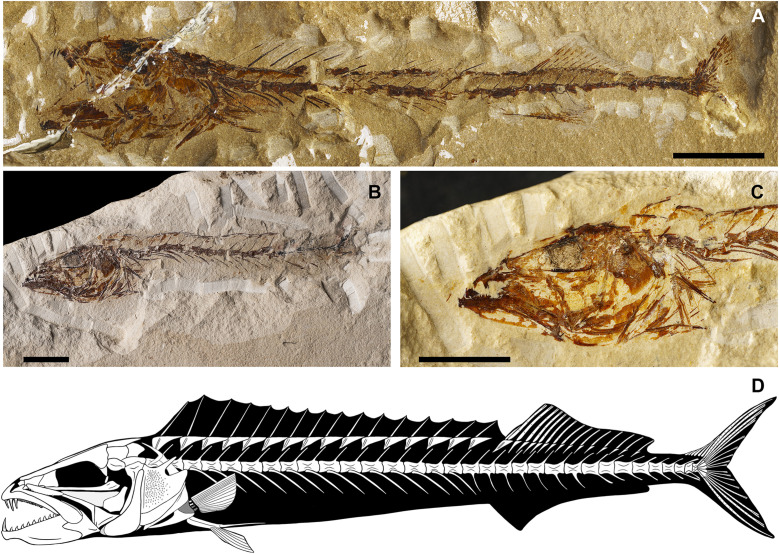
*Thyrsitoides cangrandei* n. sp. Lateral view in natural light and coated in alcohol of the holotype, IGVR 82445 **(A)**. Lateral view of the paratype IGVR 67880, coated in alcohol and detail of the cranium **(B-C)**. Interpretive reconstruction of the skeleton (infraorbital series is omitted; **D)**. Scale bars 10 mm.

**Fig 25:**
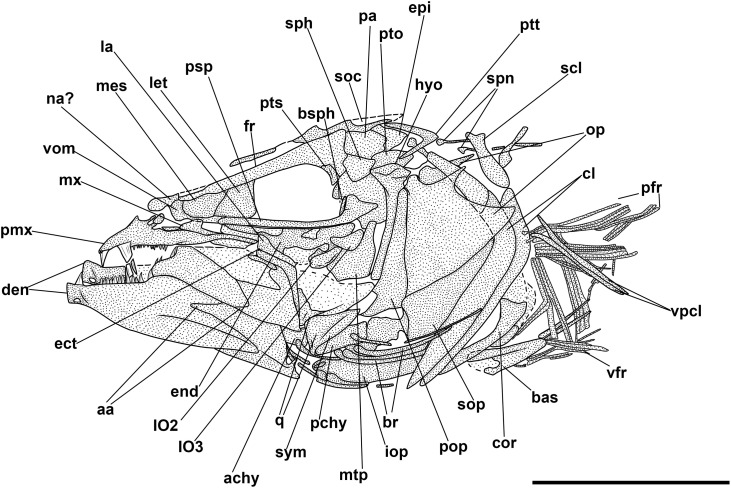
*Thyrsitoides cangrandei* n. sp. **Paratype, IGVR 67780. Interpretive reconstruction of the cranium.** Scale bar 10 mm. Abbreviations: aa: anguloarticular; achy: anterior ceratohyal; bas: basipterygium; br: branchiostegal rays; bsph: basisphenoid; cl: cleithrum; cor: coracoid; den: dentary; ect: ectopterygoid; end: endopterygoid; epi:epioccipital; fr: frontal; hyo: hyomandibula; IO2-3: infraorbital bones; iop: interopercle; la; lachrymal; let: lateral ethmoid; mes: mesethmoid; mtp: metapterygoid; mx: maxilla; na: nasal; op: opercle; pa: parietal; pchy: posterior ceratohyal; pfr: pectoral-fin rays; pmx: premaxilla; pop: preopercle; psp: parasphenoid; pto: pterotic; pts: pterosphenoid; ptt: posttemporal; q: quadrate; scl: supracleithrum; soc: supraoccipital; sop: subopercle; sph: sphenotic; spn: supraneural; sym: symplectic; vom: vomer; vfr: pelvic-fin rays; vpcl: ventral postcleithrum.

v2014 Gempylidae gen. indet. cf. *Thyrsitoides* Giusberti et al., p. 7, fig. 6A. [15]

**Diagnosis:** A small-sized gempylid, characterized by the following combination of features: body remarkably slender and laterally compressed body (BD: 10.9% of SL; CPH: 2.9% of SL); sharply pointed snout; lower jaw pointed and prognathous; premaxilla with three long fangs followed by 27 smaller conical teeth; lower jaw with one large anterior fang followed by at least 10 triangular teeth; 34 vertebrae (18 + 16); caudal fin with five autogenous hypurals and parhypural; first dorsal fin with 16 or 17 spines; second dorsal fin with one spine and 14 rays; anal fin with two small spines followed by at least 11 rays; pectoral fin with 15–17 rays; pelvic fins with one spine and 5 rays; minute cycloid scales.

**Etymology:** Named after Cangrande Della Scala (1291-1329), Master of the city of Verona from 1308 to 1329.

**Type locality and horizon:** Marly limestones of the Chiusole Formation (CNE5 and E6-E7a Zones; upper Ypresian), Monte Solane (Sant’Ambrogio di Valpolicella, Verona, Italy).

**Holotype:** IGVR 82445, a nearly complete articulated skeleton, 73.2 mm SL.

**Paratype**: IGVR 67880, an incomplete articulated skeleton lacking the posterior half of the body.

**Referred materials:** IGVR 64110, an incomplete articulated skeleton lacking the posterior half of the body.

#### Description.

The body is slender and laterally compressed (Fig 24). The head is less than one-fourth of the body length (HL: 23.2% of SL; Table 14), with a slightly ovoid orbit and a large mouth.

The neurocranium is triangular (Figs 24D and 25). The vomer is small and toothless. The mesethmoid and lateral ethmoid are almost trapezoidal in outline. The frontals are large and almost triangular. The parietals are polygonal in outline. The sphenotic is small and triangular, while the pterotic is quadrangular. The bones of the otic and occipital regions are difficult to recognize. The supraoccipital crest is low and partially extends over the frontals. The parasphenoid is straight and narrow. The pterosphenoid is partially exposed, while the basisphenoid is poorly preserved. The nasals are only partially recognizable. The lachrymal is always incomplete and appears to be elongate, while the second and third infraorbitals are much compact.

The premaxilla is thin with a pointed ascending process; it bears three large anterior fangs and up to 27 smaller conical teeth (Figs 24D and 25). The maxilla is thin, with an expanded distal end characterized by a rounded profile. There is a thin and splint-like supramaxilla that articulates with the posterodorsal margin of the maxilla (see IGVR 82445; Fig 24A). The dentary is deep and triangular, bearing a single large anterior fang, followed by at least 11 conical teeth, larger than those of the premaxilla; the outer surface of the dentary exhibits a cancellous texture and a small alveolar ventral foramen close to the anteroventral corner of the symphysis (Fig 25). The anguloarticular is trapezoid with a well-developed posterior process.

The quadrate has a triangular shape with a developed anterior process. The symplectic is slender and narrow. The ectopterygoid is elongate and curved. The endopterygoid is subtriangular, while the metapterygoid is almost quadrangular in outline. The palatine is slightly expanded anteriorly, characterized by rounded margins, and bears eight small teeth (see IGVR 82445; Fig 24A and 24D). The hyomandibula has a narrow ventral shaft and a broad articular head.

The preopercle is crescent-shaped with a smooth ventral margin. The opercle is trapezoid with a broad posterodorsal notch. The subopercle is arcuate, with a rounded anterodorsal process, while the interopercle is almost elliptical.

The hyoid apparatus is partially preserved; the anterior ceratohyal is rectangular and narrow, while the posterior ceratohyal has a triangular shape. There are seven saber-like branchiostegal rays (Fig 25). Of the branchial skeleton, only a few isolated ceratobranchials are preserved, bearing spinous gill rakers and minute gill filaments.

The vertebral column contains 34 vertebrae (18 + 16). The centra are considerably elongate (Fig 24D). The neural prezygapophyses are considerably developed. The neural spines are thin, straight, and elongate, emerging from the posterior half of the centrum. The haemal spines are thin and curved, arising from the midlength of the centrum. There are slender and curved ribs articulated to the lateral sides of the abdominal vertebrae except for the first two. There are no traces of epineurals.

**Fig 26:**
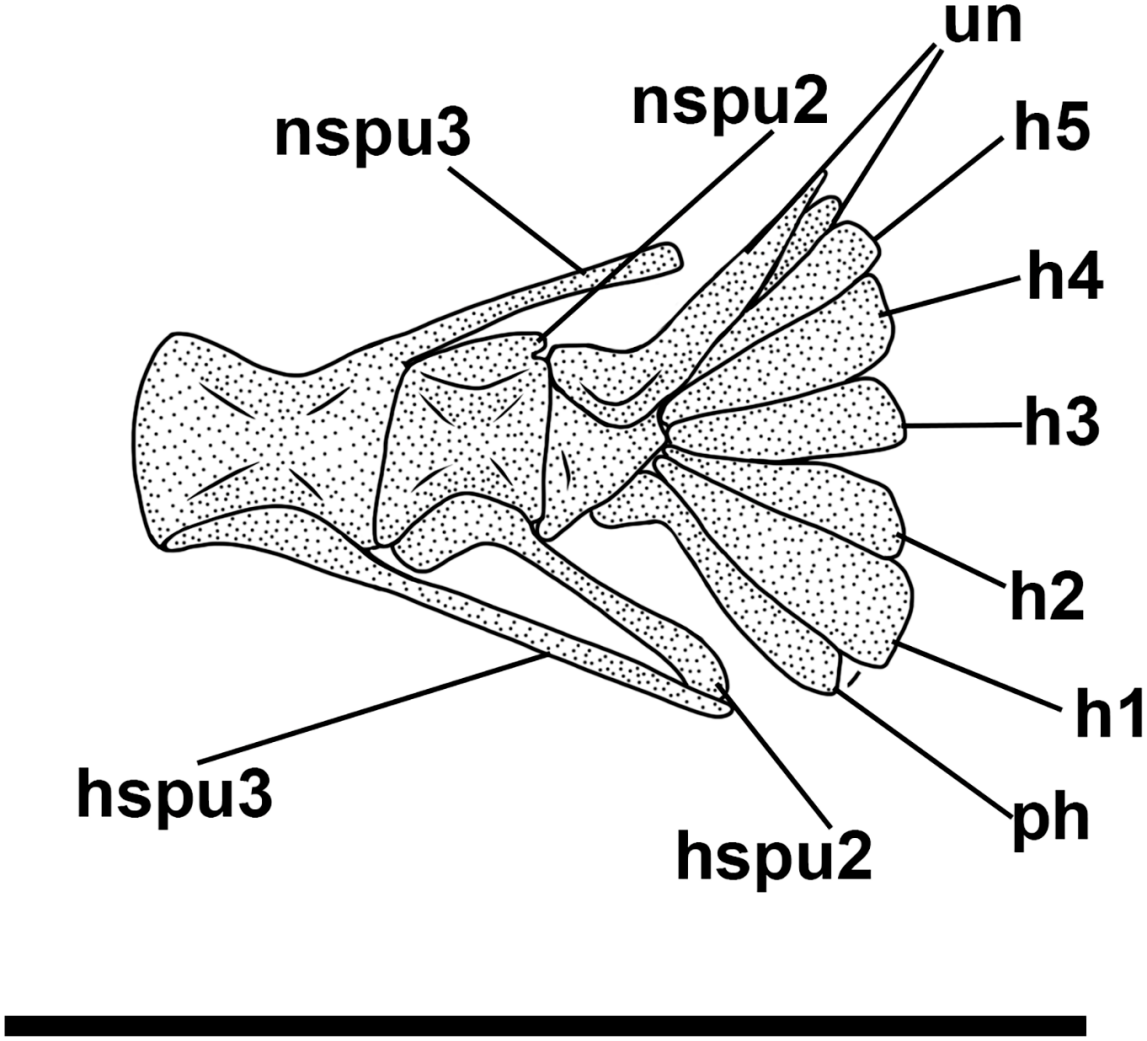
*Thyrsitoides cangrandei* n. sp. **Holotype, IGVR 82445. Interpretive reconstruction of the caudal skeleton.** Scale bar 10 mm. Abbreviations: h: hypural; hspu2: haemal spine of the second preural vertebra; hspu3: haemal spine of the third preural vertebra; nspu2: neural spine of the second preural vertebra; nspu3: neural spine of the third preural vertebra; ph: parhypural; un: uroneural.

The caudal skeleton is composed of five autogenous hypurals, an autogenous parhypural, two uroneurals, and an indeterminate number of epurals (Fig 26). There is a small notch between the second and third hypurals. The haemal and neural spines of the second and third preural vertebrae are autogenous (Fig 26). The caudal fin is forked and bears 17 (9 + 8) principal rays, plus at least six dorsal procurrent rays.

There are two thin and slender supraneurals (see IGVR 67880; Fig 25). The first dorsal fin is extremely elongate; it contains 16 or 17 spines supported by robust and wedge-shaped pterygiophores. Due to inadequate preservation, not all the pterygiophores are preserved, but there is a one-to-one relationship between pterygiophores and their underlying interneural spaces, with the first pterygiophore inserting in the preneural space. The second dorsal fin contains one spine followed by 14 rays, supported by 14 slender and wedge-like pterygiophores. The first and second soft rays are the longest of the series. The anal fin is incomplete; only two short spines and at least eleven rays, supported by thin and almost straight pterygiophores. The anal-fin origin is located slightly behind that of the second dorsal fin. The spines are considerably smaller than the longer successive rays. There are no finlets.

The posttemporal is deeply bifurcate. The supracleithrum is slightly ovoid. The cleithrum is large with an elongate and pointed ventral arm. The coracoid is narrow and pointed, while the scapula is poorly preserved. The ventral postcleithrum is a thin and pointed process projected backwards, while the dorsal postcleithrum is compact and ovoid (see IGVR 82445: Fig 24A). The pectoral fin is short, inserts low on the body flanks, and contains 15–17 rays; only two pectoral-fin radials are preserved.

The pelvic girdle is thoracic, located just below the pectoral girdle. The basipterygia are thin and short: their posterior processes are elongate and pointed (see IGVR 67880; Fig 24B and 24C). The pelvic fins are reduced and bear one spine plus five rays.

The squamation is almost entirely not preserved except for some thin, minute cycloid scales that are visible in IGVR 67880. There is no trace of the lateral-line scales.

#### Discussion.

The main differences between the three genera of gempylids from Monte Solane (*Krampusichthys tridentinus*, *Contemptor mastinoi* n. gen. et n. sp., and *Thyrsitoides cangrandei* n. sp.) are related to the physiognomy of the body, as well as to morphometric and meristic traits. *K. tridentinus* is characterized by a deeper body when compared to the other two taxa, which exhibit a more slender appearance (BD: 30.5% of SL and CPH: 14.1% of SL in *K. tridentinus* vs BD: 26.7–26.8% of SL and CPH: 11.1–12% of SL in *C. mastinoi* n. gen. et n. sp.; and BD: 10.9% of SL and CPH: 2.9% of SL in *T*. *cangrandei* n. sp.; [14]; Table 14). A few differences can be noticed in the cranium, for example, *C. mastinoi* n. gen. et n. sp. shows a rather high and developed supraoccipital crest compared to the other two low-crested taxa. Moreover, the three genera can also be distinguished for their dentition: *K. tridentinus* bears one or two fangs on the premaxilla and up to eight conical teeth in the dentary with three large fangs; the premaxilla of *C. mastinoi* n. gen. et n. sp. bears two or three anterior fangs plus 20 smaller teeth, while the lower jaw bears four fangs and up to five large conical teeth; *T. cangrandei* n. sp. bears up to 27 premaxillary teeth and three large fangs on the premaxilla, while on the dentary has at least 11 conical teeth plus a single anterior fang (see Tables 14 and 15). Another difference between *K. tridentinus* and the other two genera is in the vertebral count (32 vs 34 for both *C. mastinoi* and *T*. *cangrandei* n. sp.; Tables 13 and S2). The main meristic differences can be found in the first dorsal fin, which is remarkably longer in *T*. *cangrandei* n. sp. compared to the other two taxa (16 or 17 spines vs 9–13 in *K. tridentinus* and eight in *C. mastinoi* n. gen. et n. sp.; [14]; Tables 13 and S2). While the second dorsal fin of *C*. *mastinoi* n. gen. et n. sp. contains one spine followed by 28 rays, that of *K. tridentinus* bears one or two spines followed by 18–26 rays, and that of *T*. *cangrandei* n. sp. bears one spine and 14 rays ( [14]; Tables 13 and S2). Finally, *K. tridentinus* and *C. mastinoi* n. gen. et n. sp. share moderately large scales, compared to the minute and thin cycloid scales of *T*. *cangrandei* n. sp.

**Table 15:** Measurements of the various species of the genus *Thyrsitoides.*

	*Thyrsitoides cangrandei* n. sp.	*Thyrsitoides marleyi*	*Thyrsitoides zarathoustrae*
**SL (mm)**	73.2	500-1500	113-181
**TL (mm)**	78.6	?	?
**HL**	23.2	24.4-26.3	16.6-24.7
**PD1**	21.9	22.8	22.1-26
**PD2**	72.5	74.6	?
**PA**	74.6	76.2	80.5-96
**PP**	23.6	25.7	?
**PV**	25.5	28.4	?
**DFL1**	47.8	51.4	54.9-67.4
**DFL2**	14.1	19.5	9.7
**AFL**	?	17.7	7.9
**PFL**	9.9	9.4	?
**VFL**	8.1	7.9	3.5
**PRO**	5.6	11.1	5.3
**O**	3.6	3.8	5.3-7.2
**POO**	19.4	10.7	?
**PRO (%HL)**	33.7	43.8	?
**O (%HL)**	27.8	15	?
**POO (%HL)**	38.9	42.2	?
**DRL1**	9	9.9	?
**DRL2**	8.8	7.9	?
**AFR**	10.7	5.2	?
**BD**	10.9	9.5-12	7.1-12.4
**CPL**	?	1.8	?
**CPH**	2.9	3.1	?

Includes new data and data from [62,107]. Values are as a percentage of SL. Abbreviations: AFL: anal-fin base length; AFR; anal-fin ray length; BD: maximum body depth; CPH: caudal peduncle height; CPL: caudal peduncle length; DFL1–2: dorsal-fin base length; DRL1–2: dorsal-fin ray length; HL: head length; O: orbit diameter; PA: preanal distance; PD1–2: predorsal distance; PFL: pectoral-fin length; POO: postorbital distance; PRO: preorbital distance; PP: prepectoral distance; PV: prepelvic distance; SL: standard length; TL: total length; VFL: pelvic-fin length.

In addition, *T*. *cangrandei* n. sp. differs from its congenerics (the type species *T*. *marleyi* and *T*. *zarathoustrae* from the Eocene of Iran) mainly for meristic and morphometric traits (e.g., shorter first and second dorsal-fin base length, shorter pre- and postorbital distance, see Tables 15 and 16; [62,107]). The main differences with *T*. *zarathoustrae* concern the dentition (three premaxillary and one dentary fangs in *T*. *cangrandei* n. sp. vs two or three premaxillary and two dentary fangs in *T*. *zarathoustrae* [62]) and the first dorsal fin (16–17 spines in *T*. *cangrandei* n. sp. vs 19 in *T*. *zarathoustrae*; [62]; Table 16). As far as the body proportions are concerned, *T*. *cangrandei* n. sp. has a shorter dorsal-fin base (DFL1: 47.8% of SL vs DFL1: 54.9–67.4% of SL in *T*. *zarathoustrae*; [62]; Table 15), a longer second dorsal-fin base (DFL2: 14.1% of SL vs DFL2: 9.7% of SL in *T*. *zarathoustrae*; [62]; Table 15), and a shorter preanal distance (PA: 74.6% of SL vs PA: 80.5–96% in *T*. *zarathoustrae*; [62]; Table 15).

**Table 16:** Summary of the meristic traits of different *Thyrsitoides* species.

	*Thyrsitoides cangrandei* n. sp.	*Thyrsitoides marleyi*	*Thyrsitoides zarathoustrae*
**1st Dorsal-fin rays**	XVI-XVII	XVII-XIX	XIX
**2nd Dorsal-fin rays**	I, 14	I, 16–17	I, 15
**Anal-fin rays**	II, 11+	I, 16–17	I, 15
**Pectoral-fin rays**	15-19	I, 13–14	15
**Pelvic-fin rays**	I + 5	I + 5	I + 5
**Caudal-fin rays (procurrent)**	? + 6	?	?
**Vertebrae**	34 (18 + 16)	34 (20 + 14)	34 (20 + 14)
**Branchiostegal rays**	7	?	?
**Premaxillary fangs**	3	3	2-3
**Dentary fangs**	1	1	2

Includes new data and data from [62,107].

**Fig 27:**
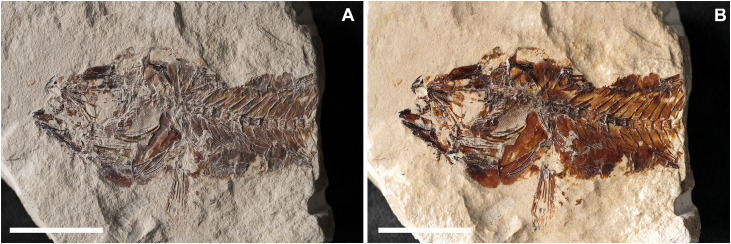
Gempylidae indet. Lateral view of MGP-PD 33557 in natural light (A) and coated in alcohol **(B)**. Scale bars 20 mm.

Gempylidae indet.Fig 27

**Referred materials**: MGP-PD 33557, an incomplete articulated skeleton lacking the posterior portion of the body.

**Remarks:** This specimen is referred to the family Gempylidae by having: elongate mesethmoid and lateral ethmoid; premaxilla with short ascending process; presence of fangs in the upper jaw; a small supramaxilla; a deep opercular notch; a rounded anterodorsal process of the subopercle; two clearly separate dorsal fins; supracleithrum elongate; cleithrum without a posterior protuberance; pectoral-fin length shorter than head length (e.g., [107,109]).

The specimen cannot be referred to Euzaphlegidae by having: a supramaxilla; two premaxillary fangs; lack of rayless pterygiophores between the two dorsal fins; short pectoral fins inserting low on the body flanks [95,108].

The specimen also differs from the Trichiuridae by having a rather deep body (vs extremely thin and compressed in Trichiuridae) and dorsal fins separated by a small gap (vs continuous running across all the body in Trichiuridae; [107,110]).

#### Description.

The specimen measures 59.6 mm from the tip of the premaxilla up to the last preserved vertebra. The head is large with a circular orbit and a terminal mouth (Fig 27).

Most of the elements of the neurocranium are poorly preserved. The mesethmoid and lateral ethmoid are trapezoid in outline. The frontals are large and broad posteriorly. The sphenotic is small and triangular, while the pterotic is almost quadrangular. The supraoccipital seems to have a low crest. The parasphenoid is straight and narrow, while the pterosphenoid and basisphenoid are only partially preserved.

The nasals are small, while the infraorbital bones are not preserved.

The premaxilla has a pointed ascending process and bears two anterior fangs (the first smaller than the second) and several displaced conical and minute teeth. The maxilla has a slender body and articulates a rod-like supramaxilla in its posterior half. The dentary is stout, with an ovoid foramen close to the anteroventral corner of the symphysis and bears numerous minute conical teeth similar to those of the upper jaw. The anguloarticular is poorly preserved.

The suspensorium is generally poorly preserved. The quadrate is triangular in outline, and there is no trace of the symplectic. The ectopterygoid is boomerang-shaped. The palatine is narrow, bearing several small teeth. The hyomandibula, and the endo- and metapterygoid are fragmented.

The preopercle is poorly preserved. The opercle is trapezoid. The subopercle is arcuate with a developed process that overhangs the anterior margin of the opercle, while the interopercle is broad and almost ovoid.

The bones of the hyoid arc are poorly preserved. There are at least seven saber-like branchiostegal rays. Of the branchial arcs, several ceratobranchials are exposed, bearing nine spinous gill rakers and numerous gill filaments.

Only 20 vertebrae are preserved, 13 of which are abdominal. The centra are rectangular, slightly longer than high. The neural spines are straight, thick at the base and with a pointed distal end. The haemal spines are slightly curved and slender. There are curved and slender ribs that articulate with the lateral sides of the abdominal vertebrae except for the two anteriormost. There are thin and straight epineurals attached to the base of the neural spines.

The caudal skeleton and fin are not preserved.

The supraneurals are inadequately preserved. The first dorsal fin contains ten spines supported by nine wedge-like pterygiophores, with the first pterygiophore bearing a supernumerary spine. The second dorsal fin is incomplete and shows only one spine and seven rays, with pterygiophores similar to those of the first dorsal fin. The anal fin is also incomplete, showing two spines and three rays, supported by slender and thin pterygiophores.

The posttemporal is short and rather compact, anteriorly pointed. The supracleithrum is rod-like and straight. The cleithrum is poorly preserved, as are the scapula and coracoid. The pectoral fin inserts low on the body flanks and contains 17 rays. The pelvic girdle and fins are poorly preserved.

The body is covered by moderately sized cycloid scales; there are no traces of the lateral-line series.

#### Discussion.

The specimen differs from the other gempylid taxa of Monte Solane mainly for its deep body and larger head (compared to the slender body of the other taxa, especially *Contemptor mastinoi* n. gen. et n. sp. and *Thyrsitoides cangrandei* n. sp.). Its peculiar dentition, lacking fangs in the lower jaw, sets it apart from all the other taxa, which always show fangs in the dentary. It further differs from *C*. *mastinoi* n. gen. et n. sp. for having epineurals attached to the bases of the neural spines rather than having epineurals attached to the ribs. The scale cover made of moderately large cycloid scales differs from the ctenoid scales of *K*. *tridentinus* and the small and delicate scales of *T. cangrandei* n. sp. [14].

Family Trichiuridae Rafinesque, 1810 [117]

*Eomastix* n. gen. Calzoni, Giusberti & Carnevale

urn:lsid:zoobank.org:act:F201CDB3–2BF4-429C-BE95-B87ECAA5D21C

**Type species (by monotypy):** *Eomastix zabimaru* n. gen. et n. sp.

**Diagnosis:** Small-sized trichiurid characterized by the following combination of features: elongate and ribbon-like body (BD: 8% of SL; CPH: 1.2% of SL); low supraoccipital crest; one large anterior fang in the premaxilla; lower jaw bearing conical teeth and lacking fangs; seven branchiostegal rays; at least 37 vertebrae; caudal fin forked; first dorsal fin with 15 spines and at least 15 rays; anal fin with at least 14 rays; pectoral fins inserting low on the body flanks with 10 rays; pelvic fins reduced with one spine and one ray.

**Etymology:** From the Greek word *“Ηώς”*, meaning “dawn”, also referring to the Eocene epoch, and the Greek word *“μάστιξ”*, meaning “whip”, due to the extremely slender and thin body of this taxon.

**Remarks:** *Eomastix* n. gen. can be referred to the Trichiuridae by having: a remarkably compressed and ribbon-like body; large mouth with prognathous lower jaw; upper jaw with small conical teeth and fangs in the anterior portion; dorsal fins running across all the body with only a small notch between them; soft dorsal-fin base longer than spinous dorsal-fin base; small pectoral fins inserting low on the body flanks; reduced pelvic fins with one spine and one ray; extremely reduced forked caudal fin [107,110].

*Eomastix* n. gen. et n. sp. can be distinguished from several extant trichiurid taxa by having: low supraoccipital crest (vs high in *Assurger*, *Eupleurogrammus*, *Evoxymetopon*, *Lepidopus*, *Tentoriceps*, and *Trichiurus*; [110]); a forked caudal fin (filamentous in *Demissolinea, Eupleurogrammus, Lepturacanthus, Tentoriceps*, and *Trichiurus*; [107,110,118]); developed pelvic girdle and pelvic fin (absent in *Trichiurus* and *Lepturacanthus*; or reduced to scale-like spines in *Aphanopus*, *Evoxymetopon*, and *Lepidopus*; [110]); pelvic girdle thoracic (vs abdominal, well posterior to the pectoral gridle in *Eupleurogrammus* and *Tentoriceps*; [110]).

It also differs from the fossil trichiuroid *Musculopedunculus micklichi* by having fewer vertebrae (37 vs 80 in *M. micklichi*) and a thin caudal peduncle (vs deep and strong in *M. micklichi*; [119]).

*Eomastix zabimaru* n. gen. et n. sp. Calzoni, Giusberti & Carnevale

urn:lsid:zoobank.org:act:2F057330-D9EB-4041-A943-8FEDB8AF5B05

Fig 28

**Fig 28:**
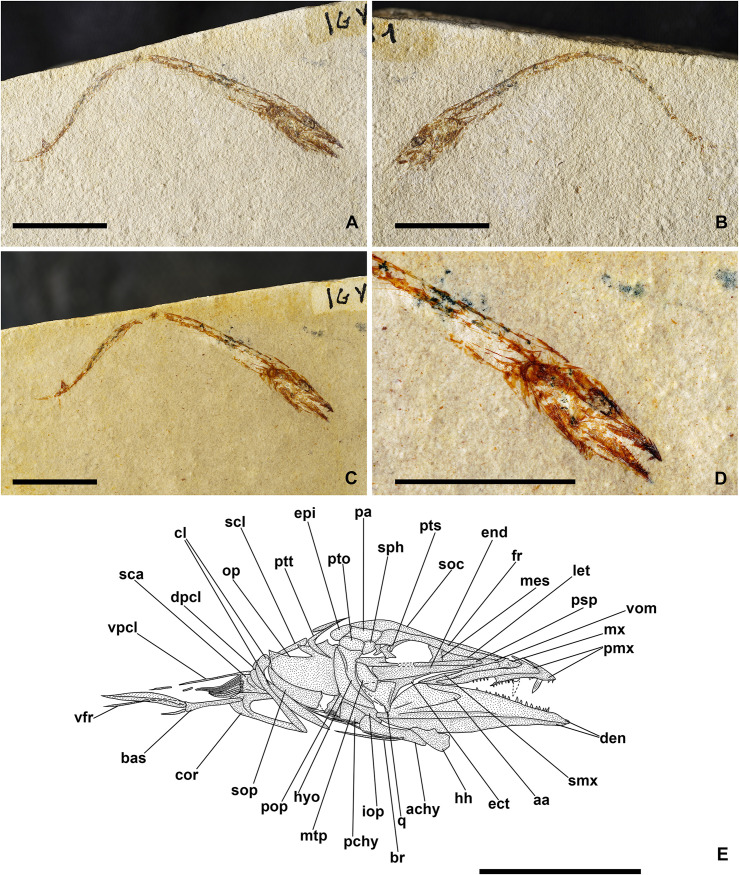
*Eomastix zabimaru* n. gen. et n. sp. Lateral view of the holotype, IGVR 64081-64082, in natural light **(A-B)**, IGVR 64082 coated in alcohol **(C)** and detail of the cranium of IGVR 64082 coated in alcohol **(D)**. Interpretive reconstruction of the cranium **(E)**. Scale bars 10 mm **(A-D)**; 5 mm **(E)**. Abbreviations: aa: anguloarticular; achy: anterior ceratohyal; bas: basipterygium; br: branchiostegal rays; cl: cleithrum; cor: coracoid; den: dentary; dpcl: dorsal postcleithrum; ect: ectopterygoid; end: endopterygoid; epi: epioccipital; fr: frontal; hh: ventral hypohyal; hyo: hyomandibula; iop: interopercle; let: lateral ethmoid; mes: mesethmoid; mtp: metapterygoid; mx: maxilla; op: opercle; pa: parietal; pchy: posterior ceratohyal; pmx: premaxilla; pop: preopercle; psp: parasphenoid; pto: pterotic; pts: pterosphenoid; ptt: posttemporal; q: quadrate; sca: scapula; scl: supracleithrum; smx: supramaxilla; soc: supraoccipital; sop: subopercle; sph: sphenotic; vfr: pelvic-fin rays; vom: vomer; vpcl: ventral postcleithrum.

v2011 Trichiuridae gen. indet. cf. *Anenchelum*, Zorzin et al., p. 62, fig. 9. [25]

v2014 Trichiuridae gen. indet. cf. *Anenchelum*, Giusberti et al., p.7, fig. 6B. [15]

**Diagnosis:** As for genus.

**Etymology:** species named after the Japanese word “*zabimaru*” meaning “snake tail” due to the extremely thin caudal portion of this species.

**Type locality and horizon:** Marly limestones of the Chiusole Formation (CNE5 and E6-E7a Zones; upper Ypresian), Monte Solane (Sant’Ambrogio di Valpolicella, Verona, Italy).

**Holotype (by monotypy):** IGVR 64081-64082, a nearly complete specimen, in part and counterpart, 41.1 mm SL.

#### Description.

*Eomastix zabimaru* n. gen. et n. sp. has a small size (41.1 mm SL) and the body is extremely slender, ribbon-like (Fig 28). The head is not particularly large (HL: 21.6% of SL; Table 17) with an almost elliptic orbit and a large mouth.

**Table 17:** Measurements of *Eomastix zabimaru* n. gen. et n. sp.

	IGVR 64081–64082
**SL (mm)**	41.1
**TL (mm)**	42.2
**HL**	21.6
**PD1**	17.9
**PA**	67.9?
**PP**	22.9
**PV**	28.3
**DFL1**	?
**AFL**	?
**PFL**	6.1
**VFL**	4.7
**PRO**	8.1
**O**	6.4
**POO**	8.3
**PRO (%HL)**	37.4
**O (%HL)**	29.7
**POO (%HL)**	38.6
**DRL1**	7.2
**AFR**	?
**BD**	9
**CPL**	?
**CPH**	1.2

Values are as a percentage of SL. Abbreviations: AFL: anal-fin base length; AFR; anal-fin ray length; BD: maximum body depth; CPH: caudal peduncle height; CPL: caudal peduncle length; DFL1–2: dorsal-fin base length; DRL1–2: dorsal-fin ray length; HL: head length; O: orbit diameter; PA: preanal distance; PD1–2: predorsal distance; PFL: pectoral-fin length; POO: postorbital distance; PRO: preorbital distance; PP: prepectoral distance; PV: prepelvic distance; SL: standard length; TL: total length; VFL: pelvic-fin length.

The neurocranium is triangular, with a particularly narrow and elongate ethmoid region. The lateral ethmoid is almost trapezoid in outline, while the mesethmoid is extremely narrow (Fig 28E). The vomer is thin and toothless. The frontals are elongate and narrow. The parietals are quadrangular. The sphenotic is small and triangular, while the pterotic is subrectangular. The bones of the otic and occipital region are very delicate and extensively fragmented, therefore difficult to recognize. The supraoccipital crest is low and extends anteriorly over the frontals. The parasphenoid is thin and straight. The pterosphenoid is partially exposed in the posterodorsal corner of the orbit. The basisphenoid is not preserved, like the nasals and the bones of the infraorbital series.

The premaxilla has a thin body with a pointed ascending process and reduced postmaxillary processes; it bears a single large anterior fang and at least 11 smaller conical teeth (Fig 28E). The maxilla is slender and articulates with a splint-like supramaxilla along its posterodorsal border. The dentary is narrow and triangular, remarkably prognathous, and bears a series of 17 conical teeth, larger than the premaxillary ones (Fig 28E). The anguloarticular is quadrangular, while the retroarticular is minute and compact.

The quadrate is triangular, bearing a well-developed posterodorsal process. The symplectic is thin and rod-like. The ectopterygoid is thin and curved. The endopterygoid is triangular and narrow, while the metapterygoid is quadrangular in outline. The palatine is oblong and bears minute teeth along its ventral edge (see IGVR 64081; Fig 28D and 28E). The hyomandibula has a narrow ventral shaft and a well-developed articular head.

The preopercle is crescent-shaped. The opercle is trapezoid and large with a deep posterodorsal notch. The subopercle is poorly preserved and arcuate, while the interopercle is almost elliptical.

The hyoid apparatus is well preserved. The hypohyals are knob-like and compact. The anterior ceratohyal is narrow, lacking a beryciform foramen, while the posterior ceratohyal is almost triangular. There are seven slender and saber-like branchiostegal rays. Of the branchial skeleton, only fragments of isolated ceratobranchials are preserved.

The vertebral column contains 37 vertebrae (18 + 19). The centra are elongate, longer than high. The neural prezygapophyses are particularly developed. The neural spines are thin and straight, while the haemal spines are thin and curved and highly bent in the posterior-most caudal vertebrae. There are slender and curved ribs articulated to the lateral sides of the abdominal vertebrae except for the first two. There is no trace of the epineurals.

The caudal skeleton and caudal fin are reduced and poorly preserved. The haemal spines of the second and third preural vertebrae are thin, almost adherent to the centrum and autogenous, while the neural spines are extremely reduced. It is possible to notice a small, forked caudal fin, even if the number of the caudal-fin rays is difficult to establish (at least 12); the structure of the caudal skeleton cannot be determined due to inadequate preservation.

The supraneurals are poorly preserved, and their original number is difficult to determine.

There is a single continuous dorsal fin running along the whole body. The spinous portion of the dorsal fin contains 15 spines supported by robust, wedge-shaped pterygiophores; there is no supernumerary spine on the first pterygiophore, which inserts in the preneural space, and there is a one-to-one relationship between the successive pterygiophores and the underlying interneural spaces. The soft dorsal fin is incomplete and contains at least 15 rays. The anal fin is also incomplete, and only 14 rays are preserved; only a few anal-fin pterygiophores are preserved, being thin and slender. The anal-fin origin is almost opposite to that of the soft dorsal fin.

The posttemporal is deeply bifurcate. The supracleithrum is thin and rod-like. The cleithrum is crescent-shaped, large, and with an elongate and distally pointed ventral portion. The coracoid is curved and anteriorly pointed. The scapula is small and compact. The ventral postcleithrum is styliform and elongate, while the dorsal postcleithrum is minute and triangular. The pectoral fin is short, inserting low on the body flanks, and contains ten rays, supported by four pectoral-fin radials.

The pelvic girdle is thoracic, inserting just below the pectoral girdle. The basipterygia are thin and anteriorly elongate; their posterior process is rather developed. The pelvic fins consist of a large and posteriorly developed spine and a short and poorly preserved ray. The squamation is not preserved, and there is no trace of the lateral-line series.

#### Discussion.

The specimen was attributed to Trichiuridae gen. indet. cf. *Anenchelum* by Zorzin et al. [25] and Giusberti et al [15]. However, *Eomastix zabimaru* n. gen. et n. sp. differs from *Anenchelum* by having a greater body depth (BD: 9% of SL vs 5.6% of SL in *Anenchelum*; Table 17; see [120]); fewer vertebrae (37 vs 76–119 in *Anenchelum*; [120,121]; Table 18); first anal-fin pterygiophore showing the same size of the successive pterygiophores (vs first pterygiophore considerably larger in *Anenchelum* [121]); pectoral fins short with 10 rays (vs highly elongated with 15–17 rays in *Anenchelum*; [120,121]; Table 18); pelvic girdle is directly below the pectoral girdle (vs markedly posterior to the pectoral fin base in *Anenchelum*; [120,121]).

**Table 18:** Summary of the meristic traits of *Eomastix zabimaru* n. gen. et n. sp. compared with different trichiurid taxa.

	*Eomastix zabimaru* n. gen. et n. sp.	*Anenchelum*
**1st Dorsal-fin rays**	XV	XXXV
**2nd Dorsal-fin rays**	15+	74-77
**Anal-fin rays**	14+	I-II, 35–68
**Pectoral-fin rays**	10	15-17
**Pelvic-fin rays**	I + 1	I + 0
**Caudal-fin rays (principal)**	12?	17 (9 + 8)
**Caudal-fin rays (procurrent)**	?	?
**Vertebrae**	37 (18 + 19)	76-119
**Branchiostegal rays**	7	?
**Premaxillary teeth (fangs)**	11+ (1)	? (2)
**Dentary teeth (fangs)**	17 (0)	13 (1)

Includes new data and data from [111,120,121].

The most ancient trichiurids known in the fossil record are *Trichiurides* and *Eutrichiurides* (Paleocene to middle Eocene), even though they are known only by isolated teeth [122], thus making *E. zabimaru* n. gen. et n. sp. the oldest articulated skeleton of a trichiurid known in the fossil record.

Order Gobiiformes *sensu* Thacker, 2014 [123]

Suborder Apogonoidei *sensu* Thacker, 2014 [123]

Family Apogonidae Günther, 1859 [124]

Subfamily Apogoninae Günther, 1859 [124]

Fig 29

**Fig 29:**
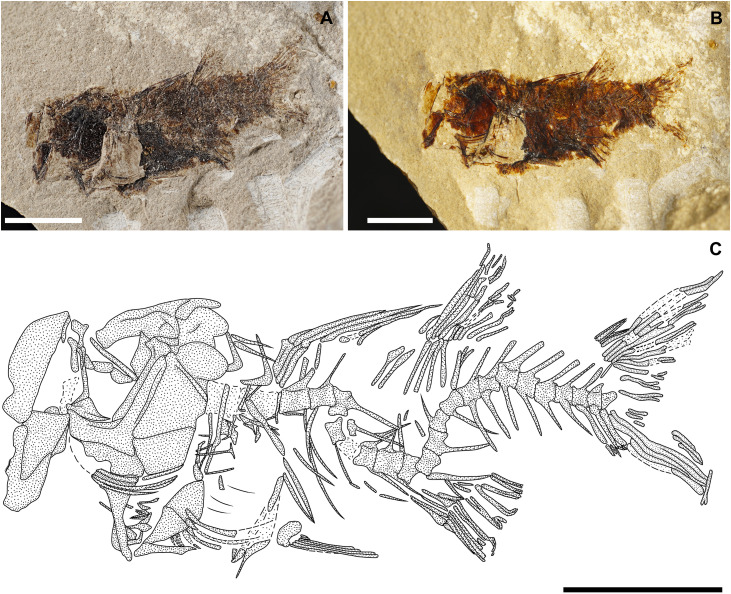
Apogoninae indet. Lateral view of IGVR 82413 in natural light **(A)** and coated in alcohol **(B)**. Interpretive reconstruction of the skeleton of IGVR 82413. Scales are omitted **(C)**. Scale bars 5 mm.

v2014 Apogonidae gen. indet., Giusberti et al., p. 7, fig. 5A. [15]

**Referred materials** IGVR 82413, a nearly complete articulated skeleton.

**Remarks** This specimen is tentatively referred to the Apogonidae by having: small size (under 70 mm); body short, oblong and compressed; head and eyes large; mouth terminal; jaws with small villiform teeth; seven branchiostegal rays; 23 vertebrae; haemal spines of the second and third preural centra autogenous; caudal fin forked with 17 principal rays; two separate dorsal-fin lobes; first dorsal fin with six spines; second dorsal fin with one spine and nine rays; one supernumerary dorsal-fin spine; anal fin with two spines (the first smaller than the second) and ten rays; first anal-fin ray segmented and branched; pelvic fin with one spine and five rays; cycloid scales also covering the cheek (e.g., [125–127]).

#### Description.

The specimen has a total length of 19.7 mm TL measured from the anterior tip of the head to the distal tip of the caudal-fin rays. The body is short and compact, with a large head and rounded orbit (Fig 29).

The neurocranium is compact but poorly preserved. The ethmoid region is not preserved. The frontals are the largest bones of the skull roof, slightly expanded posteriorly. The sphenotic is minute. The supraoccipital bears a moderately developed crest. The other elements of the neurocranium are poorly preserved and difficult to recognize. The parasphenoid is thin and straight. The nasals are poorly preserved, and some fragments of the anterior infraorbital bones are preserved but are poorly exposed partially hidden by the scale covering.

The jaws are incomplete and displaced from their original position. The upper jaw is almost totally missing, with no traces of premaxilla and only the anteriormost portion of the maxilla preserved. The dentary is incomplete, and the anguloarticular is triangular in outline.

The quadrate is fan-shaped. The symplectic is thin and rod-like. The ectopterygoid is mostly hidden and appears rather short and arcuate. The endo- and metapterygoid are hidden by the scales and poorly preserved. The palatine is small, and part of its anterior articular process is noticeable. The hyomandibula has a narrow ventral shaft, a broad articular head, and a tiny opercular process.

The preopercle is L-shaped, with a rather expanded horizontal arm, a weakly crenulate ventral margin, and a smooth posterior edge. The opercle is large, with a somewhat developed spine. The subopercle and interopercle are partially hidden under the other elements of the opercular series and difficult to describe.

The hyoid apparatus is partially disarticulated. The hypohyals are not preserved. The anterior ceratohyal is narrow and rectangular and lacks a beryciform foramen, while the posterior ceratohyal is triangular. There are seven saber-like branchiostegal rays. Of the branchial skeleton, some fragmented ceratobranchials are preserved.

The vertebral column comprises at least 23 vertebrae (9 + 14). The centra are squared and compact. The neural spines are thin and straight, attached to the anterior portion of the centrum. The haemal spines are slender, almost identical to their neural counterparts. Some elongate and thin ribs are preserved, articulated with the lateral sides of the abdominal vertebrae. There are no traces of epineurals.

The structure of the caudal skeleton is difficult to interpret due to inadequate preservation. The haemal spines of the second and third preural centrum are autogenous. The neural spine of the second preural centrum is reduced. The caudal fin is weakly forked and bears 17 (9 + 8) principal rays plus at least five dorsal and ventral procurrent rays.

There are two flimsy and pointed supraneurals. There are two dorsal fins with separate lobes. The first spinous dorsal fin contains six spines of similar length, with a small supernumerary spine on the first pterygiophore. The first dorsal fin is supported by thin and delicate pterygiophores. The second dorsal fin contains one spine and nine rays, with the first ray being the longest of the series; it is supported by thin pterygiophores similar to those of the first dorsal fin. The anal fin contains two small spines followed by at least nine rays, supported by thin pterygiophores similar to those of the dorsal fins. Due to poor preservation of the vertebral column, it is not possible to determine the pterygiophore formulae.

The pectoral girdle is almost completely not exposed. The posttemporal is bifurcate. The supracleithrum is thin and rod-like. Of the cleithrum, only its posterior portion is visible. The ventral postcleithrum is slender and pointed, almost reaching the ventral margin of the body. The scapula, coracoid, and pectoral fin are not preserved.

The pelvic girdle is thoracic, placed not far behind the pectoral girdle. The basipterygium is almost completely not preserved. However, the pelvic fin is visible and rather short, containing one spine and five rays.

The body is fully covered with moderate-sized cycloid scales, extending also in the cranial region and covering the opercular series and the suspensorium. The scales of the lateral-line series are not recognizable.

#### Discussion.

The specimen cannot be assigned to the subfamily Amioidinae by having 17 principal caudal-fin rays (vs 15 in Amioidinae; [3]). It cannot be referred to the subfamily Paxtoninae by having two separate dorsal-fin lobes (vs single and continuous in Paxtoninae; [3]). As far as the subfamily Pseudamiinae is concerned, its distinctive characters cannot be observed in the examined specimen if not for the body physiognomy, which is substantially different (more compact vs slender in Pseudamiinae; [3]).

The specimen might be referred to the subfamily Apogoninae by having number of median and pelvic-fin rays consistent with those characteristics of the genera of this subfamily, as well as by having cycloid scales, also in the head region (see [127]; Table 19). The alignment of our specimen to the extinct tribe Eoapogonini (Table 20; comprising *Arconiapogon*, *Eoapogon*, *Bolcapogon*, and *Apogoniscus*) is prevented by the composition of its anal fin, comprising two spines and nine rays (vs two spines and six or seven rays in Eoapogonini; [128]; Table 20), and also by the possession of a forked caudal fin (vs rounded in all Eoapogonini except *Apogoniscus*; [128]; Table 20) and a smooth posterior edge of the preopercle (vs serrate in all Eoapogonini except *Apogoniscus*; see [3]; Table 20). Among the Apogonini assigned to indeterminate tribes, our specimen differs from *Leptolumamia vetula* by having different dorsal fin formulae (VI + I, 9 vs VII + I,10 in *L*. *vetula*; [129]; Table 20); separated dorsal-fin lobes (vs connected in *L*. *vetula*; [129]; Table 20); five ventral and dorsal procurrent rays (vs nine dorsal and eight ventral procurrent rays in *L*. *vetula*; [129]; Table 20); 23 vertebrae (vs 24 in *L*. *vetula*; [129]; Table 20); and cycloid scales (vs ctenoid in *L*. *vetula*; [129]; Table 20). It also differs from *Eosphaeramia* by having 23 vertebrae (vs 25 in *Eosphaeramia*; [130]); two separate dorsal-fin lobes (vs connected in *Eosphaeramia*; [129]; Table 20); six spines in the first dorsal fin (vs seven in *Eosphaeramia*); first dorsal fin low (vs remarkably high in *Eosphaeramia*; [130]; Table 20); short pelvic fins (vs exceptionally long in *Eosphaeramia*; [130]; Table 20). The lack of preserved cranial characters useful for an attribution at the tribe level prevents us from assigning this specimen to a specific taxon and is therefore referred to as Apogonini tribe and gen. indet.

**Table 19:** Meristic and characteristic traits of the Subfamily Apogoninae compared to IGVR 82413.

	IGVR 82413	Subfamily Apogoninae
**Dorsal fin**	VI + I, 9	VI-VIII+I, 9–13
**Dorsal-fin lobes**	separate	separate
**Anal-fin rays**	II, 9	II, 8–18
**Pelvic-fin ryas**	I + 5	I + 5
**Caudal-fin rays (principal)**	17 (9 + 8)	17 (9 + 8)
**Caudal-fin margin**	Forked	Forked, truncate, slightly rounded
**Posterior edge of preopercle**	Smooth	smooth/serrated
**Supraneurals**	2	0-3
**Supernumerary dorsal-fin spines**	1	1-2
**Scales**	Cycloid	cycloid, ctenoid, spinoid, absent

Includes new data and data from [3,125,127,131].

**Table 20:** Summary of the morphological and meristic traits of IGVR 82413 compared with different genera of fossil apogonids.

	Apogoninae indet.	Tribe Eoapogonini				Tribe indet.	
	IGVR 82413	*Apogoniscus*	*Arconiapogon*	*Bolcapogon*	*Eoapogon*	*Eosphaeramia*	*Leptolumamia*
**1st Dorsal-fin rays**	VI	VI	VI	VII	VIII	VII	VII
**2nd Dorsal-fin rays**	I, 9	I, 8	I, 9	I, 9	I, 9	I, 9	I, 10
**Dorsal-fin lobes**	separated	separated	separated	separated	connected	connected	connected
**Anal-fin rays**	II, 9	II, 6	II, 6	II, 7	II, 6	II, 9	II, 9
**Pelvic-fin rays**	I + 5	?	I + 5	?	I + 5	I + 5	I + 5
**Pelvic-fin length**	short	short	Short	short	short	long	short
**Caudal-fin rays (principal)**	17 (9 + 8)	17 (9 + 8)	17 (9 + 8)	17 (9 + 8)	17 (9 + 8)	17 (9 + 8)	17 (9 + 8)
**Caudal-fin rays (procurrent)**	5 + 5	9 + 8	?	7-8 + 6-7	9 + 8	7-8 + 7-8	9 + 8
**Caudal-fin margin**	forked	forked	rounded	rounded	rounded	forked	forked
**Vertebrae**	23 (9 + 14)	24 (10 + 14)	?	24 (10 + 14)	24 (10 + 14)	25 (10 + 15)	24 (10 + 14)
**Posterior edge of preopercle**	smooth	smooth	serrate	serrate	serrate	smooth	smooth
**Scales**	cycloid	cycloid	cycloid	cycloid	ctenoid	cycloid	ctenoid
**Branchiostegal rays**	7	7	2-3	?	7	7	?

Includes new data and data from [3,128–130].

### Teleostei indet

#### Description.

32 specimens from Monte Solane are only referred to as indeterminate teleosts due to their poor preservation. It is not possible to achieve a more precise taxonomic placement for these specimens, which are usually small-sized and often disarticulated or fragmented. There are a few large specimens consisting of isolated elements of considerable size, as for example, MGP-PD 33559, which consists of a large ctenoid scale (2.5 cm in length) showing five radii and clearly visible primary and secondary circuli; in addition, the anterior portion of the scale appears to bear several small ctenii.

## Discussion

### The paleoenvironment of the Monte Solane *Lagerstätte*

**Fig 30:**
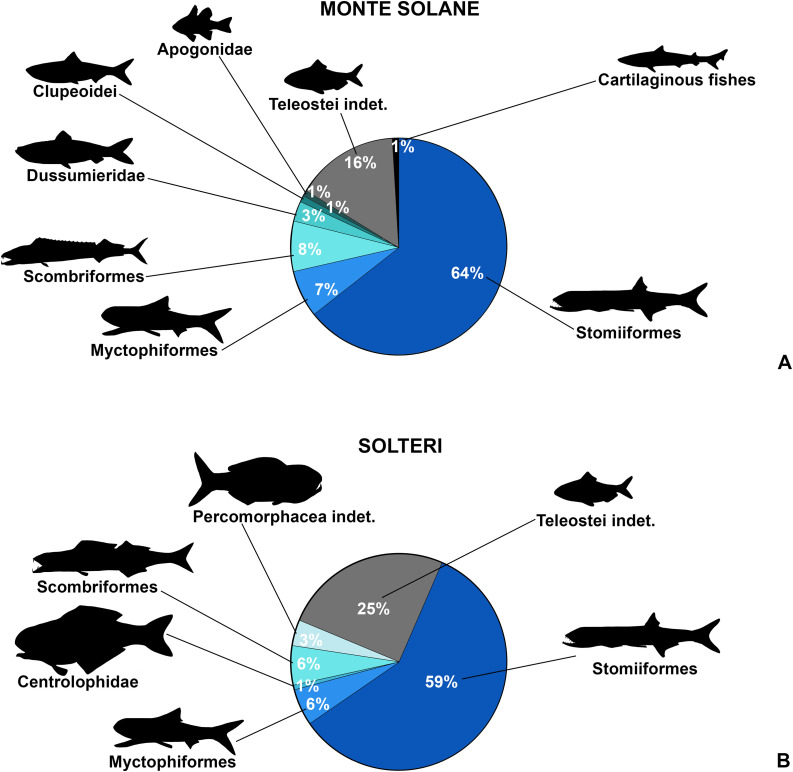
Comparison between the taxonomic composition of the ichthyofauna of the Monte Solane (A) and Solteri (B) sites. Graph modified and reprinted from [14] under a CC BY license, with permission from Rivista Italiana di Paleontologia e Stratigrafia, original copyright year 2025.

The Monte Solane ichthyofauna unquestionably reflects an ecosystem dominated by mesopelagic taxa. Stomiiforms account for about two-thirds of the specimens (64%), mainly gonostomatids and, to a lesser extent, phosichthyids. Myctophiforms are less abundant (7%), while, among percomorphs, the most common are scombriforms (8%), represented by gempylids, a few euzaphlegids, and a single trichiurid (Fig 30A). Given the bathyal setting and coeval age of both deposits (see also below), the assemblages of Monte Solane and Solteri, unsurprisingly, are broadly similar (Fig 30), sharing several taxa (e.g., *Scopeloides violator*, *S. bellator*, *Eomyctophum mainardii*, and *Krampusichthys tridentinus* [14]). The main difference between Solteri and Monte Solane is the occurrence at Monte Solane of a single shark remain and of sardines, round herrings, and a cardinalfish (Fig 30). The latter taxa (ca. 5% of the entire assemblage; Fig 30A) are epipelagic and benthic fishes, therefore pertaining to a neritic environment. Their low abundance supports the hypothesis already proposed by [15] that such taxa were transported horizontally into the basin from a shallow-water setting. They are, along with red algae and other fossils recovered in the laminites, a component of the marked “neritic input” characterizing the Monte Solane site, well epitomized by the dominant presence of lithologies derived from shallow-water carbonate detritus (e.g., biocalcarenites and larger foraminiferal turbidites) sourced by a nearby platform [15].

Based on the composition of the fish assemblage it can be hypothesized that the Monte Solane site could be indicative of a paleobiotope characterized by a depth comparable to the Solteri site, which reflects a bathymetry not beyond 400 m [14]. Giusberti et al. [15] suggested a 300–600 m paleodepth range for the Monte Solane site, combining the preliminary data from the fish and the foraminiferal indices. Hence, by considering the depth range of the extant groups of fishes found in the Monte Solane site, and with all necessary caution in applying taxonomic uniformitarianism, we can attempt a refinement of this estimate, considering that gempylids (which comprise around 6% of the Monte Solane ichthyofauna) show a bathymetric range from 200 to 500 m [107]. Therefore, it is reasonable to hypothesize that the fossiliferous deposits of Monte Solane originated at a paleodepth comprised between 300 and 500 m.

### Taphonomic remarks

There is no field information available about the specimens found at the Monte Solane site, as most of the fish were collected during prospections of the site without specific controlled excavations. Nevertheless, it is still possible to discuss some taphonomic aspects of the fossil-bearing interval of this site and to compare it with the coeval Solteri site. The Monte Solane specimens are moderately well-preserved and mostly articulated, with a few cases of fragmentary or extensively disarticulated skeletons; as a matter of fact, only 16% of the specimens were referred to as indeterminate teleosts (while at Solteri these were more than one quarter of the finds; [14]). It is often possible to observe soft tissues preserved in the examined material, showing various organs preserved as a thin, black organic film (e.g., photophores, eyeballs, and sometimes even part of the viscera). About 31% of the specimens can be identified at the species level, with the best-preserved specimens being small-sized fishes, usually under ten cm SL; large individuals are rare and usually fragmented. A common feature of the Monte Solane and Solteri fishes resides in the alteration of their vertebral column, which in most of the deformed fishes shows an undulate and irregular pattern (54.7% and 47% respectively), while the body outline of the fish remains unaltered (for details on the nature of these types of backbone deformations, see Calzoni et al. [14]). Conversely, concave or convex-bent backbones are extremely rare in both sites (less than 10% combined in both sites). Despite Monte Solane presenting a higher percentage of well-preserved fishes, it is particularly puzzling to see how the number of specimens with deformed backbones is higher than that of the Solteri site (54.7% vs 47%, respectively), while the specimens with no deformation are notably fewer than in Solteri (35% vs 45%).

### The Monte Solane record and its evolutionary implications for the Paleogene ichthyofaunas

The Monte Solane fish assemblage, along with that of the Solteri site, provides a clearer picture of the paleodiversity and evolutionary history of some teleost groups that are scarcely represented in the lower Paleogene skeletal record (e.g., stomiiforms, myctophiforms, and certain scombriforms), extending back their stratigraphic range and expanding their geographical distribution.

Among the stomiiforms, the Gonostomatidae are mostly known from the Oligocene of eastern Europe (see [68,89,95,132–134]), with the previously earliest known representative of the family being *Primaevistomias weitzmani* from the Bartonian of Gorny Luch (Russia; [135]). The newly described species from the Ypresian of northern Italy (*Scopeloides violator* and *S*. *bellator*), both occurring at Monte Solane and Solteri [14], extend back the record of this family, being its earliest representatives [14]. In addition, a third species from Monte Solane might represent the earliest known appearance of *S*. *glarisianus*.

The Phosichthyidae are mostly found in the Oligocene of Europe [73,74,95] and in the Miocene deposits of Europe and the North Pacific (see [68,136–138]). The earliest representative of the family previously reported was *Vinciguerria distincta* from the Lutetian of Georgia [73,74]. However, *Solterichthys macrognathus* from the Ypresian of Solteri turned out to be ca. 8 million years older than the previously recorded first occurrence of a Phosichtyidae in the fossil record (*Vinciguerria distincta*; Dabakhan Formation, Georgia [132,139]) and, together with *Sabbathichthys osbournei* n. gen. et n. sp. from Monte Solane, it extends the stratigraphic range of phosichthyids back to the Ypresian [14].

Among the myctophiforms, *Eomyctophum mainardii* is one of the earliest unquestionable lanternfishes of the Cenozoic, being present in both Monte Solane and Solteri, confirming the presence of the genus *Eomyctophum* also in southern Europe [14].

The newly established taxa of euzaphlegids and gempylids from Monte Solane further expand our knowledge of the scombriform paleodiversity in the early Paleogene. However, the most significant finding is the cutlassfish *Eomastix zabimaru* n. gen. et n. sp., representing the earliest articulated skeletal evidence of a trichiurid in the record. The earliest putative fossils referred to the Trichiuridae are known from the Paleocene but are based only on isolated teeth [122], while the previously known earliest complete skeleton comes from the Lutetian of the Dabakhan Formation (Georgia; [139]).

Lastly, among the epipelagic taxa reported from Solane, *Lepidoclupea renga* n. gen. et n. sp. stands out as one of the most ancient and unquestionable round herrings (dussumieriids) together with *Trollichthys bolcensis* (upper Ypresian, Bolca, Italy; [61]).

### Climatic context of early Paleogene mesopelagic fishes of Monte Solane and Solteri

**Fig 31:**
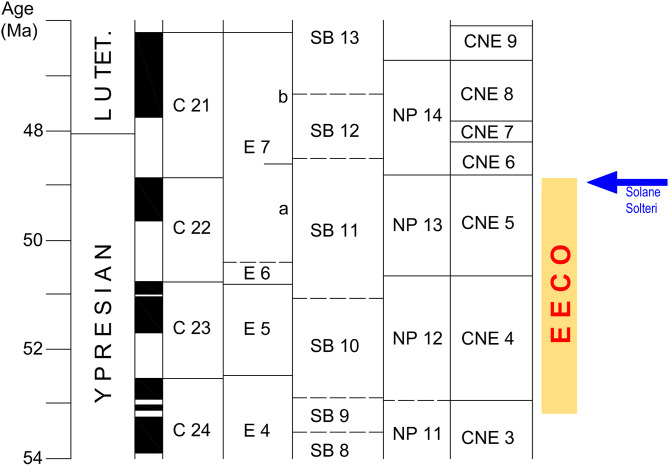
Biomagnetostratigraphy of the Ypresian p.p.-Lutetian p.p. interval. Time scale, geomagnetic polarity scale, planktic foraminiferal zonation (E), larger benthic foraminiferal zonation (SB) and calcareous nannofossil zonations (NP and CE) after Speijer et al. [45] with modifications. Planktic foraminiferal zonation E of Wade et al. [28] with modifications of Luciani & Giusberti [29], Tethyan zonation of larger benthic foraminifera SB from Serra-Kiel et al. [42] with modifications of Benedetti et al. [142], and calcareous nannofossil zonations NP of Martini [43] and CNE of Agnini et al. [41]. The yellow bar corresponds to the EECO stratigraphical position and concept according to Westerhold et al. [141]. The blue arrow indicates the stratigraphic position of the Monte Solane and Solteri fish beds.

The early Paleogene records some of the most significant climatic events of the Cenozoic, considered major drivers of the evolutionary radiation of teleost fishes [140]. Differently from the neritic environment, the oldest known Paleogene mesopelagic ichthyofaunas previously came from upper Eocene and lower Oligocene sites (e.g., Maikop Formation, Russia [95]; Litenčice, Czech Republic; Piatra-Neamț, Romania [74,97]), leaving a major gap in understanding mesopelagic contributions to this extraordinary diversification. The Monte Solane and Solteri record significantly contribute to filling this gap: both sites, here assigned to the upper part of the CNE5 and E6/E7a biozones (48.9–50.5 Ma [45]), deposited in the late Ypresian, during the final phase of the Early Eocene Climatic Optimum (EECO, ~ 53.2–49.1 Ma; Fig 31) *sensu* Westerhold et al. [141]. The prolonged warming of the EECO likely created favorable conditions for diversification not only in neritic environments but also in the mesopelagic zone [140]. However, how mesopelagic teleost assemblages were structured and evolved during the climatically dynamic early Paleogene and, specifically, through the ~ 4 Myr-long EECO, remains a major unresolved question, which can only be addressed through the discovery of progressively older bathyal sites that will bridge the 16 Myr-long gap between the base of the Paleocene and the top of the lower Eocene.

## Conclusions

With this study, we provide the first detailed description of the Monte Solane ichthyofauna, one of the most ancient mesopelagic fish assemblages of the Cenozoic so far known. Our research evidences:

- The presence of eight new bony fish taxa belonging to different mesopelagic and epipelagic groups (*Acanthophleges lessiniae* n. gen. et n. sp., *Bolcaichthys solanensis* n. sp., *Contemptor mastinoi* n. gen. et n. sp., *Eomastix zabimaru* n. gen. et n. sp., *Lepidoclupea renga* n. gen. et n. sp., *Sabbathichthys osbournei* n. gen. et n. sp., *Thyrsitoides cangrandei* n. sp., and *Veronaphleges ambrosii* n. sp.), with some being the oldest known representatives of their respective groups, with their first appearances dating back to the late Ypresian, approximately eight million years earlier than previously thought (e.g., Gonostomatidae, Phosichthyidae, and Trichiuridae).
- The integrated investigation of calcareous plankton biostratigraphy of the Solteri site of Trento allowed us to correlate its fossiliferous interval with that of Monte Solane, allowing us to establish that the two fish beds are both upper Ypresian and perfectly coeval, falling within the upper CNE 5 and E6/E7a biozones, implying that the two sites deposited in the final phase of the EECO.
- The evolutionary implications of the newly described ichthyofauna of Monte Solane allow us to better decipher the early evolution of the modern deep-sea communities, thus shedding light on one of the most understudied and underrepresented paleoenvironments in the sedimentary record.

## Supporting information

S1 Fig Stratigraphic column of the Solteri section (Trento) plotted against the calcium carbonate curve, the abundance (calculated on 6–7 mm^2^. On the left), and the relative abundance (on the right) of selected calcareous nannofossil taxa. The percentage abundance of *Coccolithus* taxa is calculated on the total number of *Coccolithus*, whereas the relative abundance of selected *Discoaster* taxa is calculated on the total number of *Discoaster* in the calcareous nannofossil assemblage. A: lithostratigraphy; B: lithologic intervals; C: planktic foraminiferal zonation E after Wade et al. [28], modified by Luciani & Giusberti [29]; D: calcareous nannofossil zonation CNE after Agnini et al. [41]. The asterisk indicates the position of the sample that yielded a single specimen of *Tribrachiatus orthostylus*. Lithologic legend: 1) laminated, organic-rich marls and calcareous marls; 2) limestones, marly limestones and marls; 3) calcarenitic limestones; 4) calciruditic larger foraminiferal limestones; 5) chert; 6) dark grey to brown lithologies; 7) fishes.
(TIF)

S1 Table Raw calcareous nannofossil data analyzed for the Solteri section, with indication of the samples studied for qualitative planktic foraminiferal analysis. (XLSX)

S2 Table Summary of the meristic traits of different gempylid genera. Includes new data and data from [14,107,112,122,143–148].
(DOCX)
