# Supplementary figures and images for: The Ypresian ichthyofauna of the Monte Solane Lagerstätte (Verona, northern Italy): A deep dive into the western Tethys early Eocene mesopelagic setting
Source: PLoS One. 2026 Mar 4;21(3):e0338490. doi: 10.1371/journal.pone.0338490 (PMC12959715; doi:10.1371/journal.pone.0338490)

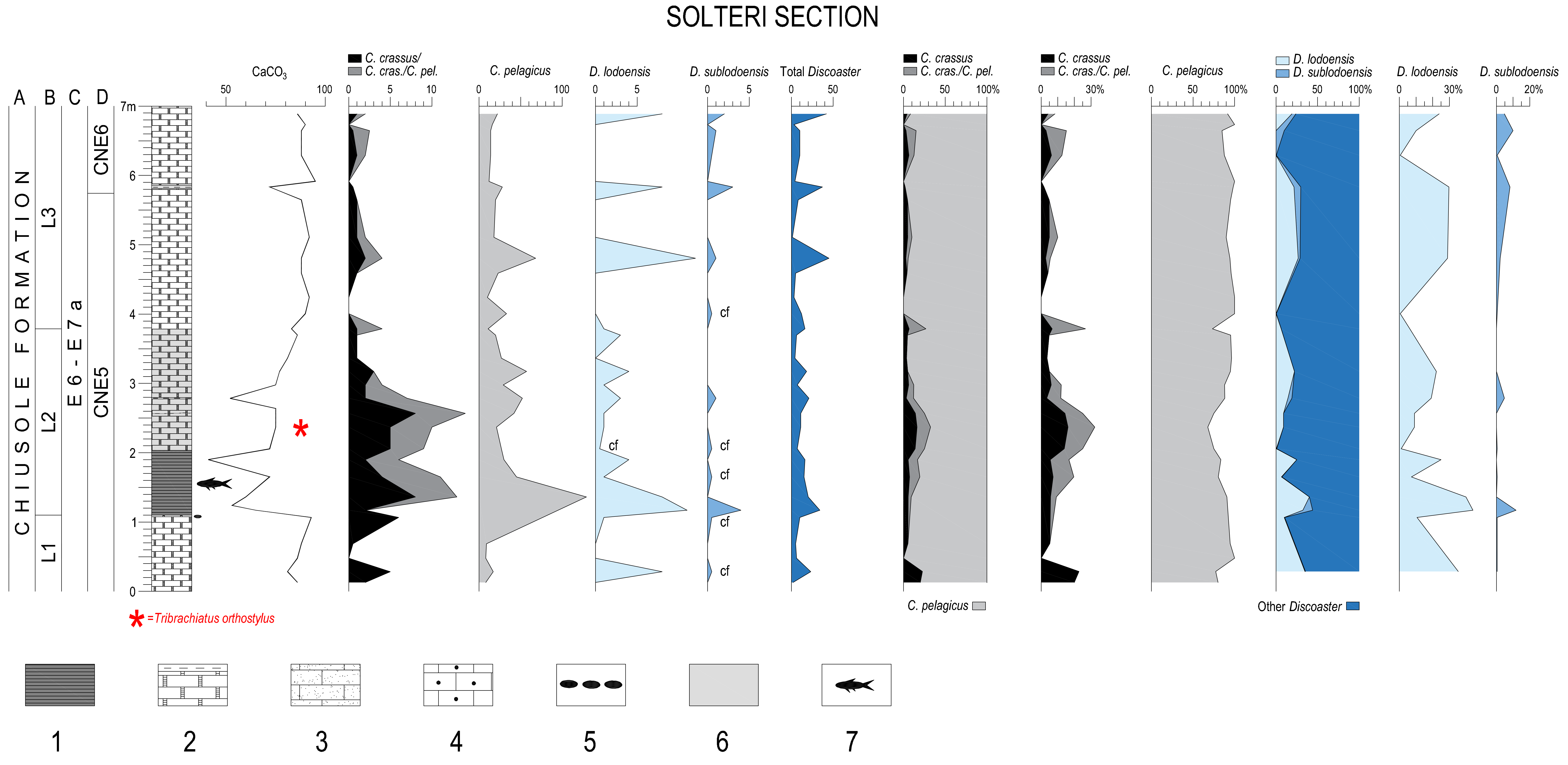

Supplement: S1 Fig — On the left), and the relative abundance (on the right) of selected calcareous nannofossil taxa. The percentage abundance of Coccolithus taxa is calculated on the total number of Coccolithus, whereas the relative abundance of selected Discoaster taxa is calculated on the total number of Discoaster in the calcareous nannofossil assemblage. A: lithostratigraphy; B: lithologic intervals; C: planktic foraminiferal zonation E after Wade et al. [28], modified by Luciani & Giusberti [29]; D: calcareous nannofossil zonation CNE after Agnini et al. [41]. The asterisk indicates the position of the sample that yielded a single specimen of Tribrachiatus orthostylus. Lithologic legend: 1) laminated, organic-rich marls and calcareous marls; 2) limestones, marly limestones and marls; 3) calcarenitic limestones; 4) calciruditic larger foraminiferal limestones; 5) chert; 6) dark grey to brown lithologies; 7) fishes. (TIF) [file pone.0338490.s001.tif]
