# Supplementary material for: The Ypresian ichthyofauna of the Monte Solane Lagerstätte (Verona, northern Italy): A deep dive into the western Tethys early Eocene mesopelagic setting
Source: PLoS One. 2026 Mar 4;21(3):e0338490. doi: 10.1371/journal.pone.0338490 (PMC12959715; doi:10.1371/journal.pone.0338490)
Supplement: S2 Table — Includes new data and data from [14,107,112,122,143–148]. (DOCX) [file pone.0338490.s003.docx]

|  | ***Contemptor mastinoi* n. gen. et n. sp.** | ***Thyrsioides cangrandei* n. sp.** | ***Abadzekhia*** | ***Argestichthys*** | ***Chelificthtys*** | ***Eothyrsites*** | ***Epinnula*** | ***Hemithyrsites*** | ***Krampusichthys*** | ***Progempylus*** |
| --- | --- | --- | --- | --- | --- | --- | --- | --- | --- | --- |
| **1st Dorsal-fin rays** | VIII+ | XVI-XVII | XVI | XIV+ | XIII | ? | XV-XVI | XVII-XVIII | IX-XIII | ? |
| **2nd Dorsal-fin rays** | I, 28 | I, 14 | I, 12 | 17 | ? | 10+? | I, 15-18 | I, 17-20 | I-II, 19-26 | ? |
| **Anal-fin rays** | II, 24 | II; 11+ | II, 13 | II, 12 | ? | ? | III, 13-17 | II, 16 | II; 14-19 | ? |
| **Pectoral-fin rays** | 15 | 15-19 | 12+ | 14-16 | 16 | 10+ | 15-16 | 13-14 | 15-19 | ? |
| **Pelvic-fin rays** | I+5 | I+5 | I+4-5 | I+5 | - | ? | I+5 | I, 0 | I+5 | ? |
| **Caudal-fin rays (procurrent)** | 6+6 | ?+6 | ? | ? | ? | ? | ? | 4+4 | 6-7+6-8 | ? |
| **Vertebrae** | 34 (15+19) | 34 (18+16) | 34 (16+18) | 35 (15+20) | 17+ | 30+ | 32-35 | 33-35  (16-20+14-16) | 32 (14+18) | ? |
| **Branchiostegal rays** | 5+? | 7 | ? | 7 | ? | ? | 7 | ? | 8 | 2+ |
| **Pmx teeth (fangs)** | 20 (2-3) | 27 (3) | 25 (1) | 16 (5) | ? | 9+ (1+) | 15-25 (2+3) | ? (0-3) | 14-21 (1-2) | ? |
| **Den teeth (fangs)** | 5 (4) | 11+ (1) | 12-13 (0) | 9 (4-5) | ? | 4+ (?) | 7-11 (2) | ? (1) | 7 (3) | 4+(?) |
